# Comprehensive mRNA Expression Profiling Distinguishes Tauopathies and Identifies Shared Molecular Pathways

**DOI:** 10.1371/journal.pone.0006826

**Published:** 2009-08-28

**Authors:** Iraad F. Bronner, Zoltán Bochdanovits, Patrizia Rizzu, Wouter Kamphorst, Rivka Ravid, John C. van Swieten, Peter Heutink

**Affiliations:** 1 Section Medical Genomics, Department of Clinical Genetics, VU University Medical Center, and Center for Neurogenomics and Cognitive Research, VU University Medical Center and VU University, Amsterdam, the Netherlands; 2 Department of Neurology, Erasmus Medical Center, Rotterdam, the Netherlands; 3 Department of Pathology, VU University Medical Center, Amsterdam, the Netherlands; 4 Netherlands Brain Bank, Amsterdam, the Netherlands; National Institutes of Health, United States of America

## Abstract

**Background:**

Understanding the aetiologies of neurodegenerative diseases such as Alzheimer's disease (AD), Pick's disease (PiD), Progressive Supranuclear Palsy (PSP) and Frontotemporal dementia (FTD) is often hampered by the considerable clinical and molecular overlap between these diseases and normal ageing. The development of high throughput genomic technologies such as microarrays provide a new molecular tool to gain insight in the complexity and relationships between diseases, as they provide data on the simultaneous activity of multiple genes, gene networks and cellular pathways.

**Methodology/Principal Findings:**

We have constructed genome wide expression profiles from snap frozen post-mortem tissue from the medial temporal lobe of patients with four neurodegenerative disorders (5 AD, 5 PSP, 5 PiD and 5 FTD patients) and 5 control subjects. All patients were matched for age, gender, ApoE-ε and MAPT (tau) haplotype. From all groups a total of 790 probes were shown to be differently expressed when compared to control individuals. The results from these experiments were then used to investigate the correlations between clinical, pathological and molecular findings. From the 790 identified probes we extracted a gene set of 166 probes whose expression could discriminate between these disorders and normal ageing.

**Conclusions/Significance:**

From genome wide expression profiles we extracted a gene set of 166 probes whose expression could discriminate between neurological disorders and normal ageing. This gene set can be further developed into an accurate microarray-based classification test. Furthermore, from this dataset we extracted a disease specific set of genes and identified two aging related transcription factors (FOXO1A and FOXO3A) as possible drug targets related to neurodegenerative disease.

## Introduction

Neurodegenerative diseases are characterised by regional cellular changes associated with progressive loss (of function) of neurons that on careful examination of brain pathology is generally distinctive between diseases. On a clinical level however, large differences exist and in addition symptoms can vary widely during the course of the disease. Although classification of patients is possible into broadly defined clinical and pathological groups, defining the borders of such classifications is often hampered by individual variation between patients, even within families with Mendelian forms of disease [e.g. as is the case in Frontotemporal dementia (FTD), [1]–[3]]. Furthermore, towards the endpoint of disease clinical characteristics of the different disorders converge, making it even more difficult to classify patients with less typical disease phenotypes [2].

Characteristic for many neurodegenerative diseases is the occurrence of characteristic aggregation of specific proteins in brain. Neurodegenerative disorders may be distinguished upon presence and absence of disease specific protein aggregates e.g. α-synuclein in Parkinson, and amyloid precursor protein in Alzheimer's disease or the microtubule associated protein tau (MAPT or tau) as observed in for example AD, FTD, PiD, PSP [4], [5]. Although in this last group of diseases, also referred to as tauopathies, patients are often distinguishable upon pathology, patients with less typical or mixed pathology are difficult to classify, demonstrating the need to understand the underlying processes behind the pathological changes.

The identification of genetic defects for Mendelian forms of neurodegenerative disorders has given important new clues about molecular pathways involved in their aetiology, and has contributed to a better classification systems for a range of these neurodegenerative disorders [3]. Although Mendelian genes usually explain only a minority of cases, their identification has opened up new research directions and this has given us important new clues about the molecular pathways involved. As the Mendelian forms of disease do not always result in uniform clinical and pathological findings, it can be hypothesised that genetic and/or environmental modifying factors contribute to the disease process. Evidence is emerging that some of the identified genes (i.e. MAPT, α-Synuclein) also play a role in the more common (non-Mendelian) forms of the disease and it is generally accepted that the aetiology of the non-Mendelian forms of neurodegenerative disorders must be explained by a combination of genetic and environmental factors and that the effects of the sum and interactions of these factors must be reflected in gene expression patterns on both the RNA and protein level.

Identification of the complete network of genetic and environmental factors involved in a disease is a daunting task. In the past, genetic association studies of candidate genes tried to identify risk factors once at a time. The full elucidation of the genetic influences of neurodegenerative diseases, however, requires a thorough understanding of the relationship between the variation present in our genome and the corresponding phenotypes. The ability to collect this genomic information, including sequence and genotype information, gene/protein expression levels and cell biological parameters, rapidly and inexpensively, has long been a bottleneck to realizing this goal. However, in the past 15 years genomic approaches have entered the functional evaluation phase and newly developed high-throughput methods now provide enormous amounts of raw data for that purpose (see e.g. [6], [7]. The ability to perform these highly parallel genomic assays depends on two fundamental characteristics of the assays: a highly parallel array based read-out and an intrinsically scalable multiplexing sample preparation. Gene-expression profiling was the first type of genomic assay to be parallelized and over the years using high-density DNA arrays for readout from a single-tube sample preparation has matured into a “standard” molecular biology tool for many research institutions. Gene expression profiling has been used for neurodegenerative disorders but mainly with the aim to identify molecular pathways affected by the disease process [8], [9]. An important criticism on these studies has been that human post-mortem brain represents an end stage of disease where a total collapse of the system might lead to many changes not relevant for the disease process, which is reflected in the observance of converging clinical symptoms. An alternative approach would therefore be to use animal models such as transgenic mice with human mutations. However, this approach only partly overcomes the problem. There is indeed an advantage that one can study the expression patterns longitudinally, however, most animal models mimic only part of the human phenotype and therefore the relevance of findings is difficult to establish

Studying end stage human brain material, although not ideal, can still provide important information and by using a different study design than commonly used (i.e. comparing a group of patients with a specific disease with a control group) the value of human material can be improved. We have therefore systematically compared four groups of patients of different disorders, with significant overlap in clinical and pathological characteristics with controls and searched for discriminating gene expression patterns as well as common denominators for more than a single disease. Furthermore, we compared our original group with a group of independent samples to confirm our obtained results were robust and reliable. This study is a first attempt to distinguish different neurodegenerative disorders with tau pathology (and related disease processes) based upon genome wide gene expression patterns.

## Results

### Clustering

We performed a hierarchical cluster analysis based on similarities in gene expression without pre-grouping of samples based upon clinical, pathological or genetic characteristics. No clustering was observed for age of death, gender, ApoE-ε and MAPT haplotype or post mortem delays (see more details about brain samples in Table 1).

**Table 1:** Overview of brain samples.

NBB number (*)	Autopsy nr	gender	age	Disease	Braak stage	Post mortem delay (h:m)	Brain pH	brain weight (grams)	APOE haplotype	region used	MAPT haplotype	Disease duration (years)	Cause of death	Chip ID	Figure ID
96–098	S96/289	f	72	Progressive supranuclear palsy		5∶20	6.25	1213	43	medial temporalis gyrus	H1/H1	7	pulmonary arrest	IB31	PSP5
96–119	S96/340	m	72	Progressive supranuclear palsy		5∶48	6.71	1160	33	medial temporalis gyrus	H1/H1	9	dehydration and respiratory insufficiency	IB29	PSP3
97–072	S97/191	m	57	Progressive supranuclear palsy		5∶43	6.42	1396	33	medial temporalis gyrus	H1/H1	6	pneumonia and cachexia by PSP	IB28	PSP2
99–050	S99/120	f	93	Progressive supranuclear palsy	2	4∶15	6.32	1048	33	medial temporalis gyrus	H1/H1	3	cachexia and dehydration	IB30	PSP4
00–086	S00/180	m	73	Progressive supranuclear palsy		8∶55	7.20	1408	42	medial temporalis gyrus	H1/H1	2	euthanasia	IB27	PSP1
00–096 *	S00/203	m	73	Progressive supranuclear palsy		5∶20	7.04	1204	43	medial temporalis gyrus	H1/H1	3	pneumonia/cachexia	IB64	
95–099 *	S95/287	f	57	Progressive supranuclear palsy		8∶10	6.75	1391	33	medial temporalis gyrus	H1/H1	4	septic with a urinary infection	IB55	
**AD**															
00–140	S00/318	f	72	Alzheimer's disease	6	3∶45	6.61	1070	43	medial temporalis gyrus	H1/H1	15	dehydration	IB35	AD4
00–099	S00/206	f	78	Alzheimer's disease	6	3∶35	6.93	935	33	medial temporalis gyrus	H1/H2	7	unknown	IB34	AD3
00–044	S00/085	f	80	Alzheimer's disease	6	5∶20	6.68	946	44	medial temporalis gyrus	H1/H1	unspecified (7+)	general deterioration	IB32	AD1
01–091	S01/206	m	73	Alzheimer's disease	6	5∶00	6.68	1075	43	medial temporalis gyrus	H1/H1	10	cachexia following swallowing disturbances	IB33	AD2
00–066	S00/141	m	80	Alzheimer's disease	6	4∶20	7.08	1240	43	medial temporalis gyrus	H2/H2	7	dehydration and vascular insufficiency	IB36	AD5
95–069 *	S95/181	m	68	Alzheimer's disease	6	4∶15	6.65	1446	44	medial temporalis gyrus	H1/H1	8	aspiration pneumonia	IB60	
89–073 *	S89/208	m	82	Alzheimer's disease	6	3∶25	6.64	1010	43	medial temporalis gyrus	H1/H1	13	cachexia and dehydration	IB59	
**CONTR**															
01–028	S01/078	f	78	Non-demented control	1	4∶50	6.40	1250	33	medial temporalis gyrus	H1/H1	-	myocardial infarction	IB39	CONTR3
00–142	S00/320	f	82	Non-demented control	1	5∶30	6.60	1280	32	medial temporalis gyrus	H1/H1	-	myocardial infarction	IB37	CONTR1
98–101	S98/196	m	72	Non-demented control		6∶45	undefined	1383	43	medial temporalis gyrus	H1/H1	-	heart failure	IB38	CONTR2
99–116	S99/249	m	78	Non-demented control		4∶20	undefined	1310	33	medial temporalis gyrus	H1/H1	-	pancreatic cancer	IB40	CONTR4
01–021	S01/064	m	82	Non-demented control	1	7∶40	6.07	1373	33	medial temporalis gyrus	H1/H2	-	heart attack	IB41	CONTR5
**Pick C1**															
96–064	S96/179	m	78	Pick's disease (Const. type C1)		5∶00	6.64	1354	43	medial temporalis gyrus	H1/H1	12	sepsis, due to an urinary tract infection, and cachexia	IB17	FTD1
03–049	S03/137	f	64	Pick's disease (Const. type C1)		5∶10	6.53	1002	43	medial temporalis gyrus	H1/H1	3	respiratory insufficience	IB18	FTD2
96–094	S96/277	f	72	Pick's disease (Const. type C1)		3∶55	6.90	1243	43	medial temporalis gyrus	H1/H1	8	cachexia and dehydration	IB19	FTD3
98–029	S98/046	m	74	Pick's disease (Const. type C1)		5∶10	6.49	1223	33	medial temporalis gyrus	H1/H1	14	infection of lungs	IB20	FTD4
98–119	S98/223	m	74	Pick's disease (Const. type C1)		3∶45	6.55	951	43	medial temporalis gyrus	H1/H2	11	pneumonia in a dehydrated and cachexic patient	IB21	FTD5
97–051 *	S97/158	m	75	Pick's disease (Const. type C1)		5∶30	7.50	1069	33	medial temporalis gyrus	H1/H2	12	bronchopneumonia	IB63	
**Pick A**															
98–075	S98/162	f	77	Pick's disease (Const. type A)		5∶20	6.21	967	33	medial temporalis gyrus	H1/H2	4	complications secondary to a cerebro vasculair accident	IB22	PID1
98–159	S98/283	f	83	Pick's disease (Const. type A)		5∶10	6.90	715	43	medial temporalis gyrus	H1/H1	16	acute cardiac arrest	IB23	PID2
95–086	S95/244	m	64	Pick's disease (Const. type A)		4∶40	6.53	1353	33	medial temporalis gyrus	H1/H1	6	myocardial infarction	IB24	PID3
02–062	S02/185	m	70	Pick's disease (Const. type A)		5∶15	6.53	1201	33	medial temporalis gyrus	H1/H2	15	aspiration pneumonia	IB25	PID4
96–012	S96/027	f	64	Pick's disease (Const. type A)		7∶55	6.50	1260	33	medial temporalis gyrus	H1/H2	6	bleeding of the stomach	IB26	PID5
98–163 *	S98/284	f	61	Pick's disease (Const. type A)		6∶15	6.18	1148	44	medial temporalis gyrus	H1/H2	8	lungdisorder with fever for 4 weeks	IB62	

Overview of characteristics of all brain samples used in this paper. ^*^Samples that were used for the verification experiment.

**Figure 1:**
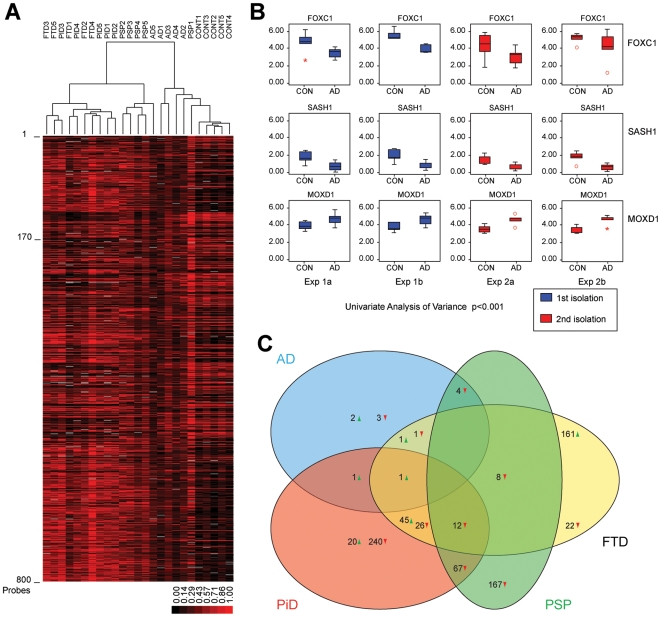
Initial analysis and confirmation of the microarray data. Figure 1A. Clustering of all 25 samples using the background filtered dataset of 790 probes. Figure 1B. Results of quantitative PCR experiments for 3 genes (FOXC1, SASH1 and MOXD1) that were significantly differently expressed in Alzheimer's disease vs. controls. Experiments were done in duplo (a and b) using cDNA from two separate isolations of the same brain sample (Exp 1 and 2). Statistical testing was done using a Univariate Analysis of Variance with p<0.001 in all samples. Similar results were observed in the other tested illnesses. Figure 1C. Graphical overview of probes and their correlation between pathology defined groups. Amount of up- and down-regulated probes is shown with up- or downwards facing arrowheads. Pathology defined groups are represented as coloured ellipses; Alzheimer's disease (AD): blue, Pick's disease (PiD): red, Frontotemporal dementia (FTD), yellow and Progressive Supranuclear Palsy (PSP): green.

Considerable overlap in expression patterns for samples with distinct clinical and pathological findings was detected even though most Frontotemporal lobe degeneration [26] samples (i.e. FTD and PiD samples) clustered separately from the other samples (results not shown). A straightforward distinction based on existing clinical and pathological criteria between all tested neurodegenerative diseases is therefore not immediately obvious from these gene expression data. Possible explanations for this ambiguous clustering may be variation in lifetime environmental influences (e.g. life style or the conditions under which the patients had died) and genomic-background effects for individual patients, which would contribute to population expression variation (i.e. noise). Whereas an alternative explanation would be that the neurodegenerative disorders fail to influence the expression of the majority of genes between sample groups. In order however to restrain the influence of normal variation we used the gene expression data from the control group as reference. Using significance analysis of microarrays (SAM) [22] we then compared each clinically and pathologically defined patient group to the control group and identified those genes that were expressed significantly different from control levels (see Table S1). Microarray results for significantly changed genes were confirmed by quantitative reverse transcriptase PCR (qRT-PCR) (Figure 1B). Interestingly the expression levels of several neurodegeneration related genes (i.e. MAPT, GRN, APP or PSEN1 and PSEN2) showed differences from the background levels in all clinical and pathological related groups, but failed to reach significance due to large individual variability (results not shown).

This analysis defined a new data set consisting of 790 significantly altered probes (∼730 genes; Table S2) between patients and controls. Re-clustering using this reduced gene set resulted in separation of samples into clinically and pathology-related groups (see Figure 1A). All controls and 4 of the 5 PSP patients formed separate clusters. The FTD and PiD disease samples clustered as a single large group and four of five Alzheimer samples also clustered together. However, an Alzheimer sample (AD5) grouped with the PSP group (see Figure 1A). Interestingly, we observed (Table S1 and Figure 1C) that the AD samples and control samples were highly similar (only 11 differently expressed genes) in gene expression. Although this seems unexpected, it might be explained by an observation made by drs. K. Bossers and J. Verhaagen (pers. comm.) that high variability of gene expression between different samples was seen in earlier and later Braak stages, whereas in intermediate Braak stages (stage III and IV) expression was more similar. Because expression levels were more variable between our patients, it becomes more difficult for the permutation analysis to pick up significant gene expression changes.

We further observed (Figure 1C) that the majority of genes in the reduced data set of 790 probes that were significantly differently expressed from the control group, were specific for single patient groups. To identify common denominators for the different diseases we reanalysed the dataset by including only those genes that were significantly altered in at least two patient groups. This reduced the gene set to only 166 probes (Table S3). Interestingly we observed no common gene significantly altered in all patient groups.

**Figure 2:**
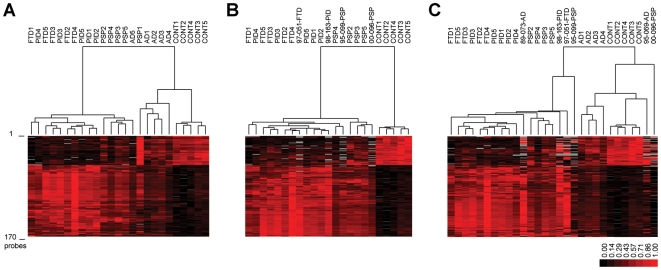
Analysis of original and new samples using the refined microarray data set. Figure 2A. Clustering of all 25 samples using the refined dataset of 166 probes. Figure 2A. Clustering of selected 23 samples and new PSP, FTD and PiD disease samples. Figure 2C. Clustering of selected 23 samples and new PSP, FTD and PiD - and Alzheimer's disease samples. Sample names are as in Figure 1.

Using this more stringent gene set, clustering further improved the separation of samples in clinically and pathologically defined groups, especially for the AD, PSP and control samples (see Figure 2A). Surprisingly FTD and PiD disease samples still clustered as a single large group. Although the considerable overlap in gene expression might be explained by the overlap in clinical symptoms and affected brain regions, the group of FTD patients do not show extensive tau pathology as they were classified as Constantinidis type C1 while the PiD patients all showed extensive tau pathology (Constantinidis type A) suggesting that the observed tau pathology is not necessarily indicative for the molecular pathways affected. We noted that the FTD and PiD samples only showed overlap for a small subset of genes significantly different from control group levels, whereas for the majority of genes in the dataset expression seemed unrelated (see Figure 1C). Since clustering of the two sample groups was very robust, this suggested a strong correlation in gene expression between the two sample groups. Therefore we constructed correlation plots between the 166 probes and the 790 probes of the larger dataset, and calculated correlation coefficients. The correlation coefficient between both illnesses was approximately 1 (data not shown) confirming that PiD disease and FTD are closely related disorders based on clinical, pathological and mRNA gene expression findings.

Although the majority of samples clustered within their clinically and pathologically related sample group, two samples (AD5 and PSP1) did not (see Figure 2A). Since, gender, age at death, ApoE-ε and MAPT haplotype could not explain why gene expression in these samples was distinct from related samples, we evaluated the medical history of these samples in more detail.

Sample PSP1 showed a somewhat longer post mortem delay than the average of our samples (8.55 hours; average for samples 5:15 hours); however RNA quality, noise levels and the number of detected probes on the microarrays did not differ form other samples (see Table S4[A and B]). Although this sample was pathologically classified as PSP, the patient originally was clinically diagnosed with corticobasal degeneration, which shows considerable phenotypic overlap with PSP [27]. Furthermore, this patient had died following euthanasia (Nesdonal 10 mg i.v., Pavulon 1000 mg i.v.), which indicates a substantial period of unbearable suffering for the patient prior to this medication and this might have had an influence on RNA expression patterns. We therefore excluded this sample from further analysis.

Sample AD5 was from an Alzheimer's disease patient who was reported to suffer from epileptic seizures and motoric disturbances. These additional clinical findings might explain why this case did not group with the other AD cases. Because of these atypical findings this patient was excluded from further analysis.

In conclusion, using a stringent gene set consisting of 166 probes it was possible to discriminate controls, AD and PSP cases from each other and clearly discriminates these groups from the group of FTD/PiD disease samples, i.e. a FTLD [26] group.

We then tested whether this dataset would also be valid in an independent group of patients. We acquired brain material from 6 additional cases: two AD (Braak stage 6), two PSP and two Pick/FTD samples [i.e. one sample with classical PiD disease (Constantinidis type A), and one with FTD (Constantinidis type C1)]. All samples matched the ones from the earlier experiment. RNA was isolated, labelled and run on new Affymetrix GeneChip Human Genome U133 Plus 2.0 microarrays by the LGTC according to protocol. All microarray data was normalised using GeneChip Operating Software (GCOS) 3.0 (Affymetrix, Inc., Santa Clara, California, USA).

Considering the results of our first experiment we first performed cluster analysis using the set of 790 significantly different expressed probes. To correct for inter-experimental error, all 790 probes were standardised between the two experiments by dividing the 790 genes from the original dataset and the new dataset by the average expression ratio per probe in Microsoft excel (Microsoft Corporation, Redmond WA, USA). Since the second set of samples did not include controls, standardisation was only done using patient samples from the first dataset. This correction is necessary since the ratio of gene expression per probe from the first 20 experiments and the latter 6 experiments was on average 1.5 times higher (data not shown). Next, expression of independent genes (Exp_n_) was transformed to fit between 1 and 0 using the minimal (Exp_min_) and maximal expression (Exp_max_) according to following formula: (Exp_n_−Exp_min_/(Exp_max_−Exp_min_)), and hierarchical cluster analysis was done using Cluster3.

Since expression data for the 166 overlapping probes gave best results to discriminate the different neurodegenerative disorders (Figure 2A) however, we extracted from this larger dataset the corrected expression data for the 166 probes determined previously. Examining only the new PSP and FTD/PiD disease samples in the cluster analysis with this 166 probes containing dataset, confirmed that this probe-set could discriminate PSP, FTD/PiD disease from controls based on gene expression (see Figure 2B). However when new Alzheimer's disease samples were also included, they did not cluster together with matching samples from the previous experiment, see Figure 2C. As mentioned above, this was hardly surprising since we observed very few differences between the AD and control samples in our first experiment, which might be explained by the observation (see e.g. Figure 1A) that AD patients with Braak stage 6 have heterogeneous gene expression, which we suggest might be caused by the severe neurodegeneration present in this region.

In conclusion, the current identified set of 166 genes is suitable to distinguish PSP samples from controls and FTD/PiD disease samples upon expression, furthermore, our results show that FTD/PiD disease samples behave as one group that can be distinguished from PSP samples and non demented controls.

### Pathway analysis

Since our gene expression data could distinguish patient samples from non-demented controls and from each other into pathology related groups we explored whether our data would also be suitable for identifying disease-related molecular pathways.

We used the “Ingenuity Pathways Analysis” on a subscription, web-delivered application that enables biologists to discover, visualize, and explore networks relevant to their experimental results such as gene expression array datasets. The Ingenuity database contains literature based manually curated gene ontology data, which decreases the hypothetical character of detected interactions and the validity of the interactions can be re-assessed from literature. In addition, the Ingenuity Pathway Analysis software grants the possibility to determine likelihood of observed changes using statistical analysis (see materials and methods for details). To increase reliability of the identified interactions even more we also limited ourselves to known canonical pathways.

Since our results showed that especially the PSP samples could be robustly distinguished from the other diseases, we focussed our analysis on PSP and investigated how disease related alternatively expressed genes in this disease group influence canonical pathways. To determine pathways that were significantly linked to the illness we created two separate datasets with genes that were statistically different between PSP cases and controls. We chose to calculate two separate datasets using SAM (one not stringent, and one stringent) to circumvent the issues that a stringently ascertained dataset might miss relevant pathways (because too few genes are entered in the analysis) or that a larger, less stringently selected set of genes would contain too many false positives. We chose to define only those pathways as interesting when detected in both analyses.

The low stringency set was defined by setting the significance threshold such that we accept ∼40 median false positives in the SAM analysis. Since our data contained approximately 40.000 probes this approach corresponds to using a nominal p-value of 0,001. The resulting gene set contained approximately 1600 probes with a false discovery rate (FDR) of 0.025. On the other hand, for the high stringency set we accepted only 1 median false positive. The resulting gene set contained 266 probes at an FDR of 0.004.

**Figure 3:**
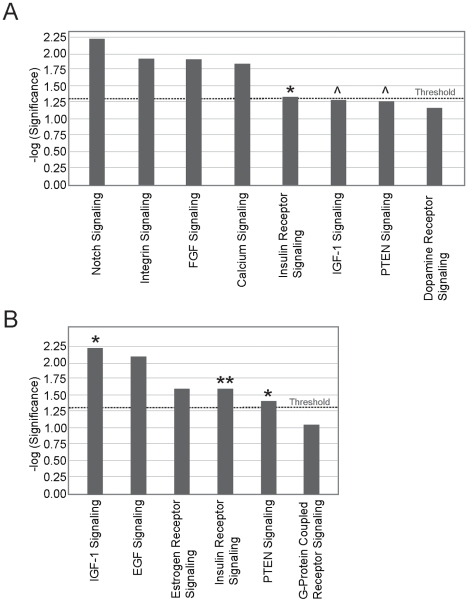
Significantly changed canonical pathways in PSP brain. Figure 3A. Significantly changed canonical pathways in PSP brain in the uncorrected dataset. Threshold for significance is shown in the dotted line. * signifies the significantly deregulated (p = 0.047) Insulin Receptor Signalling pathway and ^ signifies the almost significantly deregulated (p = 0.051 and p = 0.055) IGF-1 and PTEN Signalling pathways respectively. Figure 3B. Significantly changed canonical pathways in PSP brain in the corrected dataset. Threshold for significance is shown in the dotted line. ** signifies the significantly deregulated (p = 0.025) Insulin Receptor Signalling pathway for both analysis and * signifies the significantly deregulated (p = 0.006 and p = 0.039) IGF-1 and PTEN Signalling pathways, respectively.

To determine the likelihood for a pathway to be affected we used the statistics of the Ingenuity Pathway Analysis. This analysis compares the number of user-specified genes that participate in a pathway, relative to the total number these genes in all pathway annotations stored in the Ingenuity Pathways Knowledge Base, and gives significance values using the right-tailed Fisher's Exact Test, to indicate which pathways seem affected by the disease process. Using the less stringent dataset, five pathways were detected to be affected, among which the insulin receptor signalling (p = 0.047) (see Figure 3A). A further two pathways showed a trend toward significance (i.e. IGF-1 [p = 0.051] and PTEN [p = 0.055]; see Figure 3A).

Next, to confirm these results and to determine relevant target genes in these pathways, we repeated the analysis with a stringency of 1 median false positive per dataset (implicating essentially all detected genes to be disease related) to increase confidence that pathways were disease related. With this dataset we obtained again five affected pathways. Three of earlier mentioned pathways were detected to be significantly affected (i.e. insulin receptor signalling [p = 0.025]; IGF-1 [p = 0.006] and PTEN [p = 0.039]; see Figure 3B), and in addition the EGF and Estrogen receptor signalling pathways. The observation that several of the initially detected pathways did not remain significant in our more stringent analysis suggests that they could be false positive findings because of inclusion of house keeping processes in the initial dataset and over-detection of small and less defined pathways in the more stringent dataset. All three pathways show considerable overlap in gene content. Interestingly, the insulin/IGF-1 signalling (IIS) pathway has an important role in the ageing process [28], [29] and the transcription factors FOXO1A and FOXO3A, previously implicated as key regulators of the ageing process [29]–[33], were significantly up-regulated in our PSP data set.

To determine whether the upregulation of FOXO1A and FOXO3A was specific for PSP, we tested whether these genes were also differentially expressed in the other disease groups. In FTD samples both FOXO1A and FOXO3A were indeed also significantly upregulated, whereas in PiD only FOXO3A was significantly upregulated. In Alzheimer's disease samples no FOXO factor upregulation was observed, however as mentioned the AD samples and controls vary only on 11 genes, and the power to detect biologically relevant changes might be limited. This however might mean that FOXO3A might be a common neurodegeneration factor, whereas FOXO1A might be associated with both PSP and FTD.

## Discussion

Our results demonstrate that it possible to separate patients with pathologically confirmed PSP, AD and FTD/PiD from each other based on their gene expression patterns. In addition, we identified a gene set of 166 genes that might be developed into a tool to aid in post-mortem classification of patients. Although we identified genes that were differently expressed in more than one patient group we did not find differentially expressed genes common to all patient groups, demonstrating that although there is considerable overlap between these disorders different molecular pathways are affected. However, our analysis might have been overly stringent. We used this high stringency to minimize false positive findings. We did not reanalyse the data using lower stringency but instead aim in future experiments to extend the current study to additional brain regions and samples.

Analysis of human post-mortem brain material might be complicated by expression changes because of events just prior to death. To control for such artefacts, we compared gene expression patterns with those from control brains. Interestingly we found considerable overlap in gene expression between our Braak stage 6 AD cases and controls. We hypothesise these findings might be explained by the severity of neurodegeneration in this region and should be confirmed by including AD samples with different Braak stages or less affected brain regions.

Our results on samples with frontal forms of dementia (i.e. FTD and PiD) show that these cases not only have considerable overlap in clinical symptoms and affected brain regions but also in gene expression (see Figure 2A and B) confirming the validity to group them under the general term Frontotemporal lobar degeneration (FTLD) [26]. However to rule out that processes correlated with severity of neurodegeneration are the reason of the similarity in gene expression observed in FTD and PiD disease, our results should be validated in other brain regions. Similarly, including differently affected regions may provide more details why some regions are more sensitive to neurodegeneration and which processes are involved.

The observation that the insulin/IGF-1 signalling pathway is affected (although not equally) in PSP, FTD and PiD makes this pathway an interesting target for further research. The function of FOXO transcription factors is diverse and depends on activity of other transcription factors and cell type. FOXO transcription factors influence a diverse group of cellular mechanisms, including glucose metabolism [34], cell cycle [35]–[39], cell differentiation [40], [41], regulation of apoptosis [42]–[45] or decrease of reactive oxygen species (ROS) [36], [38], [39]. Therefore depending on signal and cell type they balance stress resistance, growth and cell death. For these reasons FOXO transcription factors can be best defined as regulators of cell fate [46].

Their mode of action however largely depends on interactions with upstream pathways, since these regulate FOXO phosphorylation, acetylation or ubiquitinilation [47], which influences FOXO stability and interaction with downstream promoters. Therefore characterisation of the upstream pathway influences cell outcome. In our microarray experiments an up regulation of IGF1-R and RAF1 was observed. Synergy between PI3K/Akt and RAF/MEK/ERK pathways has been described to prevent apoptosis and is therefore protective [48].

FOXO proteins have been reported to decrease ROS by increasing the radical scavenging proteins Mn superoxide dismutase (MnSOD) and catalase [36], [38], [39]. In several neurodegenerative disorders oxidative stress has been suggested to be correlated with disease aetiology, and therefore may provide a possible explanation. Recently in *C. elegans* a link between aggregation-mediated toxicity and decreased insulin/IGF-1-like signalling was shown. Downstream transcription factors (heat shock factor-1 and Daf-16) regulated (dis-)aggregation activities to promote cellular survival in response to constitutive toxic protein aggregation [49]. Therefore, this pathway might provide a mechanistic link to aggregation-mediated proteotoxicity and neurodegeneration.

These results imply that instead of focussing on a single gene only, future experiments to study the involvement of (e.g. these FOXO) factors in PSP, FTD, PiD and other neurodegenerative diseases should be focussed on whole pathways and their interacting pathways, instead of focussing on single genes in order to obtain a more complete insight into the true involvement of these factors in neurodegeneration.

### Ethics Statement

In agreement with Dutch law, no ethics statement is required. All research involving human brain material, however was conducted according to the ethical declaration of the Netherlands Brain Bank (see Supplementary File S1).

## Materials and Methods

### Brain samples

Snap frozen brain material from 4 pathologically defined disease groups (i.e. PSP, PiD disease (Constantinidis type A), FTD (Constantinidis type C1; dementia lacking distinctive histology)[10], AD (Braak stage 6) [11]) and controls were obtained from the Netherlands brain bank. To obtain sufficient statistical power each groups consisted of 5 samples. Because there are considerable regional differences in pathology between disease groups we selected to use the medial temporal lobe to determine gene expression patterns in all patients, as this is an affected region for all patients. All brain samples were matched by age (all groups p>0.05, t-test), post mortem delay (all groups p>0.05, t-test), gender (in total 13 males and 12 females and every group consisted of 2 or 3 males), MAPT haplotype (p>0.05, Fischer exact test) and ApoE-ε haplotype (p>0.05, Fischer exact test). Although all patients were selected to be non-familiar, all patients were screened for Microtubule Associated Protein Tau (MAPT) and Progranulin (GRN) mutations and MAPT haplotyping. DNA was isolated from brain using the Gentra PUREGENE DNA purification kit (QIAGEN Benelux B.V., Venlo, The Netherlands) according to protocol with the exception that all buffer amounts per mg of brain tissue were doubled due to the high protein and fat content of brain material. Mutation and genotype analysis was performed as described before [12]–[14].

In our confirmation experiment two additional PSP patients, two Alzheimer's disease patients (Braak stage 6 [11]), one FTD patient (Constantinidis type C1) and one PiD disease patient (Constantinidis type A [10]) were included and matched according to the same inclusion criteria.

### RNA isolation, quality assessment and labelling

Post-mortem delay might influence RNA quality [15]–[21]. Therefore to obtain RNA of adequate quality we selected patient samples with a relatively short post-mortem delay (on average 5:15 hours with the largest being 8:55 hours). RNA was isolated from 100–200 mg snap frozen brain material that had been stored at −80°C. RNA was isolated in groups of four samples, to prevent degradation during the isolation procedure, using 2–4 ml RNA-Bee (Iso-Tex diagnostics Inc, Friendswood, Texas, USA) and purified using the RNeasy RNA cleanup kit (QIAGEN Benelux B.V., Venlo, The Netherlands) according to manufacturer's protocol. It was then quantified using a spectrophotometer and quality was assessed using an Agilent 6000 Nano bioanalyser chip (Agilent Technologies Netherlands B.V., Amstelveen, The Netherlands). RNA samples were only included when the ratio between 28 s and 18 s rRNA was higher than 0.5 and contributed to more than 15% of total mRNA. An average sample, as determined by results from the Agilent 6000 Nano bioanalyser chip, was labelled and run on an Affymetrix GeneChip® Test3 Array (Affymetrix, Inc., Santa Clara, California, USA) to determine RNA quality and labelling. Ten µg of total RNA was transcribed into cDNA and subsequently into biotin labelled complement RNA (cRNA) using the Affymetrix one cycle target labelling kit (Affymetrix, Inc., Santa Clara, California, USA). All samples were hybridised by the Leiden Genome Technology Center (LGTC, Leiden, The Netherlands) on Affymetrix GeneChip Human Genome U133 Plus 2.0 microarrays according to protocol. Quality control showed that the amount of detected probes was on average between 40 and 50 percent (see Table S4A); furthermore detectable noise levels were low (see Table S4B) and thus RNA degradation was low. Therefore all our samples were of sufficient quality to proceed.

### Microarray analysis, clustering and pathway analysis

All microarray data have been deposited in ArrayExpress, the public archive for transcriptomics and related data at the EBI (http://www.ebi.ac.uk/microarray-as/ae/; accession number: E-MEXP-2280), and can be accessed through the following direct link: http://www.ebi.ac.uk/microarray-as/ae/browse.html?keywords=E-MEXP-2280. All microarray data was normalised using GeneChip Operating Software (GCOS) 3.0 (Affymetrix, Inc., Santa Clara, California, USA). Only marginal (0.06<p<0.04) and present (p<0.04) probe sets were used for subsequent analyses. Data analysis was done using the statistical package Significance Analysis of Microarrays (SAM) [22] in R (http://www.r-project.org) since previous results had shown this test to give valid results [23], [24]. Permutation analysis on a test set showed a minimum of 300 permutations needed to be performed for consistent results. The low stringency set was defined by setting the significance threshold such that we accept ∼40 median false positives in the SAM analysis. Since our data contained approximately 40.000 probes this approach corresponds to using a nominal p-value of 0,001. The stringent dataset was obtained by increasing the significance threshold to obtain less than 1 median false positive per group.

To correct for inter-experimental error between original and control datasets, all 790 probes obtained in the stringent analysis were standardised between the two experiments by dividing expression values of the 790 genes from the original dataset and the new dataset by the average expression ratio of the two datasets per probe in Microsoft excel (Microsoft Corporation, Redmond WA, USA). Since the second set of samples did not include controls, standardisation was only done using patient samples from the first dataset. Next, expression of independent genes (Exp_n_) was transformed to fit between 1 and 0 using the minimal (Exp_min_) and maximal expression (Exp_max_) according to following formula: (Exp_n_−Exp_min_/(Exp_max_−Exp_min_)), and hierarchical cluster analysis was done using Cluster3 [25].

Significance of analysed pathways was calculated in Ingenuity (www.ingenuity.com) using the “Functional Analysis” option. The significance value associated with this Analysis for a dataset is a measure for the likelihood genes from the dataset file under investigation participate in that function. The significance is expressed as a p-value, which is calculated using a right-tailed Fisher's Exact Test. The p-value is calculated by comparing the number of user-specified genes of interest that participate in a given function or pathway, relative to the total number of occurrences of these genes in all functional/pathway annotations stored in the Ingenuity Pathways Knowledge Base (83 in IPA version 3.5; [explanation adapted from official text IPA website]).

### Quantitative PCR and Primers

To confirm microarray results, cDNA was made according to standard protocols at 50°C using superscript III, (Invitrogen, Breda, The Netherlands). To confirm microarray data, primers for MOXD1; ZNF589; FOXC1; SASH1; ACAD2; SEPT2; PNPLA2; TNPO1; CBL; GOLPH4; NRD1; PARD3; PTEN; NPIP; BTBD14A; CENTB5; HPRT and β-ACTIN were designed for quantitative PCR using Primer Express 2.0.0 software (Applied Biosystems, Foster City, California, USA). All qPCRs were standardized to β-Actin and HPRT signals that were run in parallel (for all primer sequences see Table S5). PCRs were performed on an Applied Biosystems 7900HT Fast Real-Time PCR System (Applied Biosystems, USA) using standard settings.

## Supporting Information

Table S1 Overview of genes detected to be significantly different from background levels determined using non-demented controls. Amounts of significant different probes are given per pathology-defined group. Alzheimer's disease: AD, Pick's disease: PiD, Frontotemporal dementia: FTD and progressive supranuclear palsy: PSP. When possible probes are named using official gene symbol names. Gene symbol: official genbank gene symbol. Gene Title: official genbank gene name. ---: Unknown
(1.12 MB DOC)

Table S2 Compiled table of all 790 probes detected to be significantly different from background and overlapping in at least two different pathologically defined groups. When possible probes are given in official gene symbol names. Gene symbol: official genbank gene symbol. Gene Title: official genbank gene name. ---: Unknown
(0.83 MB DOC)

Table S3 Compiled table of all 166 probes detected to be significantly different from background and overlapping in at least two different pathologically defined groups. When possible probes are given in official gene symbol. Gene Title: official genbank gene name. ---: Unknown
(0.20 MB DOC)

Table S4 Overview of detected probes, signal strengths, background and noise levels.
(0.17 MB DOC)

Table S5 Primer sequences of primers used for quantitative reverse transcriptase confirmation of the microarray data.
(0.04 MB DOC)

Supplementary File S1 Ethical Declaration of The Netherlands Brain Bank
(0.09 MB PDF)
