# Supplementary material for: Comprehensive mRNA Expression Profiling Distinguishes Tauopathies and Identifies Shared Molecular Pathways
Source: PLoS One. 2009 Aug 28;4(8):e6826. doi: 10.1371/journal.pone.0006826 (PMC2729393; doi:10.1371/journal.pone.0006826)
Supplement: Table S1 — Overview of genes detected to be significantly different from background levels determined using non-demented controls. Amounts of significant different probes are given per pathology-defined group. Alzheimer's disease: AD, Pick's disease: PiD, Frontotemporal dementia: FTD and progressive supranuclear palsy: PSP. When possible probes are named using official gene symbol names. Gene symbol: official genbank gene symbol. Gene Title: official genbank gene name. ---: Unknown (1.12 MB DOC) [file pone.0006826.s001.doc]

| **Alzheimer's disease** | | green = down; red = up | |
| --- | --- | --- | --- |
| Probe Set ID | Gene Symbol | Gene Title | Expression |
| 1552957_at | LOC200383 | similar to Dynein heavy chain at 16F | down |
| 1553995_a_at | NT5E | 5'-nucleotidase, ecto (CD73) | down |
| 1558009_at | SLC1A2 | solute carrier family 1 (glial high affinity glutamate transporter), member 2 | down |
| 205839_s_at | BZRAP1 | benzodiazapine receptor (peripheral) associated protein 1 | down |
| 209708_at | MOXD1 | monooxygenase, DBH-like 1 | down |
| 219968_at | ZNF589 | zinc finger protein 589 | down |
| 1553613_s_at | FOXC1 | forkhead box C1 | up |
| 1565692_at | --- | CDNA FLJ40647 fis, clone THYMU2017522 | up |
| 213641_at | ZNF500 | zinc finger protein 500 | up |
| 222760_at | ZNF703 | zinc finger protein 703 | up |
| 233901_at | --- | MRNA full length insert cDNA clone EUROIMAGE 163507 | up |
| 242611_at | --- | Transcribed locus | up |
| 41644_at | SASH1 | SAM and SH3 domain containing 1 | up |
|  |  |  |  |
| **Frontotemporal Dementia** | | | |
| Probe Set ID | Gene Symbol | Gene Title | Expression |
| 1552256_a_at | SCARB1 | scavenger receptor class B, member 1 | down |
| 1552301_a_at | CORO6 | coronin 6 | down |
| 1552739_s_at | ST7L | suppression of tumorigenicity 7 like | down |
| 1553150_at | AOF1 | amine oxidase (flavin containing) domain 1 | down |
| 1553479_at | TMEM145 | transmembrane protein 145 | down |
| 1553565_s_at | DDAH1 | dimethylarginine dimethylaminohydrolase 1 | down |
| 1553796_at | FLJ30594 | hypothetical locus FLJ30594 | down |
| 1554474_a_at | MOXD1 | monooxygenase, DBH-like 1 | down |
| 1554679_a_at | LAPTM4B | lysosomal associated protein transmembrane 4 beta | down |
| 1554747_a_at | SEPT2 | septin 2 | down |
| 1555491_a_at | FLJ11286 | hypothetical protein FLJ11286 | down |
| 1555827_at | CCNL1 | Cyclin L1 | down |
| 1556551_s_at | SLC39A6 | solute carrier family 39 (zinc transporter), member 6 | down |
| 1558009_at | SLC1A2 | solute carrier family 1 (glial high affinity glutamate transporter), member 2 | down |
| 1558010_s_at | SLC1A2 | solute carrier family 1 (glial high affinity glutamate transporter), member 2 | down |
| 1558041_a_at | LOC653319 | hypothetical protein LOC653319 | down |
| 1558796_a_at | LOC728052; LOC731255 | hypothetical protein LOC728052; hypothetical protein LOC731255 | down |
| 1559965_at | --- | CDNA clone IMAGE:4811567 | down |
| 1560659_at | --- | --- | down |
| 1560661_x_at | --- | --- | down |
| 1562583_s_at | LOC646405; LOC651239 | hypothetical LOC646405; hypothetical protein LOC651239 | down |
| 1566472_s_at | RETSAT | retinol saturase (all-trans-retinol 13,14-reductase) | down |
| 200041_s_at | BAT1 | HLA-B associated transcript 1 | down |
| 200076_s_at | C19orf50 | chromosome 19 open reading frame 50 | down |
| 200754_x_at | SFRS2 | splicing factor, arginine/serine-rich 2 | down |
| 200778_s_at | SEPT2 | septin 2 | down |
| 200868_s_at | ZNF313 | zinc finger protein 313 | down |
| 200898_s_at | MGEA5 | meningioma expressed antigen 5 (hyaluronidase) | down |
| 200946_x_at | GLUD1 | glutamate dehydrogenase 1 | down |
| 200980_s_at | PDHA1 | pyruvate dehydrogenase (lipoamide) alpha 1 | down |
| 201014_s_at | PAICS | phosphoribosylaminoimidazole carboxylase, phosphoribosylaminoimidazole succinocarboxamide synthetase | down |
| 201103_x_at | LOC728936; LOC728980; NBPF10; NBPF11; NBPF15; NBPF8 | neuroblastoma breakpoint family, member 11; neuroblastoma breakpoint family, member 15; neuroblastoma breakpoint family, member 10; neuroblastoma breakpoint family, member 8; similar to CG10522-PA; hypothetical protein LOC728980 | down |
| 201116_s_at | CPE | carboxypeptidase E | down |
| 201135_at | ECHS1 | enoyl Coenzyme A hydratase, short chain, 1, mitochondrial | down |
| 201185_at | HTRA1 | HtrA serine peptidase 1 | down |
| 201349_at | SLC9A3R1 | solute carrier family 9 (sodium/hydrogen exchanger), member 3 regulator 1 | down |
| 201439_at | GBF1 | golgi-specific brefeldin A resistance factor 1 | down |
| 201559_s_at | CLIC4 | chloride intracellular channel 4 | down |
| 201619_at | PRDX3 | peroxiredoxin 3 | down |
| 201818_at | AYTL2 | acyltransferase like 2 | down |
| 202026_at | SDHD | succinate dehydrogenase complex, subunit D, integral membrane protein | down |
| 202087_s_at | CTSL1 | cathepsin L1 | down |
| 202281_at | GAK | cyclin G associated kinase | down |
| 202578_s_at | DDX19A | DEAD (Asp-Glu-Ala-As) box polypeptide 19A | down |
| 202740_at | ACY1 | aminoacylase 1 | down |
| 203025_at | ARD1A | ARD1 homolog A, N-acetyltransferase (S. cerevisiae) | down |
| 203146_s_at | GABBR1 | gamma-aminobutyric acid (GABA) B receptor, 1 | down |
| 203423_at | RBP1 | retinol binding protein 1, cellular | down |
| 203615_x_at | SULT1A1 | sulfotransferase family, cytosolic, 1A, phenol-preferring, member 1 | down |
| 203668_at | MAN2C1 | mannosidase, alpha, class 2C, member 1 | down |
| 203790_s_at | HRSP12 | heat-responsive protein 12 | down |
| 203802_x_at | NSUN5 | NOL1/NOP2/Sun domain family, member 5 | down |
| 204090_at | STK19 | serine/threonine kinase 19 | down |
| 204257_at | FADS3 | fatty acid desaturase 3 | down |
| 204538_x_at | LOC339047; LOC642778; LOC642799; NPIP | nuclear pore complex interacting protein; hypothetical protein LOC339047; similar to nuclear pore complex interacting protein | down |
| 204650_s_at | APBB3 | amyloid beta (A4) precursor protein-binding, family B, member 3 | down |
| 204864_s_at | IL6ST | interleukin 6 signal transducer (gp130, oncostatin M receptor) | down |
| 205130_at | RAGE | renal tumor antigen | down |
| 205344_at | CSPG5 | chondroitin sulfate proteoglycan 5 (neuroglycan C) | down |
| 205662_at | EPPB9 | B9 protein | down |
| 206527_at | ABAT | 4-aminobutyrate aminotransferase | down |
| 206541_at | KLKB1 | kallikrein B, plasma (Fletcher factor) 1 | down |
| 206582_s_at | GPR56 | G protein-coupled receptor 56 | down |
| 206701_x_at | EDNRB | endothelin receptor type B | down |
| 206846_s_at | HDAC6 | histone deacetylase 6 | down |
| 206950_at | SCN9A | sodium channel, voltage-gated, type IX, alpha subunit | down |
| 207048_at | SLC6A11 | solute carrier family 6 (neurotransmitter transporter, GABA), member 11 | down |
| 208591_s_at | PDE3B | phosphodiesterase 3B, cGMP-inhibited | down |
| 208633_s_at | MACF1 | microtubule-actin crosslinking factor 1 | down |
| 208634_s_at | MACF1 | microtubule-actin crosslinking factor 1 | down |
| 208686_s_at | BRD2 | bromodomain containing 2 | down |
| 208853_s_at | CANX | calnexin | down |
| 208922_s_at | NXF1 | nuclear RNA export factor 1 | down |
| 209091_s_at | SH3GLB1 | SH3-domain GRB2-like endophilin B1 | down |
| 209135_at | ASPH | aspartate beta-hydroxylase | down |
| 209209_s_at | PLEKHC1 | pleckstrin homology domain containing, family C (with FERM domain) member 1 | down |
| 209393_s_at | EIF4E2 | eukaryotic translation initiation factor 4E family member 2 | down |
| 209403_at | LOC653380; LOC653498; LOC727735; LOC729837; LOC729873; LOC729877; TBC1D3; TBC1D3C | TBC1 domain family, member 3; TBC1 domain family, member 3C; similar to USP6 N-terminal like; similar to TBC1 domain family member 3 (Rab GTPase-activating protein PRC17) (Prostate cancer gene 17 protein) (TRE17 alpha protein); similar to TBC1 domain family, member 3 | down |
| 209428_s_at | ZFPL1 | zinc finger protein-like 1 | down |
| 209437_s_at | SPON1 | spondin 1, extracellular matrix protein | down |
| 209450_at | OSGEP | O-sialoglycoprotein endopeptidase | down |
| 209593_s_at | TOR1B | torsin family 1, member B (torsin B) | down |
| 209623_at | MCCC2 | methylcrotonoyl-Coenzyme A carboxylase 2 (beta) | down |
| 209708_at | MOXD1 | monooxygenase, DBH-like 1 | down |
| 209940_at | PARP3 | poly (ADP-ribose) polymerase family, member 3 | down |
| 209954_x_at | SS18 | synovial sarcoma translocation, chromosome 18 | down |
| 210101_x_at | SH3GLB1 | SH3-domain GRB2-like endophilin B1 | down |
| 210153_s_at | ME2 | malic enzyme 2, NAD(+)-dependent, mitochondrial | down |
| 210738_s_at | SLC4A4 | solute carrier family 4, sodium bicarbonate cotransporter, member 4 | down |
| 210775_x_at | CASP9 | caspase 9, apoptosis-related cysteine peptidase | down |
| 210794_s_at | MEG3 | maternally expressed 3 | down |
| 210843_s_at | MFAP3L | microfibrillar-associated protein 3-like | down |
| 210896_s_at | ASPH | aspartate beta-hydroxylase | down |
| 211207_s_at | ACSL6 | acyl-CoA synthetase long-chain family member 6 | down |
| 211276_at | TCEAL2 | transcription elongation factor A (SII)-like 2 | down |
| 211569_s_at | HADH | hydroxyacyl-Coenzyme A dehydrogenase | down |
| 211574_s_at | CD46 | CD46 molecule, complement regulatory protein | down |
| 211715_s_at | BDH1 | 3-hydroxybutyrate dehydrogenase, type 1 | down |
| 211876_x_at | PCDHGA10; PCDHGA11; PCDHGA12; PCDHGA3; PCDHGA5; PCDHGA6 | protocadherin gamma subfamily A, 12; protocadherin gamma subfamily A, 11; protocadherin gamma subfamily A, 10; protocadherin gamma subfamily A, 6; protocadherin gamma subfamily A, 5; protocadherin gamma subfamily A, 3 | down |
| 211890_x_at | CAPN3 | calpain 3, (p94) | down |
| 212087_s_at | ERAL1 | Era G-protein-like 1 (E. coli) | down |
| 212228_s_at | COQ9 | coenzyme Q9 homolog (S. cerevisiae) | down |
| 212601_at | ZZEF1 | zinc finger, ZZ-type with EF-hand domain 1 | down |
| 213143_at | LOC257407 | hypothetical protein LOC257407 | down |
| 213652_at | PCSK5 | Proprotein convertase subtilisin/kexin type 5 | down |
| 213872_at | C6orf62 | Chromosome 6 open reading frame 62 | down |
| 214035_x_at | LOC399491 | LOC399491 protein | down |
| 214121_x_at | PDLIM7 | PDZ and LIM domain 7 (enigma) | down |
| 214205_x_at | TXNL2 | thioredoxin-like 2 | down |
| 214241_at | NDUFB8 | NADH dehydrogenase (ubiquinone) 1 beta subcomplex, 8, 19kDa | down |
| 214564_s_at | PCDHGC3 | protocadherin gamma subfamily C, 3 | down |
| 214882_s_at | SFRS2 | splicing factor, arginine/serine-rich 2 | down |
| 214929_s_at | KIAA1109 | KIAA1109 | down |
| 215253_s_at | DSCR1 | Down syndrome critical region gene 1 | down |
| 215683_at | --- | Clone 24803 mRNA sequence | down |
| 215794_x_at | GLUD2 | glutamate dehydrogenase 2 | down |
| 215982_s_at | DOM3Z | dom-3 homolog Z (C. elegans) | down |
| 216080_s_at | FADS3 | fatty acid desaturase 3 | down |
| 216294_s_at | KIAA1109 | KIAA1109 | down |
| 216352_x_at | PCDHGA3 | protocadherin gamma subfamily A, 3 | down |
| 216532_x_at | LOC643450; LOC728344 | similar to Thioredoxin-like protein 2 (PKC-interacting cousin of thioredoxin) (PKC-theta-interacting protein) (PKCq-interacting protein) | down |
| 216958_s_at | IVD | isovaleryl Coenzyme A dehydrogenase | down |
| 217541_x_at | LOC731901; ZNF816A | zinc finger protein 816A; similar to zinc finger protein 160 | down |
| 217889_s_at | CYBRD1 | cytochrome b reductase 1 | down |
| 218059_at | ZNF706 | zinc finger protein 706 | down |
| 218253_s_at | LGTN | ligatin | down |
| 218358_at | CRELD2 | cysteine-rich with EGF-like domains 2 | down |
| 218429_s_at | FLJ11286 | hypothetical protein FLJ11286 | down |
| 218505_at | WDR59 | WD repeat domain 59 | down |
| 218803_at | CHFR | checkpoint with forkhead and ring finger domains | down |
| 218958_at | C19orf60 | chromosome 19 open reading frame 60 | down |
| 219196_at | SCG3 | secretogranin III | down |
| 219527_at | MOSC2 | MOCO sulphurase C-terminal domain containing 2 | down |
| 219577_s_at | ABCA7 | ATP-binding cassette, sub-family A (ABC1), member 7 | down |
| 219627_at | ZNF767 | zinc finger family member 767 | down |
| 219774_at | CCDC93 | coiled-coil domain containing 93 | down |
| 219807_x_at | RAB4B | RAB4B, member RAS oncogene family | down |
| 220029_at | ELOVL2 | elongation of very long chain fatty acids (FEN1/Elo2, SUR4/Elo3, yeast)-like 2 | down |
| 220642_x_at | GPR89A; LOC728932; UNQ192 | G protein-coupled receptor 89A; similar to G protein-coupled receptor 89 | down |
| 220892_s_at | PSAT1 | phosphoserine aminotransferase 1 | down |
| 221036_s_at | APH1B | anterior pharynx defective 1 homolog B (C. elegans) | down |
| 221050_s_at | GTPBP2 | GTP binding protein 2 | down |
| 221307_at | KCNIP1 | Kv channel interacting protein 1 | down |
| 221501_x_at | LOC339047 | hypothetical protein LOC339047 | down |
| 221535_at | LSG1 | large subunit GTPase 1 homolog (S. cerevisiae) | down |
| 221636_s_at | MOSC2 | MOCO sulphurase C-terminal domain containing 2 | down |
| 221739_at | C19orf10 | chromosome 19 open reading frame 10 | down |
| 221881_s_at | CLIC4 | chloride intracellular channel 4 | down |
| 221972_s_at | SDF4 | stromal cell derived factor 4 | down |
| 222026_at | RBM3 | RNA binding motif (RNP1, RRM) protein 3 | down |
| 222150_s_at | LOC54103 | hypothetical protein LOC54103 | down |
| 222302_at | --- | --- | down |
| 222385_x_at | SEC61A1 | Sec61 alpha 1 subunit (S. cerevisiae) | down |
| 222780_s_at | BAALC | brain and acute leukemia, cytoplasmic | down |
| 223390_at | C9orf37 | chromosome 9 open reading frame 37 | down |
| 223528_s_at | LOC731602; METT11D1 | methyltransferase 11 domain containing 1; similar to methyltransferase 11 domain containing 1 isoform 2 | down |
| 223534_s_at | RPS6KL1 | ribosomal protein S6 kinase-like 1 | down |
| 223539_s_at | LOC728492; SERF1A; SERF1B | small EDRK-rich factor 1A (telomeric); small EDRK-rich factor 1B (centromeric); similar to small EDRK-rich factor 1A, telomeric | down |
| 224196_x_at | DPH5 | DPH5 homolog (S. cerevisiae) | down |
| 224727_at | LOC284361 | hematopoietic signal peptide-containing | down |
| 224804_s_at | C15orf17 | chromosome 15 open reading frame 17 | down |
| 224904_at | PDPR | pyruvate dehydrogenase phosphatase regulatory subunit | down |
| 225035_x_at | CXYorf1; FAM39B; FAM39DP; FLJ00038; LOC376475; LOC653635 | family with sequence similarity 39, member D pseudogene; family with sequence similarity 39, member B; CXYorf1-related protein; chromosomes X and Y open reading frame 1; similar to CXYorf1-related protein | down |
| 225298_at | PNKD | paroxysmal nonkinesiogenic dyskinesia | down |
| 225311_at | IVD | isovaleryl Coenzyme A dehydrogenase | down |
| 225529_at | CENTB5 | centaurin, beta 5 | down |
| 225758_s_at | TUBGCP6 | tubulin, gamma complex associated protein 6 | down |
| 225866_at | BXDC1 | brix domain containing 1 | down |
| 225973_at | TAP2 | transporter 2, ATP-binding cassette, sub-family B (MDR/TAP) | down |
| 225995_x_at | FAM39B | family with sequence similarity 39, member B | down |
| 226179_at | SLC25A37 | solute carrier family 25, member 37 | down |
| 226354_at | LACTB | lactamase, beta | down |
| 226620_x_at | DAZAP1 | DAZ associated protein 1 | down |
| 226848_at | --- | CDNA FLJ39306 fis, clone OCBBF2013123 | down |
| 227168_at | MIAT | myocardial infarction associated transcript (non-protein coding) | down |
| 227208_at | CCDC84 | coiled-coil domain containing 84 | down |
| 227286_at | CCDC95 | coiled-coil domain containing 95 | down |
| 227347_x_at | HES4 | hairy and enhancer of split 4 (Drosophila) | down |
| 227417_at | MOSC2 | MOCO sulphurase C-terminal domain containing 2 | down |
| 227431_at | --- | CDNA clone IMAGE:4791585 | down |
| 227640_s_at | LOC441212; RP9 | retinitis pigmentosa 9 (autosomal dominant); retinitis pigmentosa 9 pseudogene | down |
| 227677_at | JAK3 | Janus kinase 3 (a protein tyrosine kinase, leukocyte) | down |
| 229351_at | --- | CDNA FLJ13620 fis, clone PLACE1010947 | down |
| 229654_at | ZNF44 | Zinc finger protein 44 | down |
| 230941_at | LOC728537; LOC730797 | hypothetical protein LOC728537; hypothetical protein LOC730797 | down |
| 231116_at | --- | Transcribed locus | down |
| 231164_at | LOC440331 | hypothetical gene supported by AK095200; BC042853 | down |
| 231240_at | DIO2 | deiodinase, iodothyronine, type II | down |
| 231828_at | LOC253039 | Hypothetical protein LOC253039 | down |
| 232392_at | SFRS3 | Splicing factor, arginine/serine-rich 3 | down |
| 232408_at | ZFYVE28 | zinc finger, FYVE domain containing 28 | down |
| 232663_s_at | LOC390595 | similar to ubiquitin-associated protein 1 (predicted) | down |
| 233168_s_at | RP3-402G11.5 | selenoprotein O | down |
| 233851_s_at | TOR3A | torsin family 3, member A | down |
| 235902_at | --- | CDNA FLJ42963 fis, clone BRSTN2012380 | down |
| 237108_x_at | FLJ42875 | FLJ42875 protein | down |
| 238743_at | --- | Full-length cDNA clone CS0DK002YF13 of HeLa cells Cot 25-normalized of Homo sapiens (human) | down |
| 239190_at | VRK3 | vaccinia related kinase 3 | down |
| 239856_at | --- | Transcribed locus, strongly similar to XP_001175123.1 hypothetical protein [Pan troglodytes] | down |
| 244241_x_at | --- | Transcribed locus | down |
| 33132_at | CPSF1 | cleavage and polyadenylation specific factor 1, 160kDa | down |
| 36019_at | STK19 | serine/threonine kinase 19 | down |
| 36936_at | TSTA3 | tissue specific transplantation antigen P35B | down |
| 37278_at | TAZ | tafazzin (cardiomyopathy, dilated 3A (X-linked); endocardial fibroelastosis 2; Barth syndrome) | down |
| 38157_at | DOM3Z | dom-3 homolog Z (C. elegans) | down |
| 39854_r_at | PNPLA2 | patatin-like phospholipase domain containing 2 | down |
| 46167_at | C1orf175; TTC4 | tetratricopeptide repeat domain 4; chromosome 1 open reading frame 175 | down |
| 90610_at | LRCH4 | leucine-rich repeats and calponin homology (CH) domain containing 4 | down |
| 1552536_at | VTI1A | vesicle transport through interaction with t-SNAREs homolog 1A (yeast) | up |
| 1553407_at | MACF1 | microtubule-actin crosslinking factor 1 | up |
| 1554963_at | --- | CDNA clone IMAGE:5310797 | up |
| 1555318_at | HIF3A | hypoxia inducible factor 3, alpha subunit | up |
| 1556000_s_at | BTBD7 | BTB (POZ) domain containing 7 | up |
| 1556606_at | NAV2 | neuron navigator 2 | up |
| 1557803_at | --- | Full length insert cDNA clone YZ56G10 | up |
| 1559618_at | --- | CDNA FLJ30384 fis, clone BRACE2008114 | up |
| 1561167_at | --- | Full length insert cDNA clone YA75A09 | up |
| 1561195_at | --- | MRNA; cDNA DKFZp686A22111 (from clone DKFZp686A22111) | up |
| 1561362_at | --- | CDNA FLJ36285 fis, clone THYMU2003470 | up |
| 1565692_at | --- | CDNA FLJ40647 fis, clone THYMU2017522 | up |
| 1566551_at | --- | MRNA; cDNA DKFZp586L2217 (from clone DKFZp586L2217) | up |
| 1566887_x_at | --- | Multiple myeloma susceptibility mRNA sequence | up |
| 1568619_s_at | LOC162073 | hypothetical protein LOC162073 | up |
| 1569477_at | --- | Homo sapiens, clone IMAGE:4291396, mRNA | up |
| 1570511_at | ARHGEF10L | Rho guanine nucleotide exchange factor (GEF) 10-like | up |
| 202040_s_at | JARID1A | jumonji, AT rich interactive domain 1A | up |
| 202935_s_at | SOX9 | SRY (sex determining region Y)-box 9 (campomelic dysplasia, autosomal sex-reversal) | up |
| 205435_s_at | AAK1 | AP2 associated kinase 1 | up |
| 206548_at | FLJ23556 | hypothetical protein FLJ23556 | up |
| 207660_at | DMD | dystrophin (muscular dystrophy, Duchenne and Becker types) | up |
| 210407_at | PPM1A | protein phosphatase 1A (formerly 2C), magnesium-dependent, alpha isoform | up |
| 212079_s_at | MLL | myeloid/lymphoid or mixed-lineage leukemia (trithorax homolog, Drosophila) | up |
| 215600_x_at | FBXW12 | F-box and WD repeat domain containing 12 | up |
| 216189_at | --- | Homo sapiens, clone IMAGE:3344506 | up |
| 219975_x_at | OLAH | oleoyl-ACP hydrolase | up |
| 220694_at | DDEF1IT1 | DDEF1 intronic transcript 1 | up |
| 221191_at | DKFZP434A0131 | DKFZp434A0131 protein | up |
| 221829_s_at | TNPO1 | transportin 1 | up |
| 222282_at | --- | Transcribed locus | up |
| 222366_at | --- | Transcribed locus | up |
| 224771_at | NAV1 | neuron navigator 1 | up |
| 225234_at | CBL | Cas-Br-M (murine) ecotropic retroviral transforming sequence | up |
| 227931_at | --- | MRNA; cDNA DKFZp686D22106 (from clone DKFZp686D22106) | up |
| 229467_at | PCBP2 | Poly(rC) binding protein 2 | up |
| 231109_at | --- | CDNA FLJ38468 fis, clone FEBRA2021864 | up |
| 231495_at | --- | Transcribed locus | up |
| 232541_at | --- | CDNA FLJ20099 fis, clone COL04544 | up |
| 232685_at | --- | CDNA: FLJ21564 fis, clone COL06452 | up |
| 232882_at | --- | CDNA FLJ12289 fis, clone MAMMA1001788 | up |
| 232935_at | --- | Primary neuroblastoma cDNA, clone:Nbla03614, full insert sequence | up |
| 233449_at | --- | CDNA FLJ11377 fis, clone HEMBA1000442 | up |
| 235084_x_at | --- | Transcribed locus | up |
| 235508_at | PML | promyelocytic leukemia | up |
| 235538_at | --- | CDNA FLJ30718 fis, clone FCBBF2001675 | up |
| 235756_at | --- | CDNA FLJ26187 fis, clone ADG04782 | up |
| 236617_at | --- | Transcribed locus | up |
| 236946_at | GPR75 | G protein-coupled receptor 75 | up |
| 238884_at | --- | Transcribed locus | up |
| 239167_at | --- | Transcribed locus | up |
| 239661_at | --- | Transcribed locus | up |
| 240125_at | --- | Transcribed locus | up |
| 240665_at | --- | --- | up |
| 240971_x_at | --- | --- | up |
| 241797_at | --- | --- | up |
| 242106_at | --- | Transcribed locus | up |
| 242171_at | --- | --- | up |
| 242303_at | --- | Transcribed locus | up |
| 242320_at | --- | Homo sapiens, clone IMAGE:4769230, mRNA | up |
| 242736_at | --- | --- | up |
| 243158_at | --- | --- | up |
| 243291_at | --- | Transcribed locus | up |
| 243431_at | --- | Transcribed locus | up |
| 244345_at | CADM1 | cell adhesion molecule 1 | up |
| 244358_at | --- | --- | up |
| 244535_at | --- | Transcribed locus | up |
| 244669_at | SNHG5; SNORD50A; SNORD50B | small nucleolar RNA, C/D box 50A; small nucleolar RNA host gene (non-protein coding) 5; small nucleolar RNA, C/D box 50B | up |
| 244697_at | --- | --- | up |
|  |  |  |  |
| **Pick's disease** | | | |
| Probe Set ID | Gene Symbol | Gene Title | Expression |
| 1552301_a_at | CORO6 | coronin 6 | down |
| 1553565_s_at | DDAH1 | dimethylarginine dimethylaminohydrolase 1 | down |
| 1553995_a_at | NT5E | 5'-nucleotidase, ecto (CD73) | down |
| 1555470_a_at | PPM1F | protein phosphatase 1F (PP2C domain containing) | down |
| 1557186_s_at | TPCN1 | two pore segment channel 1 | down |
| 1558009_at | SLC1A2 | solute carrier family 1 (glial high affinity glutamate transporter), member 2 | down |
| 1558010_s_at | SLC1A2 | solute carrier family 1 (glial high affinity glutamate transporter), member 2 | down |
| 1558041_a_at | LOC653319 | hypothetical protein LOC653319 | down |
| 1559965_at | --- | CDNA clone IMAGE:4811567 | down |
| 200644_at | MARCKSL1 | MARCKS-like 1 | down |
| 200778_s_at | SEPT2 | septin 2 | down |
| 201135_at | ECHS1 | enoyl Coenzyme A hydratase, short chain, 1, mitochondrial | down |
| 201185_at | HTRA1 | HtrA serine peptidase 1 | down |
| 203179_at | GALT | galactose-1-phosphate uridylyltransferase | down |
| 203229_s_at | CLK2 | CDC-like kinase 2 | down |
| 203452_at | B3GAT3 | beta-1,3-glucuronyltransferase 3 (glucuronosyltransferase I) | down |
| 203668_at | MAN2C1 | mannosidase, alpha, class 2C, member 1 | down |
| 203802_x_at | NSUN5 | NOL1/NOP2/Sun domain family, member 5 | down |
| 204193_at | CHKB; CPT1B | choline kinase beta; carnitine palmitoyltransferase 1B (muscle) | down |
| 204257_at | FADS3 | fatty acid desaturase 3 | down |
| 204538_x_at | LOC339047; LOC642778; LOC642799; NPIP | nuclear pore complex interacting protein; hypothetical protein LOC339047; similar to nuclear pore complex interacting protein | down |
| 204650_s_at | APBB3 | amyloid beta (A4) precursor protein-binding, family B, member 3 | down |
| 205130_at | RAGE | renal tumor antigen | down |
| 205318_at | KIF5A | kinesin family member 5A | down |
| 205344_at | CSPG5 | chondroitin sulfate proteoglycan 5 (neuroglycan C) | down |
| 205839_s_at | BZRAP1 | benzodiazapine receptor (peripheral) associated protein 1 | down |
| 206527_at | ABAT | 4-aminobutyrate aminotransferase | down |
| 206950_at | SCN9A | sodium channel, voltage-gated, type IX, alpha subunit | down |
| 207122_x_at | SULT1A2 | sulfotransferase family, cytosolic, 1A, phenol-preferring, member 2 | down |
| 208686_s_at | BRD2 | bromodomain containing 2 | down |
| 208829_at | TAPBP | TAP binding protein (tapasin) | down |
| 208871_at | ATN1 | atrophin 1 | down |
| 211876_x_at | PCDHGA10; PCDHGA11; PCDHGA12; PCDHGA3; PCDHGA5; PCDHGA6 | protocadherin gamma subfamily A, 12; protocadherin gamma subfamily A, 11; protocadherin gamma subfamily A, 10; protocadherin gamma subfamily A, 6; protocadherin gamma subfamily A, 5; protocadherin gamma subfamily A, 3 | down |
| 212059_s_at | TRPC4AP | transient receptor potential cation channel, subfamily C, member 4 associated protein | down |
| 212228_s_at | COQ9 | coenzyme Q9 homolog (S. cerevisiae) | down |
| 213143_at | LOC257407 | hypothetical protein LOC257407 | down |
| 213652_at | PCSK5 | Proprotein convertase subtilisin/kexin type 5 | down |
| 214427_at | NOL1 | nucleolar protein 1, 120kDa | down |
| 214882_s_at | SFRS2 | splicing factor, arginine/serine-rich 2 | down |
| 215683_at | --- | Clone 24803 mRNA sequence | down |
| 215836_s_at | PCDHGA1; PCDHGA10; PCDHGA11; PCDHGA12; PCDHGA2; PCDHGA3; PCDHGA4; PCDHGA5; PCDHGA6; PCDHGA7; PCDHGA8; PCDHGA9; PCDHGB1; PCDHGB2; PCDHGB3; PCDHGB4; PCDHGB5; PCDHGB6; PCDHGB7; PCDHGC3; PCDHGC4; PCDHGC5 | protocadherin gamma subfamily C, 3; protocadherin gamma subfamily B, 4; protocadherin gamma subfamily A, 8; protocadherin gamma subfamily A, 12; protocadherin gamma subfamily C, 5; protocadherin gamma subfamily C, 4; protocadherin gamma subfamily B, 7; protocadherin gamma subfamily B, 6; protocadherin gamma subfamily B, 5; protocadherin gamma subfamily B, 3; protocadherin gamma subfamily B, 2; protocadherin gamma subfamily B, 1; protocadherin gamma subfamily A, 11; protocadherin gamma subfamily A, 10; protocadherin gamma subfamily A, 9; protocadherin gamma subfamily A, 7; protocadherin gamma subfamily A, 6; protocadherin gamma subfamily A, 5; protocadherin gamma subfamily A, 4; protocadherin gamma subfamily A, 3; protocadherin gamma subfamily A, 2; protocadherin gamma subfamily A, 1 | down |
| 215982_s_at | DOM3Z | dom-3 homolog Z (C. elegans) | down |
| 216532_x_at | LOC643450; LOC728344 | similar to Thioredoxin-like protein 2 (PKC-interacting cousin of thioredoxin) (PKC-theta-interacting protein) (PKCq-interacting protein) | down |
| 216958_s_at | IVD | isovaleryl Coenzyme A dehydrogenase | down |
| 218803_at | CHFR | checkpoint with forkhead and ring finger domains | down |
| 218958_at | C19orf60 | chromosome 19 open reading frame 60 | down |
| 219333_s_at | CAPN10 | calpain 10 | down |
| 221307_at | KCNIP1 | Kv channel interacting protein 1 | down |
| 221501_x_at | LOC339047 | hypothetical protein LOC339047 | down |
| 221636_s_at | MOSC2 | MOCO sulphurase C-terminal domain containing 2 | down |
| 222026_at | RBM3 | RNA binding motif (RNP1, RRM) protein 3 | down |
| 223528_s_at | LOC731602; METT11D1 | methyltransferase 11 domain containing 1; similar to methyltransferase 11 domain containing 1 isoform 2 | down |
| 223534_s_at | RPS6KL1 | ribosomal protein S6 kinase-like 1 | down |
| 223933_at | KIF5A | kinesin family member 5A | down |
| 225035_x_at | CXYorf1; FAM39B; FAM39DP; FLJ00038; LOC376475; LOC653635 | family with sequence similarity 39, member D pseudogene; family with sequence similarity 39, member B; CXYorf1-related protein; chromosomes X and Y open reading frame 1; similar to CXYorf1-related protein | down |
| 225311_at | IVD | isovaleryl Coenzyme A dehydrogenase | down |
| 225529_at | CENTB5 | centaurin, beta 5 | down |
| 225995_x_at | FAM39B | family with sequence similarity 39, member B | down |
| 226200_at | VARS2 | valyl-tRNA synthetase 2, mitochondrial (putative) | down |
| 226620_x_at | DAZAP1 | DAZ associated protein 1 | down |
| 228155_at | C10orf58 | chromosome 10 open reading frame 58 | down |
| 232663_s_at | LOC390595 | similar to ubiquitin-associated protein 1 (predicted) | down |
| 237108_x_at | FLJ42875 | FLJ42875 protein | down |
| 238743_at | --- | Full-length cDNA clone CS0DK002YF13 of HeLa cells Cot 25-normalized of Homo sapiens (human) | down |
| 242670_at | LGI4 | leucine-rich repeat LGI family, member 4 | down |
| 36936_at | TSTA3 | tissue specific transplantation antigen P35B | down |
| 37278_at | TAZ | tafazzin (cardiomyopathy, dilated 3A (X-linked); endocardial fibroelastosis 2; Barth syndrome) | down |
| 52078_at | C1orf160 | chromosome 1 open reading frame 160 | down |
| 1552536_at | VTI1A | vesicle transport through interaction with t-SNAREs homolog 1A (yeast) | up |
| 1554473_at | SRGAP1 | SLIT-ROBO Rho GTPase activating protein 1 | up |
| 1554703_at | ARHGEF10 | Rho guanine nucleotide exchange factor (GEF) 10 | up |
| 1555014_x_at | --- | OK/SW-cl.92 | up |
| 1555259_at | ZAK | sterile alpha motif and leucine zipper containing kinase AZK | up |
| 1555372_at | BCL2L11 | BCL2-like 11 (apoptosis facilitator) | up |
| 1556000_s_at | BTBD7 | BTB (POZ) domain containing 7 | up |
| 1556352_at | --- | CDNA FLJ30440 fis, clone BRACE2009185 | up |
| 1556606_at | NAV2 | neuron navigator 2 | up |
| 1556650_at | --- | CDNA FLJ13011 fis, clone NT2RP3000561 | up |
| 1556658_a_at | --- | CDNA FLJ36459 fis, clone THYMU2014762 | up |
| 1556818_at | --- | Full length insert cDNA clone YB35F05 | up |
| 1556849_at | --- | CDNA FLJ11909 fis, clone HEMBB1000099 | up |
| 1557505_a_at | --- | Full length insert cDNA YQ11E04 | up |
| 1557690_x_at | --- | CDNA FLJ11951 fis, clone HEMBB1000827 | up |
| 1557706_at | ZHX2 | zinc fingers and homeoboxes 2 | up |
| 1557889_at | --- | CDNA clone IMAGE:4138742 | up |
| 1558678_s_at | MALAT1 | metastasis associated lung adenocarcinoma transcript 1 (non-coding RNA) | up |
| 1558783_at | --- | CDNA: FLJ21152 fis, clone CAS09594 | up |
| 1558831_x_at | --- | CDNA FLJ34403 fis, clone HCHON2001607 | up |
| 1558832_at | FLJ32224 | hypothetical gene supported by AK056786 | up |
| 1558877_at | --- | CDNA FLJ36355 fis, clone THYMU2007384 | up |
| 1559020_a_at | --- | CDNA FLJ14081 fis, clone HEMBB1002280 | up |
| 1559156_at | --- | MRNA; cDNA DKFZp686B1142 (from clone DKFZp686B1142) | up |
| 1559249_at | ATXN1 | Ataxin 1 | up |
| 1559375_s_at | --- | Full length insert cDNA clone YI45C08 | up |
| 1559436_x_at | --- | MRNA; cDNA DKFZp313M2114 (from clone DKFZp313M2114) | up |
| 1559593_a_at | CRSP7 | Cofactor required for Sp1 transcriptional activation, subunit 7, 70kDa | up |
| 1559987_at | --- | Homo sapiens, clone IMAGE:5585678, mRNA | up |
| 1560798_at | --- | CDNA FLJ14121 fis, clone MAMMA1002009 | up |
| 1560926_at | --- | Full length insert cDNA clone YR43G06 | up |
| 1561166_a_at | --- | Full length insert cDNA clone YB22D01 | up |
| 1561180_at | --- | CDNA FLJ11745 fis, clone HEMBA1005526 | up |
| 1561346_at | --- | CDNA FLJ32691 fis, clone TESTI2000221 | up |
| 1561777_at | --- | (TL22) mRNA from LNCaP cell line | up |
| 1562062_at | KIAA1245; LOC728895; NBPF1; NBPF10; NBPF11; NBPF20; NBPF3; NBPF8; NBPF9; XXyac-YX155B6.1 | neuroblastoma breakpoint family, member 1; neuroblastoma breakpoint family, member 3; KIAA1245; neuroblastoma breakpoint family, member 11; neuroblastoma breakpoint family, member 20; neuroblastoma breakpoint family, member 9; neuroblastoma breakpoint family, member 10; neuroblastoma breakpoint family, member 8; hypothetical protein LOC728895; CLIP-190-like | up |
| 1562063_x_at | KIAA1245; LOC728895; NBPF1; NBPF10; NBPF11; NBPF20; NBPF3; NBPF8; NBPF9; XXyac-YX155B6.1 | neuroblastoma breakpoint family, member 1; neuroblastoma breakpoint family, member 3; KIAA1245; neuroblastoma breakpoint family, member 11; neuroblastoma breakpoint family, member 20; neuroblastoma breakpoint family, member 9; neuroblastoma breakpoint family, member 10; neuroblastoma breakpoint family, member 8; hypothetical protein LOC728895; CLIP-190-like | up |
| 1562948_at | --- | Homo sapiens, clone IMAGE:5722724, mRNA | up |
| 1563321_s_at | MLLT10 | myeloid/lymphoid or mixed-lineage leukemia (trithorax homolog, Drosophila); translocated to, 10 | up |
| 1563482_at | --- | CDNA FLJ26750 fis, clone PRS01773 | up |
| 1565743_at | --- | CDNA FLJ37648 fis, clone BRHIP2000532 | up |
| 1566482_at | --- | MRNA; cDNA DKFZp313L2229 (from clone DKFZp313L2229) | up |
| 1566539_at | --- | MRNA; cDNA DKFZp586G081 (from clone DKFZp586G081) | up |
| 1566887_x_at | --- | Multiple myeloma susceptibility mRNA sequence | up |
| 1568866_at | --- | CDNA clone IMAGE:5450715 | up |
| 1569323_at | PTPRG | protein tyrosine phosphatase, receptor type, G | up |
| 1569477_at | --- | Homo sapiens, clone IMAGE:4291396, mRNA | up |
| 1569661_at | --- | CDNA clone IMAGE:5260324 | up |
| 1569948_at | --- | CDNA clone IMAGE:5275301 | up |
| 1570414_x_at | FLJ13197 | hypothetical FLJ13197 | up |
| 201220_x_at | CTBP2 | C-terminal binding protein 2 | up |
| 201404_x_at | PSMB2 | proteasome (prosome, macropain) subunit, beta type, 2 | up |
| 201667_at | GJA1 | gap junction protein, alpha 1, 43kDa | up |
| 201867_s_at | TBL1X | transducin (beta)-like 1X-linked | up |
| 201901_s_at | YY1 | YY1 transcription factor | up |
| 201996_s_at | SPEN | spen homolog, transcriptional regulator (Drosophila) | up |
| 202040_s_at | JARID1A | jumonji, AT rich interactive domain 1A | up |
| 202449_s_at | RXRA | retinoid X receptor, alpha | up |
| 202935_s_at | SOX9 | SRY (sex determining region Y)-box 9 (campomelic dysplasia, autosomal sex-reversal) | up |
| 202975_s_at | RHOBTB3 | Rho-related BTB domain containing 3 | up |
| 203007_x_at | LYPLA1 | lysophospholipase I | up |
| 203496_s_at | PPARBP | PPAR binding protein | up |
| 203628_at | IGF1R | insulin-like growth factor 1 receptor | up |
| 204461_x_at | RAD1 | RAD1 homolog (S. pombe) | up |
| 204786_s_at | IFNAR2 | interferon (alpha, beta and omega) receptor 2 | up |
| 205187_at | SMAD5 | SMAD family member 5 | up |
| 205255_x_at | TCF7 | transcription factor 7 (T-cell specific, HMG-box) | up |
| 205383_s_at | ZBTB20 | zinc finger and BTB domain containing 20 | up |
| 205887_x_at | MSH3 | mutS homolog 3 (E. coli) | up |
| 206056_x_at | SPN | sialophorin (leukosialin, CD43) | up |
| 206565_x_at | SMA3 | SMA3 | up |
| 206792_x_at | PDE4C | phosphodiesterase 4C, cAMP-specific (phosphodiesterase E1 dunce homolog, Drosophila) | up |
| 207657_x_at | TNPO1 | transportin 1 | up |
| 207660_at | DMD | dystrophin (muscular dystrophy, Duchenne and Becker types) | up |
| 208054_at | HERC4 | hect domain and RLD 4 | up |
| 208238_x_at | --- | --- | up |
| 209074_s_at | FAM107A | family with sequence similarity 107, member A | up |
| 209225_x_at | TNPO1 | transportin 1 | up |
| 210407_at | PPM1A | protein phosphatase 1A (formerly 2C), magnesium-dependent, alpha isoform | up |
| 210528_at | MR1 | major histocompatibility complex, class I-related | up |
| 210556_at | NFATC3 | nuclear factor of activated T-cells, cytoplasmic, calcineurin-dependent 3 | up |
| 210778_s_at | MXD4 | MAX dimerization protein 4 | up |
| 210835_s_at | CTBP2 | C-terminal binding protein 2 | up |
| 211316_x_at | CFLAR | CASP8 and FADD-like apoptosis regulator | up |
| 211713_x_at | KIAA0101 | KIAA0101 | up |
| 212079_s_at | MLL | myeloid/lymphoid or mixed-lineage leukemia (trithorax homolog, Drosophila) | up |
| 212291_at | HIPK1 | homeodomain interacting protein kinase 1 | up |
| 212492_s_at | JMJD2B | jumonji domain containing 2B | up |
| 212852_s_at | TROVE2 | TROVE domain family, member 2 | up |
| 213002_at | MARCKS | Myristoylated alanine-rich protein kinase C substrate | up |
| 213236_at | SASH1 | SAM and SH3 domain containing 1 | up |
| 213956_at | CEP350 | centrosomal protein 350kDa | up |
| 214004_s_at | VGLL4 | vestigial like 4 (Drosophila) | up |
| 214104_at | GPR161 | G protein-coupled receptor 161 | up |
| 214176_s_at | PBXIP1 | Pre-B-cell leukemia homeobox interacting protein 1 | up |
| 214329_x_at | TNFSF10 | Tumor necrosis factor (ligand) superfamily, member 10 | up |
| 214405_at | --- | Clone 23705 mRNA sequence | up |
| 214707_x_at | ALMS1 | Alstrom syndrome 1 | up |
| 214743_at | CUTL1 | cut-like 1, CCAAT displacement protein (Drosophila) | up |
| 214806_at | BICD1 | bicaudal D homolog 1 (Drosophila) | up |
| 214902_x_at | --- | MRNA; cDNA DKFZp586A061 (from clone DKFZp586A061) | up |
| 215067_x_at | PRDX2 | peroxiredoxin 2 | up |
| 215310_at | APC | Adenomatosis polyposis coli | up |
| 215350_at | SYNE1 | spectrin repeat containing, nuclear envelope 1 | up |
| 215372_x_at | --- | CDNA FLJ12002 fis, clone HEMBB1001536 | up |
| 215383_x_at | SPG21 | spastic paraplegia 21 (autosomal recessive, Mast syndrome) | up |
| 215385_at | --- | CDNA FLJ12411 fis, clone MAMMA1002964 | up |
| 215390_at | --- | CDNA FLJ12102 fis, clone HEMBB1002684 | up |
| 215435_at | --- | CDNA FLJ11921 fis, clone HEMBB1000318 | up |
| 215439_x_at | --- | CDNA FLJ11924 fis, clone HEMBB1000343 | up |
| 215507_x_at | --- | Transcribed locus | up |
| 215588_x_at | RIOK3 | RIO kinase 3 (yeast) | up |
| 215589_at | --- | CDNA: FLJ21284 fis, clone COL01911 | up |
| 215595_x_at | --- | CDNA FLJ13856 fis, clone THYRO1000988 | up |
| 215600_x_at | FBXW12 | F-box and WD repeat domain containing 12 | up |
| 215615_x_at | --- | CDNA FLJ14152 fis, clone MAMMA1003089 | up |
| 215653_at | --- | Clone IMAGE:248602, mRNA sequence | up |
| 215698_at | JARID1A | jumonji, AT rich interactive domain 1A | up |
| 216101_at | --- | Full length insert cDNA clone YR67C11 | up |
| 216123_x_at | --- | CDNA FLJ14096 fis, clone MAMMA1000752 | up |
| 216187_x_at | --- | Alu repeat (LNX1) mRNA sequence | up |
| 216189_at | --- | Homo sapiens, clone IMAGE:3344506 | up |
| 217536_x_at | --- | Transcribed locus | up |
| 217550_at | ATF6 | Activating transcription factor 6 | up |
| 217643_x_at | --- | --- | up |
| 217662_x_at | --- | Transcribed locus | up |
| 217679_x_at | --- | --- | up |
| 217713_x_at | --- | --- | up |
| 217715_x_at | --- | --- | up |
| 217810_x_at | LARS | leucyl-tRNA synthetase | up |
| 219392_x_at | PRR11 | proline rich 11 | up |
| 219426_at | EIF2C3 | eukaryotic translation initiation factor 2C, 3 | up |
| 219975_x_at | OLAH | oleoyl-ACP hydrolase | up |
| 220071_x_at | CEP27 | centrosomal protein 27kDa | up |
| 220113_x_at | POLR1B | polymerase (RNA) I polypeptide B, 128kDa | up |
| 220694_at | DDEF1IT1 | DDEF1 intronic transcript 1 | up |
| 220791_x_at | SCN11A | sodium channel, voltage-gated, type XI, alpha subunit | up |
| 221176_x_at | WBSCR23 | Williams-Beuren syndrome chromosome region 23 | up |
| 221829_s_at | TNPO1 | transportin 1 | up |
| 221963_x_at | --- | Transcribed locus | up |
| 222024_s_at | AKAP13 | A kinase (PRKA) anchor protein 13 | up |
| 222104_x_at | GTF2H3 | general transcription factor IIH, polypeptide 3, 34kDa | up |
| 222158_s_at | C1orf121 | chromosome 1 open reading frame 121 | up |
| 222284_at | --- | Transcribed locus | up |
| 222319_at | --- | --- | up |
| 222320_at | --- | Transcribed locus | up |
| 222366_at | --- | Transcribed locus | up |
| 222540_s_at | RSF1 | remodeling and spacing factor 1 | up |
| 222762_x_at | LIMD1 | LIM domains containing 1 | up |
| 223134_at | BBX | bobby sox homolog (Drosophila) | up |
| 223519_at | ZAK | sterile alpha motif and leucine zipper containing kinase AZK | up |
| 224105_x_at | --- | Clone FLB8034 PRO2158 | up |
| 224567_x_at | MALAT1 | metastasis associated lung adenocarcinoma transcript 1 (non-coding RNA) | up |
| 224569_s_at | IRF2BP2 | interferon regulatory factor 2 binding protein 2 | up |
| 224667_x_at | C10orf104 | chromosome 10 open reading frame 104 | up |
| 224712_x_at | C19orf42 | chromosome 19 open reading frame 42 | up |
| 224771_at | NAV1 | neuron navigator 1 | up |
| 224970_at | NFIA | nuclear factor I/A | up |
| 225234_at | CBL | Cas-Br-M (murine) ecotropic retroviral transforming sequence | up |
| 225269_s_at | RBMS1 | RNA binding motif, single stranded interacting protein 1 | up |
| 226153_s_at | CNOT6L | CCR4-NOT transcription complex, subunit 6-like | up |
| 226252_at | --- | CDNA FLJ34585 fis, clone KIDNE2008758 | up |
| 226876_at | FAM101B | family with sequence similarity 101, member B | up |
| 227039_at | AKAP13 | A kinase (PRKA) anchor protein 13 | up |
| 227082_at | --- | MRNA; cDNA DKFZp586K1922 (from clone DKFZp586K1922) | up |
| 227484_at | --- | CDNA FLJ41690 fis, clone HCASM2009405 | up |
| 227948_at | FGD4 | FYVE, RhoGEF and PH domain containing 4 | up |
| 229272_at | FNBP4 | formin binding protein 4 | up |
| 229467_at | PCBP2 | Poly(rC) binding protein 2 | up |
| 229574_at | TRA2A | transformer-2 alpha | up |
| 229686_at | P2RY8 | purinergic receptor P2Y, G-protein coupled, 8 | up |
| 229851_s_at | C11orf54 | chromosome 11 open reading frame 54 | up |
| 229858_at | --- | CDNA FLJ12024 fis, clone HEMBB1001797 | up |
| 229943_at | TRIM13 | tripartite motif-containing 13 | up |
| 230014_at | --- | Transcribed locus | up |
| 230200_at | NSUN6 | NOL1/NOP2/Sun domain family, member 6 | up |
| 230599_at | --- | Transcribed locus | up |
| 230630_at | --- | Transcribed locus | up |
| 230733_at | --- | Transcribed locus | up |
| 230820_at | --- | Transcribed locus | up |
| 231109_at | --- | CDNA FLJ38468 fis, clone FEBRA2021864 | up |
| 231212_x_at | --- | --- | up |
| 231238_at | --- | Transcribed locus | up |
| 231696_x_at | --- | Transcribed locus | up |
| 231829_at | VISA | virus-induced signaling adapter | up |
| 232096_x_at | --- | CDNA: FLJ22140 fis, clone HEP20977 | up |
| 232125_at | --- | CDNA FLJ34585 fis, clone KIDNE2008758 | up |
| 232175_at | ARF1 | ADP-ribosylation factor 1 | up |
| 232215_x_at | PRR11 | proline rich 11 | up |
| 232216_at | YME1L1 | YME1-like 1 (S. cerevisiae) | up |
| 232264_at | --- | CDNA FLJ12142 fis, clone MAMMA1000356 | up |
| 232347_x_at | --- | CDNA FLJ11379 fis, clone HEMBA1000469 | up |
| 232420_x_at | LOC286260 | hypothetical protein LOC286260 | up |
| 232472_at | --- | CDNA FLJ12399 fis, clone MAMMA1002780 | up |
| 232516_x_at | YY1AP1 | YY1 associated protein 1 | up |
| 232537_x_at | MARK3 | MAP/microtubule affinity-regulating kinase 3 | up |
| 232541_at | --- | CDNA FLJ20099 fis, clone COL04544 | up |
| 232614_at | --- | CDNA FLJ12049 fis, clone HEMBB1001996 | up |
| 232615_at | --- | CDNA: FLJ22765 fis, clone KAIA1180 | up |
| 232665_x_at | --- | --- | up |
| 232691_at | SFXN5 | Sideroflexin 5 | up |
| 232693_s_at | FBXO16; ZNF395 | zinc finger protein 395; F-box protein 16 | up |
| 232935_at | --- | Primary neuroblastoma cDNA, clone:Nbla03614, full insert sequence | up |
| 232940_s_at | MLL3 | myeloid/lymphoid or mixed-lineage leukemia 3 | up |
| 232957_x_at | --- | CDNA FLJ13017 fis, clone NT2RP3000628 | up |
| 233014_at | --- | CDNA FLJ12918 fis, clone NT2RP2004580 | up |
| 233017_x_at | --- | CDNA FLJ12326 fis, clone MAMMA1002132 | up |
| 233226_at | PTPN9 | Protein tyrosine phosphatase, non-receptor type 9 | up |
| 233265_at | --- | CDNA FLJ12203 fis, clone MAMMA1000914 | up |
| 233315_at | --- | CDNA: FLJ21294 fis, clone COL01981 | up |
| 233319_x_at | --- | CDNA FLJ13845 fis, clone THYRO1000815 | up |
| 233321_x_at | LOC90834 | hypothetical protein BC001742 | up |
| 233427_x_at | --- | CDNA FLJ13808 fis, clone THYRO1000253 | up |
| 233449_at | --- | CDNA FLJ11377 fis, clone HEMBA1000442 | up |
| 233608_at | --- | CDNA FLJ11929 fis, clone HEMBB1000434 | up |
| 233622_x_at | --- | MRNA; cDNA DKFZp761A219 (from clone DKFZp761A219) | up |
| 233702_x_at | --- | CDNA: FLJ20946 fis, clone ADSE01819 | up |
| 233877_at | --- | CDNA FLJ20770 fis, clone COL06509 | up |
| 234135_x_at | --- | CDNA FLJ11590 fis, clone HEMBA1003758 | up |
| 234159_at | --- | CDNA: FLJ21529 fis, clone COL05981 | up |
| 234382_x_at | --- | --- | up |
| 234491_s_at | SAV1 | salvador homolog 1 (Drosophila) | up |
| 234762_x_at | NLN | Neurolysin (metallopeptidase M3 family) | up |
| 234981_x_at | CMBL | carboxymethylenebutenolidase homolog (Pseudomonas) | up |
| 234989_at | TncRNA | trophoblast-derived noncoding RNA | up |
| 235041_at | GOSR2 | golgi SNAP receptor complex member 2 | up |
| 235081_x_at | TRIM65 | tripartite motif-containing 65 | up |
| 235084_x_at | --- | Transcribed locus | up |
| 235308_at | ZBTB20 | zinc finger and BTB domain containing 20 | up |
| 235652_at | --- | CDNA FLJ37623 fis, clone BRCOC2014013 | up |
| 235660_at | --- | MRNA; cDNA DKFZp667E0114 (from clone DKFZp667E0114) | up |
| 235847_at | --- | Transcribed locus | up |
| 235999_at | --- | Transcribed locus | up |
| 236041_at | --- | CDNA FLJ33236 fis, clone ASTRO2002571 | up |
| 236060_at | --- | Transcribed locus | up |
| 236327_at | --- | --- | up |
| 236558_at | --- | --- | up |
| 236617_at | --- | Transcribed locus | up |
| 236766_at | C8orf38 | Chromosome 8 open reading frame 38 | up |
| 236841_at | FAM39DP | Family with sequence similarity 39, member D pseudogene | up |
| 236923_x_at | --- | --- | up |
| 236966_at | ARMC8 | armadillo repeat containing 8 | up |
| 237035_at | --- | Transcribed locus | up |
| 237118_at | --- | --- | up |
| 237383_at | --- | Transcribed locus | up |
| 237475_x_at | SEPP1 | Selenoprotein P, plasma, 1 | up |
| 237586_at | --- | --- | up |
| 237733_at | --- | --- | up |
| 237864_at | --- | CDNA FLJ26101 fis, clone SLV05922 | up |
| 238058_at | --- | --- | up |
| 238447_at | RBMS3 | RNA binding motif, single stranded interacting protein | up |
| 238642_at | --- | --- | up |
| 238672_at | --- | Transcribed locus | up |
| 238761_at | --- | Transcribed locus | up |
| 238863_x_at | --- | Transcribed locus | up |
| 238884_at | --- | Transcribed locus | up |
| 238964_at | --- | Transcribed locus | up |
| 239167_at | --- | Transcribed locus | up |
| 239171_at | --- | --- | up |
| 239296_at | --- | Transcribed locus | up |
| 239385_at | TFG | TRK-fused gene | up |
| 239448_at | --- | Transcribed locus | up |
| 239629_at | CFLAR | CASP8 and FADD-like apoptosis regulator | up |
| 239661_at | --- | Transcribed locus | up |
| 239748_x_at | OCIAD1 | OCIA domain containing 1 | up |
| 239753_at | LOC441383 | hypothetical gene supported by AF086559; BC065734 | up |
| 239804_at | --- | Transcribed locus | up |
| 239851_at | --- | --- | up |
| 239956_at | --- | Transcribed locus | up |
| 240139_at | --- | Transcribed locus | up |
| 240146_at | --- | --- | up |
| 240168_at | --- | --- | up |
| 240174_at | --- | Transcribed locus | up |
| 240544_at | --- | Transcribed locus | up |
| 240665_at | --- | --- | up |
| 240773_at | --- | Transcribed locus | up |
| 241223_x_at | --- | Transcribed locus, weakly similar to NP_001013658.1 protein LOC387873 [Homo sapiens] | up |
| 241303_x_at | --- | --- | up |
| 241336_at | --- | --- | up |
| 241347_at | KIAA1618 | KIAA1618 | up |
| 241445_at | --- | Transcribed locus | up |
| 241464_s_at | --- | Transcribed locus | up |
| 241542_at | --- | --- | up |
| 241790_at | --- | Transcribed locus | up |
| 241797_at | --- | --- | up |
| 241818_at | --- | Transcribed locus | up |
| 241843_at | SNORA28 | small nucleolar RNA, H/ACA box 28 | up |
| 241987_x_at | MGC39715 | hypothetical protein MGC39715 | up |
| 242077_x_at | C6orf150 | chromosome 6 open reading frame 150 | up |
| 242099_at | --- | --- | up |
| 242106_at | --- | Transcribed locus | up |
| 242167_at | --- | --- | up |
| 242232_at | --- | --- | up |
| 242235_x_at | NRD1 | Nardilysin (N-arginine dibasic convertase) | up |
| 242261_at | --- | --- | up |
| 242280_x_at | CPEB4 | cytoplasmic polyadenylation element binding protein 4 | up |
| 242320_at | --- | Homo sapiens, clone IMAGE:4769230, mRNA | up |
| 242364_x_at | --- | CDNA clone IMAGE:5286005 | up |
| 242377_x_at | THUMPD3 | THUMP domain containing 3 | up |
| 242405_at | --- | Transcribed locus | up |
| 242416_at | --- | --- | up |
| 242431_at | --- | --- | up |
| 242461_at | --- | --- | up |
| 242472_x_at | FNBP4 | Formin binding protein 4 | up |
| 242480_at | --- | Transcribed locus | up |
| 242558_at | --- | CDNA FLJ45490 fis, clone BRTHA2005831 | up |
| 242579_at | --- | Transcribed locus | up |
| 242622_x_at | PTEN | Phosphatase and tensin homolog (mutated in multiple advanced cancers 1) | up |
| 242645_at | --- | Full length insert cDNA clone YR92A01 | up |
| 242664_at | --- | --- | up |
| 242671_at | --- | --- | up |
| 242859_at | --- | --- | up |
| 242889_x_at | LOC645431 | hypothetical protein LOC645431 | up |
| 242903_at | IFNGR1 | interferon gamma receptor 1 | up |
| 243006_at | --- | CDNA FLJ30333 fis, clone BRACE2007262 | up |
| 243169_at | --- | --- | up |
| 243291_at | --- | Transcribed locus | up |
| 243295_at | RBM27 | RNA binding motif protein 27 | up |
| 243365_s_at | AUTS2 | autism susceptibility candidate 2 | up |
| 243431_at | --- | Transcribed locus | up |
| 243442_x_at | --- | Transcribed locus | up |
| 243546_at | --- | Transcribed locus | up |
| 243612_at | NSD1 | Nuclear receptor binding SET domain protein 1 | up |
| 243640_x_at | --- | --- | up |
| 243648_at | --- | --- | up |
| 243826_at | --- | Transcribed locus | up |
| 243963_at | SDCCAG8 | Serologically defined colon cancer antigen 8 | up |
| 243964_at | --- | Transcribed locus | up |
| 243997_x_at | --- | Transcribed locus | up |
| 244062_at | DAAM1 | dishevelled associated activator of morphogenesis 1 | up |
| 244093_at | --- | --- | up |
| 244197_x_at | --- | --- | up |
| 244345_at | CADM1 | cell adhesion molecule 1 | up |
| 244358_at | --- | --- | up |
| 244457_at | --- | Transcribed locus | up |
| 244459_at | --- | Transcribed locus | up |
| 244480_at | --- | Transcribed locus | up |
| 244535_at | --- | Transcribed locus | up |
| 244579_at | --- | Transcribed locus | up |
| 244697_at | --- | --- | up |
| 244753_at | --- | Transcribed locus | up |
| 244826_at | --- | Transcribed locus | up |
| 244868_at | --- | --- | up |
| 34697_at | LRP6 | low density lipoprotein receptor-related protein 6 | up |
| 41512_at | --- | Transcribed locus | up |
|  |  |  |  |
| **Progressive Supranuclear Palsy** | | | |
| Probe Set ID | Gene Symbol | Gene Title | Expression |
| 1553613_s_at | FOXC1 | forkhead box C1 | up |
| 1553703_at | ZNF791 | zinc finger protein 791 | up |
| 1553704_x_at | ZNF791 | zinc finger protein 791 | up |
| 1554595_at | SYMPK | symplekin | up |
| 1555014_x_at | --- | OK/SW-cl.92 | up |
| 1555192_at | ZNF277P | zinc finger protein 277 pseudogene | up |
| 1555653_at | HNRPA3 | heterogeneous nuclear ribonucleoprotein A3 | up |
| 1555922_at | C10orf114 | chromosome 10 open reading frame 114 | up |
| 1556331_a_at | --- | CDNA clone IMAGE:5259142 | up |
| 1556442_x_at | --- | CDNA FLJ13882 fis, clone THYRO1001480 | up |
| 1556762_a_at | --- | CDNA FLJ30197 fis, clone BRACE2001423 | up |
| 1557394_at | DLGAP4 | discs, large (Drosophila) homolog-associated protein 4 | up |
| 1557477_at | --- | CDNA FLJ33037 fis, clone THYMU2000317 | up |
| 1557585_at | ATP6V1H | ATPase, H+ transporting, lysosomal 50/57kDa, V1 subunit H | up |
| 1557586_s_at | ATP6V1H | ATPase, H+ transporting, lysosomal 50/57kDa, V1 subunit H | up |
| 1557745_at | --- | CDNA FLJ25178 fis, clone CBR09176 | up |
| 1558569_at | --- | MRNA; cDNA DKFZp667K1619 (from clone DKFZp667K1619) | up |
| 1558621_at | CABLES1 | Cdk5 and Abl enzyme substrate 1 | up |
| 1558783_at | --- | CDNA: FLJ21152 fis, clone CAS09594 | up |
| 1558822_at | --- | Full length insert cDNA clone YP59C02 | up |
| 1559156_at | --- | MRNA; cDNA DKFZp686B1142 (from clone DKFZp686B1142) | up |
| 1559375_s_at | --- | Full length insert cDNA clone YI45C08 | up |
| 1559410_at | --- | CDNA FLJ34677 fis, clone LIVER2002660 | up |
| 1559436_x_at | --- | MRNA; cDNA DKFZp313M2114 (from clone DKFZp313M2114) | up |
| 1559820_at | ATG10 | ATG10 autophagy related 10 homolog (S. cerevisiae) | up |
| 1560018_at | ARPP-21 | cyclic AMP-regulated phosphoprotein, 21 kD | up |
| 1560445_x_at | ARHGEF1 | Rho guanine nucleotide exchange factor (GEF) 1 | up |
| 1560512_at | --- | CDNA FLJ30409 fis, clone BRACE2008615 | up |
| 1561139_at | --- | Full length insert cDNA clone ZD67D12 | up |
| 1561167_at | --- | Full length insert cDNA clone YA75A09 | up |
| 1561195_at | --- | MRNA; cDNA DKFZp686A22111 (from clone DKFZp686A22111) | up |
| 1561346_at | --- | CDNA FLJ32691 fis, clone TESTI2000221 | up |
| 1561657_at | --- | Full length insert cDNA clone YZ55H04 | up |
| 1562063_x_at | KIAA1245; LOC728895; NBPF1; NBPF10; NBPF11; NBPF20; NBPF3; NBPF8; NBPF9; XXyac-YX155B6.1 | neuroblastoma breakpoint family, member 1; neuroblastoma breakpoint family, member 3; KIAA1245; neuroblastoma breakpoint family, member 11; neuroblastoma breakpoint family, member 20; neuroblastoma breakpoint family, member 9; neuroblastoma breakpoint family, member 10; neuroblastoma breakpoint family, member 8; hypothetical protein LOC728895; CLIP-190-like | up |
| 1562235_s_at | --- | Transcribed locus | up |
| 1562905_at | --- | CDNA clone IMAGE:5311591 | up |
| 1562955_at | --- | Homo sapiens, clone IMAGE:5396455, mRNA | up |
| 1566491_at | --- | MRNA; cDNA DKFZp547C018 (from clone DKFZp547C018) | up |
| 1566887_x_at | --- | Multiple myeloma susceptibility mRNA sequence | up |
| 1568986_x_at | PIGT | phosphatidylinositol glycan anchor biosynthesis, class T | up |
| 1569519_at | KIAA1245; LOC728895; NBPF1; NBPF10; NBPF11; NBPF20; NBPF8; NBPF9; XXyac-YX155B6.1 | neuroblastoma breakpoint family, member 1; KIAA1245; neuroblastoma breakpoint family, member 11; neuroblastoma breakpoint family, member 20; neuroblastoma breakpoint family, member 9; neuroblastoma breakpoint family, member 10; neuroblastoma breakpoint family, member 8; hypothetical protein LOC728895; CLIP-190-like | up |
| 1569578_at | --- | Homo sapiens, clone IMAGE:4516734, mRNA | up |
| 1570414_x_at | FLJ13197 | hypothetical FLJ13197 | up |
| 201429_s_at | PLK1; RPL37A | polo-like kinase 1 (Drosophila); ribosomal protein L37a | up |
| 201904_s_at | CTDSPL | CTD (carboxy-terminal domain, RNA polymerase II, polypeptide A) small phosphatase-like | up |
| 202481_at | DHRS3 | dehydrogenase/reductase (SDR family) member 3 | up |
| 202734_at | TRIP10 | thyroid hormone receptor interactor 10 | up |
| 202796_at | SYNPO | synaptopodin | up |
| 203408_s_at | SATB1 | SATB homeobox 1 | up |
| 203488_at | LPHN1 | latrophilin 1 | up |
| 203628_at | IGF1R | insulin-like growth factor 1 receptor | up |
| 204223_at | PRELP | proline/arginine-rich end leucine-rich repeat protein | up |
| 204621_s_at | NR4A2 | nuclear receptor subfamily 4, group A, member 2 | up |
| 205887_x_at | MSH3 | mutS homolog 3 (E. coli) | up |
| 205902_at | KCNN3 | potassium intermediate/small conductance calcium-activated channel, subfamily N, member 3 | up |
| 206056_x_at | SPN | sialophorin (leukosialin, CD43) | up |
| 206278_at | PTAFR | platelet-activating factor receptor | up |
| 206374_at | DUSP8 | dual specificity phosphatase 8 | up |
| 206531_at | DPF1 | D4, zinc and double PHD fingers family 1 | up |
| 206548_at | FLJ23556 | hypothetical protein FLJ23556 | up |
| 206565_x_at | SMA3 | SMA3 | up |
| 206792_x_at | PDE4C | phosphodiesterase 4C, cAMP-specific (phosphodiesterase E1 dunce homolog, Drosophila) | up |
| 207499_x_at | UNC45A | unc-45 homolog A (C. elegans) | up |
| 208475_at | FRMD4A | FERM domain containing 4A | up |
| 209121_x_at | NR2F2 | nuclear receptor subfamily 2, group F, member 2 | up |
| 209651_at | TGFB1I1 | transforming growth factor beta 1 induced transcript 1 | up |
| 209866_s_at | LPHN3 | latrophilin 3 | up |
| 209982_s_at | NRXN2 | neurexin 2 | up |
| 210210_at | MPZL1 | myelin protein zero-like 1 | up |
| 210528_at | MR1 | major histocompatibility complex, class I-related | up |
| 211775_x_at | MGC13053 | hypothetical MGC13053 | up |
| 212303_x_at | --- | --- | up |
| 212512_s_at | CARM1 | coactivator-associated arginine methyltransferase 1 | up |
| 212520_s_at | SMARCA4 | SWI/SNF related, matrix associated, actin dependent regulator of chromatin, subfamily a, member 4 | up |
| 212553_at | KIAA0460 | KIAA0460 | up |
| 212762_s_at | TCF7L2 | transcription factor 7-like 2 (T-cell specific, HMG-box) | up |
| 213531_s_at | RAB3GAP1 | RAB3 GTPase activating protein subunit 1 (catalytic) | up |
| 213675_at | --- | CDNA FLJ25106 fis, clone CBR01467 | up |
| 214004_s_at | VGLL4 | vestigial like 4 (Drosophila) | up |
| 214707_x_at | ALMS1 | Alstrom syndrome 1 | up |
| 214989_x_at | --- | CDNA FLJ11875 fis, clone HEMBA1007078 | up |
| 215067_x_at | PRDX2 | peroxiredoxin 2 | up |
| 215147_at | --- | Clone 23712 mRNA sequence | up |
| 215200_x_at | --- | UG0651E06 | up |
| 215372_x_at | --- | CDNA FLJ12002 fis, clone HEMBB1001536 | up |
| 215383_x_at | SPG21 | spastic paraplegia 21 (autosomal recessive, Mast syndrome) | up |
| 215386_at | --- | CDNA FLJ12396 fis, clone MAMMA1002758 | up |
| 215418_at | PARVA | parvin, alpha | up |
| 215435_at | --- | CDNA FLJ11921 fis, clone HEMBB1000318 | up |
| 215455_at | TIMELESS | timeless homolog (Drosophila) | up |
| 215600_x_at | FBXW12 | F-box and WD repeat domain containing 12 | up |
| 215615_x_at | --- | CDNA FLJ14152 fis, clone MAMMA1003089 | up |
| 215810_x_at | D6S1101 | Dystonia musculorum of mouse, human homolog of | up |
| 216176_at | HCRP1 | hepatocellular carcinoma-related HCRP1 | up |
| 216187_x_at | --- | Alu repeat (LNX1) mRNA sequence | up |
| 216259_at | --- | Clone IMAGE:35527 unknown protein | up |
| 216509_x_at | MLLT10 | myeloid/lymphoid or mixed-lineage leukemia (trithorax homolog, Drosophila); translocated to, 10 | up |
| 216524_x_at | --- | MRNA; cDNA DKFZp564E233 (from clone DKFZp564E233) | up |
| 217446_x_at | --- | MRNA; cDNA DKFZp434M054 (from clone DKFZp434M054) | up |
| 217550_at | ATF6 | Activating transcription factor 6 | up |
| 217643_x_at | --- | --- | up |
| 217679_x_at | --- | --- | up |
| 217713_x_at | --- | --- | up |
| 217715_x_at | --- | --- | up |
| 217810_x_at | LARS | leucyl-tRNA synthetase | up |
| 218131_s_at | GATAD2A | GATA zinc finger domain containing 2A | up |
| 218418_s_at | ANKRD25 | ankyrin repeat domain 25 | up |
| 219045_at | RHOF | ras homolog gene family, member F (in filopodia) | up |
| 219290_x_at | DAPP1 | dual adaptor of phosphotyrosine and 3-phosphoinositides | up |
| 219392_x_at | PRR11 | proline rich 11 | up |
| 219975_x_at | OLAH | oleoyl-ACP hydrolase | up |
| 220071_x_at | CEP27 | centrosomal protein 27kDa | up |
| 220612_at | --- | Clone HQ0641 PRO0641 | up |
| 220791_x_at | SCN11A | sodium channel, voltage-gated, type XI, alpha subunit | up |
| 221141_x_at | EPN1 | epsin 1 | up |
| 222104_x_at | GTF2H3 | general transcription factor IIH, polypeptide 3, 34kDa | up |
| 222159_at | --- | CDNA FLJ12996 fis, clone NT2RP3000235 | up |
| 222282_at | --- | Transcribed locus | up |
| 222762_x_at | LIMD1 | LIM domains containing 1 | up |
| 224105_x_at | --- | Clone FLB8034 PRO2158 | up |
| 224259_at | WNT8A | wingless-type MMTV integration site family, member 8A | up |
| 224372_at | IQWD1; UNC5B | IQ motif and WD repeats 1; unc-5 homolog B (C. elegans) | up |
| 224991_at | CMIP | c-Maf-inducing protein | up |
| 225117_at | KIAA1267 | KIAA1267 | up |
| 225570_at | SLC41A1 | solute carrier family 41, member 1 | up |
| 225906_at | --- | CDNA FLJ38264 fis, clone FCBBF3001657 | up |
| 226144_at | REXO1 | REX1, RNA exonuclease 1 homolog (S. cerevisiae) | up |
| 226372_at | CHST11 | Carbohydrate (chondroitin 4) sulfotransferase 11 | up |
| 226554_at | ZBTB7A | zinc finger and BTB domain containing 7A | up |
| 227651_at | BTBD14B | BTB (POZ) domain containing 14B | up |
| 227727_at | MRGPRF | MAS-related GPR, member F | up |
| 227923_at | SHANK3 | SH3 and multiple ankyrin repeat domains 3 | up |
| 228070_at | --- | CDNA FLJ34250 fis, clone FCBBF4000529 | up |
| 228224_at | PRELP | proline/arginine-rich end leucine-rich repeat protein | up |
| 229220_x_at | NOM1 | nucleolar protein with MIF4G domain 1 | up |
| 229265_at | ATN1 | Atrophin 1 | up |
| 229943_at | TRIM13 | tripartite motif-containing 13 | up |
| 230440_at | ZNF469 | zinc finger protein 469 | up |
| 230528_s_at | MGC2752 | hypothetical protein MGC2752 | up |
| 230779_at | TNRC6B | trinucleotide repeat containing 6B | up |
| 230843_at | --- | Transcribed locus | up |
| 231109_at | --- | CDNA FLJ38468 fis, clone FEBRA2021864 | up |
| 231992_x_at | --- | CDNA clone IMAGE:4722553 | up |
| 232015_at | FAM59B | family with sequence similarity 59, member B | up |
| 232096_x_at | --- | CDNA: FLJ22140 fis, clone HEP20977 | up |
| 232169_x_at | NDUFS8 | NADH dehydrogenase (ubiquinone) Fe-S protein 8, 23kDa (NADH-coenzyme Q reductase) | up |
| 232215_x_at | PRR11 | proline rich 11 | up |
| 232225_at | --- | CDNA FLJ11764 fis, clone HEMBA1005685 | up |
| 232347_x_at | --- | CDNA FLJ11379 fis, clone HEMBA1000469 | up |
| 232396_at | --- | MRNA full length insert cDNA clone EUROIMAGE 113222 | up |
| 232416_at | BRUNOL5 | bruno-like 5, RNA binding protein (Drosophila) | up |
| 232420_x_at | LOC286260 | hypothetical protein LOC286260 | up |
| 232455_x_at | LOC340085 | hypothetical protein LOC340085 | up |
| 232554_at | LRRC56 | leucine rich repeat containing 56 | up |
| 232653_at | --- | CDNA FLJ14044 fis, clone HEMBA1006124 | up |
| 232665_x_at | --- | --- | up |
| 232753_at | ZNF346 | Zinc finger protein 346 | up |
| 232882_at | --- | CDNA FLJ12289 fis, clone MAMMA1001788 | up |
| 232935_at | --- | Primary neuroblastoma cDNA, clone:Nbla03614, full insert sequence | up |
| 232957_x_at | --- | CDNA FLJ13017 fis, clone NT2RP3000628 | up |
| 233017_x_at | --- | CDNA FLJ12326 fis, clone MAMMA1002132 | up |
| 233025_at | PDZD2 | PDZ domain containing 2 | up |
| 233041_x_at | --- | CDNA: FLJ21356 fis, clone COL02831 | up |
| 233130_at | --- | CDNA FLJ12202 fis, clone MAMMA1000908 | up |
| 233306_at | --- | CDNA FLJ11447 fis, clone HEMBA1001383 | up |
| 233313_at | --- | CDNA FLJ14302 fis, clone PLACE2000003 | up |
| 233319_x_at | --- | CDNA FLJ13845 fis, clone THYRO1000815 | up |
| 233321_x_at | LOC90834 | hypothetical protein BC001742 | up |
| 233417_at | --- | CDNA FLJ11625 fis, clone HEMBA1004200 | up |
| 233442_at | --- | CDNA FLJ12196 fis, clone MAMMA1000867 | up |
| 233596_at | --- | Clone FLB2543 | up |
| 233605_x_at | HNRPM | heterogeneous nuclear ribonucleoprotein M | up |
| 233622_x_at | --- | MRNA; cDNA DKFZp761A219 (from clone DKFZp761A219) | up |
| 233664_at | --- | CDNA: FLJ22803 fis, clone KAIA2685 | up |
| 233702_x_at | --- | CDNA: FLJ20946 fis, clone ADSE01819 | up |
| 233873_x_at | PAPD1 | PAP associated domain containing 1 | up |
| 234033_at | --- | Clone IMAGE:110218 mRNA sequence | up |
| 234126_at | --- | MRNA; cDNA DKFZp761M1112 (from clone DKFZp761M1112) | up |
| 234127_at | --- | CDNA FLJ13320 fis, clone OVARC1001611 | up |
| 234294_x_at | GATAD2A | GATA zinc finger domain containing 2A | up |
| 234307_s_at | KIF26A | kinesin family member 26A | up |
| 234314_at | C20orf74 | chromosome 20 open reading frame 74 | up |
| 234382_x_at | --- | --- | up |
| 234501_x_at | --- | MRNA; cDNA DKFZp586M151 (from clone DKFZp586M151) | up |
| 234981_x_at | CMBL | carboxymethylenebutenolidase homolog (Pseudomonas) | up |
| 235081_x_at | TRIM65 | tripartite motif-containing 65 | up |
| 235084_x_at | --- | Transcribed locus | up |
| 235538_at | --- | CDNA FLJ30718 fis, clone FCBBF2001675 | up |
| 235803_at | --- | Transcribed locus | up |
| 235875_at | --- | Transcribed locus | up |
| 235985_at | --- | Transcribed locus | up |
| 235990_at | --- | CDNA FLJ38836 fis, clone MESAN2002519, weakly similar to Mus musculus cell cycle checkpoint control protein Mrad9 gene | up |
| 236229_at | --- | Transcribed locus | up |
| 236593_at | --- | Transcribed locus, weakly similar to XP_341471.3 similar to WD repeat domain 17 [Rattus norvegicus] | up |
| 236679_x_at | --- | --- | up |
| 236923_x_at | --- | --- | up |
| 237398_at | --- | Transcribed locus | up |
| 237475_x_at | SEPP1 | Selenoprotein P, plasma, 1 | up |
| 237491_at | --- | --- | up |
| 238152_at | MGC3032 | hypothetical protein MGC3032 | up |
| 238430_x_at | SLFN5 | schlafen family member 5 | up |
| 238712_at | --- | Transcribed locus | up |
| 239058_at | --- | Transcribed locus | up |
| 239091_at | --- | Transcribed locus | up |
| 239361_at | --- | Transcribed locus | up |
| 239748_x_at | OCIAD1 | OCIA domain containing 1 | up |
| 240125_at | --- | Transcribed locus | up |
| 240138_at | --- | Transcribed locus | up |
| 240165_at | --- | Transcribed locus | up |
| 240174_at | --- | Transcribed locus | up |
| 240205_x_at | --- | --- | up |
| 240279_at | --- | --- | up |
| 240651_at | --- | Transcribed locus | up |
| 240665_at | --- | --- | up |
| 240666_at | --- | Transcribed locus | up |
| 240798_at | --- | --- | up |
| 240870_at | --- | --- | up |
| 241303_x_at | --- | --- | up |
| 241585_at | LRRC4C | leucine rich repeat containing 4C | up |
| 241818_at | --- | Transcribed locus | up |
| 242022_at | --- | --- | up |
| 242077_x_at | C6orf150 | chromosome 6 open reading frame 150 | up |
| 242121_at | RNF12 | Ring finger protein 12 | up |
| 242188_at | --- | --- | up |
| 242235_x_at | NRD1 | Nardilysin (N-arginine dibasic convertase) | up |
| 242268_at | CUGBP2 | CUG triplet repeat, RNA binding protein 2 | up |
| 242280_x_at | CPEB4 | cytoplasmic polyadenylation element binding protein 4 | up |
| 242320_at | --- | Homo sapiens, clone IMAGE:4769230, mRNA | up |
| 242364_x_at | --- | CDNA clone IMAGE:5286005 | up |
| 242377_x_at | THUMPD3 | THUMP domain containing 3 | up |
| 242405_at | --- | Transcribed locus | up |
| 242407_at | --- | --- | up |
| 242443_at | EML5 | Echinoderm microtubule associated protein like 5 | up |
| 242471_at | --- | Clone HLS_IMAGE_238756 mRNA sequence | up |
| 242551_at | --- | --- | up |
| 242578_x_at | SLC22A3 | Solute carrier family 22 (extraneuronal monoamine transporter), member 3 | up |
| 242611_at | --- | Transcribed locus | up |
| 242664_at | --- | --- | up |
| 242846_at | --- | Transcribed locus | up |
| 242865_at | --- | --- | up |
| 242872_at | CIT | citron (rho-interacting, serine/threonine kinase 21) | up |
| 242889_x_at | LOC645431 | hypothetical protein LOC645431 | up |
| 243158_at | --- | --- | up |
| 243218_at | --- | Transcribed locus | up |
| 243365_s_at | AUTS2 | autism susceptibility candidate 2 | up |
| 243442_x_at | --- | Transcribed locus | up |
| 243586_at | --- | Transcribed locus | up |
| 243618_s_at | LOC152485 | Hypothetical protein LOC152485 | up |
| 243640_x_at | --- | --- | up |
| 243929_at | --- | --- | up |
| 244217_at | --- | Transcribed locus | up |
| 244310_at | --- | Full length insert cDNA clone YU07D01 | up |
| 244345_at | CADM1 | cell adhesion molecule 1 | up |
| 244358_at | --- | --- | up |
| 244373_at | --- | --- | up |
| 244433_at | --- | --- | up |
| 244457_at | --- | Transcribed locus | up |
| 244535_at | --- | Transcribed locus | up |
| 244605_at | --- | --- | up |
| 244646_at | --- | Transcribed locus | up |
| 244697_at | --- | --- | up |
| 244726_at | --- | Transcribed locus | up |
| 40016_g_at | MAST4 | microtubule associated serine/threonine kinase family member 4 | up |
| 40562_at | GNA11 | guanine nucleotide binding protein (G protein), alpha 11 (Gq class) | up |
| 41160_at | MBD3 | methyl-CpG binding domain protein 3 | up |
| 41644_at | SASH1 | SAM and SH3 domain containing 1 | up |

Table S1. Overview of genes detected to be significantly different from background levels determined using non-demented controls. Amounts of significant different probes are given per pathology-defined group. Alzheimer's disease: AD, Pick’s disease: PiD, Frontotemporal dementia: FTD and progressive supranuclear palsy: PSP. When possible probes are named using official gene symbol names. Gene symbol: official genbank gene symbol. Gene Title: official genbank gene name. ---: Unknown
