# Supplementary material for: Comprehensive mRNA Expression Profiling Distinguishes Tauopathies and Identifies Shared Molecular Pathways
Source: PLoS One. 2009 Aug 28;4(8):e6826. doi: 10.1371/journal.pone.0006826 (PMC2729393; doi:10.1371/journal.pone.0006826)
Supplement: Table S2 — Compiled table of all 790 probes detected to be significantly different from background and overlapping in at least two different pathologically defined groups. When possible probes are given in official gene symbol names. Gene symbol: official genbank gene symbol. Gene Title: official genbank gene name. ---: Unknown (0.83 MB DOC) [file pone.0006826.s002.doc]

| **Total gene set of significant probes / genes** | | |
| --- | --- | --- |
| Probe Set ID | Gene Symbol | Gene Title |
| 1552256_a_at | SCARB1 | scavenger receptor class B, member 1 |
| 1552301_a_at | CORO6 | coronin 6 |
| 1552536_at | VTI1A | vesicle transport through interaction with t-SNAREs homolog 1A (yeast) |
| 1552739_s_at | ST7L | suppression of tumorigenicity 7 like |
| 1552957_at | LOC200383 | similar to Dynein heavy chain at 16F |
| 1553150_at | AOF1 | amine oxidase (flavin containing) domain 1 |
| 1553407_at | MACF1 | microtubule-actin crosslinking factor 1 |
| 1553479_at | TMEM145 | transmembrane protein 145 |
| 1553565_s_at | DDAH1 | dimethylarginine dimethylaminohydrolase 1 |
| 1553613_s_at | FOXC1 | forkhead box C1 |
| 1553703_at | ZNF791 | zinc finger protein 791 |
| 1553704_x_at | ZNF791 | zinc finger protein 791 |
| 1553796_at | FLJ30594 | hypothetical locus FLJ30594 |
| 1553995_a_at | NT5E | 5'-nucleotidase, ecto (CD73) |
| 1554473_at | SRGAP1 | SLIT-ROBO Rho GTPase activating protein 1 |
| 1554474_a_at | MOXD1 | monooxygenase, DBH-like 1 |
| 1554595_at | SYMPK | symplekin |
| 1554679_a_at | LAPTM4B | lysosomal associated protein transmembrane 4 beta |
| 1554703_at | ARHGEF10 | Rho guanine nucleotide exchange factor (GEF) 10 |
| 1554747_a_at | SEPT2 | septin 2 |
| 1554963_at | --- | CDNA clone IMAGE:5310797 |
| 1555014_x_at | --- | OK/SW-cl.92 |
| 1555192_at | ZNF277P | zinc finger protein 277 pseudogene |
| 1555259_at | ZAK | sterile alpha motif and leucine zipper containing kinase AZK |
| 1555318_at | HIF3A | hypoxia inducible factor 3, alpha subunit |
| 1555372_at | BCL2L11 | BCL2-like 11 (apoptosis facilitator) |
| 1555470_a_at | PPM1F | protein phosphatase 1F (PP2C domain containing) |
| 1555491_a_at | FLJ11286 | hypothetical protein FLJ11286 |
| 1555653_at | HNRPA3 | heterogeneous nuclear ribonucleoprotein A3 |
| 1555827_at | CCNL1 | Cyclin L1 |
| 1555922_at | C10orf114 | chromosome 10 open reading frame 114 |
| 1556000_s_at | BTBD7 | BTB (POZ) domain containing 7 |
| 1556331_a_at | --- | CDNA clone IMAGE:5259142 |
| 1556352_at | --- | CDNA FLJ30440 fis, clone BRACE2009185 |
| 1556442_x_at | --- | CDNA FLJ13882 fis, clone THYRO1001480 |
| 1556551_s_at | SLC39A6 | solute carrier family 39 (zinc transporter), member 6 |
| 1556606_at | NAV2 | neuron navigator 2 |
| 1556650_at | --- | CDNA FLJ13011 fis, clone NT2RP3000561 |
| 1556658_a_at | --- | CDNA FLJ36459 fis, clone THYMU2014762 |
| 1556762_a_at | --- | CDNA FLJ30197 fis, clone BRACE2001423 |
| 1556818_at | --- | Full length insert cDNA clone YB35F05 |
| 1556849_at | --- | CDNA FLJ11909 fis, clone HEMBB1000099 |
| 1557186_s_at | TPCN1 | two pore segment channel 1 |
| 1557394_at | DLGAP4 | discs, large (Drosophila) homolog-associated protein 4 |
| 1557477_at | --- | CDNA FLJ33037 fis, clone THYMU2000317 |
| 1557505_a_at | --- | Full length insert cDNA YQ11E04 |
| 1557585_at | ATP6V1H | ATPase, H+ transporting, lysosomal 50/57kDa, V1 subunit H |
| 1557586_s_at | ATP6V1H | ATPase, H+ transporting, lysosomal 50/57kDa, V1 subunit H |
| 1557690_x_at | --- | CDNA FLJ11951 fis, clone HEMBB1000827 |
| 1557706_at | ZHX2 | zinc fingers and homeoboxes 2 |
| 1557745_at | --- | CDNA FLJ25178 fis, clone CBR09176 |
| 1557803_at | --- | Full length insert cDNA clone YZ56G10 |
| 1557889_at | --- | CDNA clone IMAGE:4138742 |
| 1558009_at | SLC1A2 | solute carrier family 1 (glial high affinity glutamate transporter), member 2 |
| 1558010_s_at | SLC1A2 | solute carrier family 1 (glial high affinity glutamate transporter), member 2 |
| 1558041_a_at | LOC653319 | hypothetical protein LOC653319 |
| 1558569_at | --- | MRNA; cDNA DKFZp667K1619 (from clone DKFZp667K1619) |
| 1558621_at | CABLES1 | Cdk5 and Abl enzyme substrate 1 |
| 1558678_s_at | MALAT1 | metastasis associated lung adenocarcinoma transcript 1 (non-coding RNA) |
| 1558783_at | --- | CDNA: FLJ21152 fis, clone CAS09594 |
| 1558796_a_at | LOC728052; LOC731255 | hypothetical protein LOC728052; hypothetical protein LOC731255 |
| 1558822_at | --- | Full length insert cDNA clone YP59C02 |
| 1558831_x_at | --- | CDNA FLJ34403 fis, clone HCHON2001607 |
| 1558832_at | FLJ32224 | hypothetical gene supported by AK056786 |
| 1558877_at | --- | CDNA FLJ36355 fis, clone THYMU2007384 |
| 1559020_a_at | --- | CDNA FLJ14081 fis, clone HEMBB1002280 |
| 1559156_at | --- | MRNA; cDNA DKFZp686B1142 (from clone DKFZp686B1142) |
| 1559249_at | ATXN1 | Ataxin 1 |
| 1559375_s_at | --- | Full length insert cDNA clone YI45C08 |
| 1559410_at | --- | CDNA FLJ34677 fis, clone LIVER2002660 |
| 1559436_x_at | --- | MRNA; cDNA DKFZp313M2114 (from clone DKFZp313M2114) |
| 1559593_a_at | CRSP7 | Cofactor required for Sp1 transcriptional activation, subunit 7, 70kDa |
| 1559618_at | --- | CDNA FLJ30384 fis, clone BRACE2008114 |
| 1559820_at | ATG10 | ATG10 autophagy related 10 homolog (S. cerevisiae) |
| 1559965_at | --- | CDNA clone IMAGE:4811567 |
| 1559987_at | --- | Homo sapiens, clone IMAGE:5585678, mRNA |
| 1560018_at | ARPP-21 | cyclic AMP-regulated phosphoprotein, 21 kD |
| 1560445_x_at | ARHGEF1 | Rho guanine nucleotide exchange factor (GEF) 1 |
| 1560512_at | --- | CDNA FLJ30409 fis, clone BRACE2008615 |
| 1560659_at | --- | --- |
| 1560661_x_at | --- | --- |
| 1560798_at | --- | CDNA FLJ14121 fis, clone MAMMA1002009 |
| 1560926_at | --- | Full length insert cDNA clone YR43G06 |
| 1561139_at | --- | Full length insert cDNA clone ZD67D12 |
| 1561166_a_at | --- | Full length insert cDNA clone YB22D01 |
| 1561167_at | --- | Full length insert cDNA clone YA75A09 |
| 1561180_at | --- | CDNA FLJ11745 fis, clone HEMBA1005526 |
| 1561195_at | --- | MRNA; cDNA DKFZp686A22111 (from clone DKFZp686A22111) |
| 1561346_at | --- | CDNA FLJ32691 fis, clone TESTI2000221 |
| 1561362_at | --- | CDNA FLJ36285 fis, clone THYMU2003470 |
| 1561657_at | --- | Full length insert cDNA clone YZ55H04 |
| 1561777_at | --- | (TL22) mRNA from LNCaP cell line |
| 1562062_at | KIAA1245; LOC728895; NBPF1; NBPF10; NBPF11; NBPF20; NBPF3; NBPF8; NBPF9; XXyac-YX155B6.1 | neuroblastoma breakpoint family, member 1; neuroblastoma breakpoint family, member 3; KIAA1245; neuroblastoma breakpoint family, member 11; neuroblastoma breakpoint family, member 20; neuroblastoma breakpoint family, member 9; neuroblastoma breakpoint family, member 10; neuroblastoma breakpoint family, member 8; hypothetical protein LOC728895; CLIP-190-like |
| 1562063_x_at | KIAA1245; LOC728895; NBPF1; NBPF10; NBPF11; NBPF20; NBPF3; NBPF8; NBPF9; XXyac-YX155B6.1 | neuroblastoma breakpoint family, member 1; neuroblastoma breakpoint family, member 3; KIAA1245; neuroblastoma breakpoint family, member 11; neuroblastoma breakpoint family, member 20; neuroblastoma breakpoint family, member 9; neuroblastoma breakpoint family, member 10; neuroblastoma breakpoint family, member 8; hypothetical protein LOC728895; CLIP-190-like |
| 1562235_s_at | --- | Transcribed locus |
| 1562583_s_at | LOC646405; LOC651239 | hypothetical LOC646405; hypothetical protein LOC651239 |
| 1562905_at | --- | CDNA clone IMAGE:5311591 |
| 1562948_at | --- | Homo sapiens, clone IMAGE:5722724, mRNA |
| 1562955_at | --- | Homo sapiens, clone IMAGE:5396455, mRNA |
| 1563321_s_at | MLLT10 | myeloid/lymphoid or mixed-lineage leukemia (trithorax homolog, Drosophila); translocated to, 10 |
| 1563482_at | --- | CDNA FLJ26750 fis, clone PRS01773 |
| 1565692_at | --- | CDNA FLJ40647 fis, clone THYMU2017522 |
| 1565743_at | --- | CDNA FLJ37648 fis, clone BRHIP2000532 |
| 1566472_s_at | RETSAT | retinol saturase (all-trans-retinol 13,14-reductase) |
| 1566482_at | --- | MRNA; cDNA DKFZp313L2229 (from clone DKFZp313L2229) |
| 1566491_at | --- | MRNA; cDNA DKFZp547C018 (from clone DKFZp547C018) |
| 1566539_at | --- | MRNA; cDNA DKFZp586G081 (from clone DKFZp586G081) |
| 1566551_at | --- | MRNA; cDNA DKFZp586L2217 (from clone DKFZp586L2217) |
| 1566887_x_at | --- | Multiple myeloma susceptibility mRNA sequence |
| 1568619_s_at | LOC162073 | hypothetical protein LOC162073 |
| 1568866_at | --- | CDNA clone IMAGE:5450715 |
| 1568986_x_at | PIGT | phosphatidylinositol glycan anchor biosynthesis, class T |
| 1569323_at | PTPRG | protein tyrosine phosphatase, receptor type, G |
| 1569477_at | --- | Homo sapiens, clone IMAGE:4291396, mRNA |
| 1569519_at | KIAA1245; LOC728895; NBPF1; NBPF10; NBPF11; NBPF20; NBPF8; NBPF9; XXyac-YX155B6.1 | neuroblastoma breakpoint family, member 1; KIAA1245; neuroblastoma breakpoint family, member 11; neuroblastoma breakpoint family, member 20; neuroblastoma breakpoint family, member 9; neuroblastoma breakpoint family, member 10; neuroblastoma breakpoint family, member 8; hypothetical protein LOC728895; CLIP-190-like |
| 1569578_at | --- | Homo sapiens, clone IMAGE:4516734, mRNA |
| 1569661_at | --- | CDNA clone IMAGE:5260324 |
| 1569948_at | --- | CDNA clone IMAGE:5275301 |
| 1570414_x_at | FLJ13197 | hypothetical FLJ13197 |
| 1570511_at | ARHGEF10L | Rho guanine nucleotide exchange factor (GEF) 10-like |
| 200041_s_at | BAT1 | HLA-B associated transcript 1 |
| 200076_s_at | C19orf50 | chromosome 19 open reading frame 50 |
| 200644_at | MARCKSL1 | MARCKS-like 1 |
| 200754_x_at | SFRS2 | splicing factor, arginine/serine-rich 2 |
| 200778_s_at | SEPT2 | septin 2 |
| 200868_s_at | ZNF313 | zinc finger protein 313 |
| 200898_s_at | MGEA5 | meningioma expressed antigen 5 (hyaluronidase) |
| 200946_x_at | GLUD1 | glutamate dehydrogenase 1 |
| 200980_s_at | PDHA1 | pyruvate dehydrogenase (lipoamide) alpha 1 |
| 201014_s_at | PAICS | phosphoribosylaminoimidazole carboxylase, phosphoribosylaminoimidazole succinocarboxamide synthetase |
| 201103_x_at | LOC728936; LOC728980; NBPF10; NBPF11; NBPF15; NBPF8 | neuroblastoma breakpoint family, member 11; neuroblastoma breakpoint family, member 15; neuroblastoma breakpoint family, member 10; neuroblastoma breakpoint family, member 8; similar to CG10522-PA; hypothetical protein LOC728980 |
| 201116_s_at | CPE | carboxypeptidase E |
| 201135_at | ECHS1 | enoyl Coenzyme A hydratase, short chain, 1, mitochondrial |
| 201185_at | HTRA1 | HtrA serine peptidase 1 |
| 201220_x_at | CTBP2 | C-terminal binding protein 2 |
| 201349_at | SLC9A3R1 | solute carrier family 9 (sodium/hydrogen exchanger), member 3 regulator 1 |
| 201404_x_at | PSMB2 | proteasome (prosome, macropain) subunit, beta type, 2 |
| 201429_s_at | PLK1; RPL37A | polo-like kinase 1 (Drosophila); ribosomal protein L37a |
| 201439_at | GBF1 | golgi-specific brefeldin A resistance factor 1 |
| 201559_s_at | CLIC4 | chloride intracellular channel 4 |
| 201619_at | PRDX3 | peroxiredoxin 3 |
| 201667_at | GJA1 | gap junction protein, alpha 1, 43kDa |
| 201818_at | AYTL2 | acyltransferase like 2 |
| 201867_s_at | TBL1X | transducin (beta)-like 1X-linked |
| 201901_s_at | YY1 | YY1 transcription factor |
| 201904_s_at | CTDSPL | CTD (carboxy-terminal domain, RNA polymerase II, polypeptide A) small phosphatase-like |
| 201996_s_at | SPEN | spen homolog, transcriptional regulator (Drosophila) |
| 202026_at | SDHD | succinate dehydrogenase complex, subunit D, integral membrane protein |
| 202040_s_at | JARID1A | jumonji, AT rich interactive domain 1A |
| 202087_s_at | CTSL1 | cathepsin L1 |
| 202281_at | GAK | cyclin G associated kinase |
| 202449_s_at | RXRA | retinoid X receptor, alpha |
| 202481_at | DHRS3 | dehydrogenase/reductase (SDR family) member 3 |
| 202578_s_at | DDX19A | DEAD (Asp-Glu-Ala-As) box polypeptide 19A |
| 202734_at | TRIP10 | thyroid hormone receptor interactor 10 |
| 202740_at | ACY1 | aminoacylase 1 |
| 202796_at | SYNPO | synaptopodin |
| 202935_s_at | SOX9 | SRY (sex determining region Y)-box 9 (campomelic dysplasia, autosomal sex-reversal) |
| 202975_s_at | RHOBTB3 | Rho-related BTB domain containing 3 |
| 203007_x_at | LYPLA1 | lysophospholipase I |
| 203025_at | ARD1A | ARD1 homolog A, N-acetyltransferase (S. cerevisiae) |
| 203146_s_at | GABBR1 | gamma-aminobutyric acid (GABA) B receptor, 1 |
| 203179_at | GALT | galactose-1-phosphate uridylyltransferase |
| 203229_s_at | CLK2 | CDC-like kinase 2 |
| 203408_s_at | SATB1 | SATB homeobox 1 |
| 203423_at | RBP1 | retinol binding protein 1, cellular |
| 203452_at | B3GAT3 | beta-1,3-glucuronyltransferase 3 (glucuronosyltransferase I) |
| 203488_at | LPHN1 | latrophilin 1 |
| 203496_s_at | PPARBP | PPAR binding protein |
| 203615_x_at | SULT1A1 | sulfotransferase family, cytosolic, 1A, phenol-preferring, member 1 |
| 203628_at | IGF1R | insulin-like growth factor 1 receptor |
| 203668_at | MAN2C1 | mannosidase, alpha, class 2C, member 1 |
| 203790_s_at | HRSP12 | heat-responsive protein 12 |
| 203802_x_at | NSUN5 | NOL1/NOP2/Sun domain family, member 5 |
| 204090_at | STK19 | serine/threonine kinase 19 |
| 204193_at | CHKB; CPT1B | choline kinase beta; carnitine palmitoyltransferase 1B (muscle) |
| 204223_at | PRELP | proline/arginine-rich end leucine-rich repeat protein |
| 204257_at | FADS3 | fatty acid desaturase 3 |
| 204461_x_at | RAD1 | RAD1 homolog (S. pombe) |
| 204538_x_at | LOC339047; LOC642778; LOC642799; NPIP | nuclear pore complex interacting protein; hypothetical protein LOC339047; similar to nuclear pore complex interacting protein |
| 204621_s_at | NR4A2 | nuclear receptor subfamily 4, group A, member 2 |
| 204650_s_at | APBB3 | amyloid beta (A4) precursor protein-binding, family B, member 3 |
| 204786_s_at | IFNAR2 | interferon (alpha, beta and omega) receptor 2 |
| 204864_s_at | IL6ST | interleukin 6 signal transducer (gp130, oncostatin M receptor) |
| 205130_at | RAGE | renal tumor antigen |
| 205187_at | SMAD5 | SMAD family member 5 |
| 205255_x_at | TCF7 | transcription factor 7 (T-cell specific, HMG-box) |
| 205318_at | KIF5A | kinesin family member 5A |
| 205344_at | CSPG5 | chondroitin sulfate proteoglycan 5 (neuroglycan C) |
| 205383_s_at | ZBTB20 | zinc finger and BTB domain containing 20 |
| 205435_s_at | AAK1 | AP2 associated kinase 1 |
| 205662_at | EPPB9 | B9 protein |
| 205839_s_at | BZRAP1 | benzodiazapine receptor (peripheral) associated protein 1 |
| 205887_x_at | MSH3 | mutS homolog 3 (E. coli) |
| 205902_at | KCNN3 | potassium intermediate/small conductance calcium-activated channel, subfamily N, member 3 |
| 206056_x_at | SPN | sialophorin (leukosialin, CD43) |
| 206278_at | PTAFR | platelet-activating factor receptor |
| 206374_at | DUSP8 | dual specificity phosphatase 8 |
| 206527_at | ABAT | 4-aminobutyrate aminotransferase |
| 206531_at | DPF1 | D4, zinc and double PHD fingers family 1 |
| 206541_at | KLKB1 | kallikrein B, plasma (Fletcher factor) 1 |
| 206548_at | FLJ23556 | hypothetical protein FLJ23556 |
| 206565_x_at | SMA3 | SMA3 |
| 206582_s_at | GPR56 | G protein-coupled receptor 56 |
| 206701_x_at | EDNRB | endothelin receptor type B |
| 206792_x_at | PDE4C | phosphodiesterase 4C, cAMP-specific (phosphodiesterase E1 dunce homolog, Drosophila) |
| 206846_s_at | HDAC6 | histone deacetylase 6 |
| 206950_at | SCN9A | sodium channel, voltage-gated, type IX, alpha subunit |
| 207048_at | SLC6A11 | solute carrier family 6 (neurotransmitter transporter, GABA), member 11 |
| 207122_x_at | SULT1A2 | sulfotransferase family, cytosolic, 1A, phenol-preferring, member 2 |
| 207499_x_at | UNC45A | unc-45 homolog A (C. elegans) |
| 207657_x_at | TNPO1 | transportin 1 |
| 207660_at | DMD | dystrophin (muscular dystrophy, Duchenne and Becker types) |
| 208054_at | HERC4 | hect domain and RLD 4 |
| 208238_x_at | --- | --- |
| 208475_at | FRMD4A | FERM domain containing 4A |
| 208591_s_at | PDE3B | phosphodiesterase 3B, cGMP-inhibited |
| 208633_s_at | MACF1 | microtubule-actin crosslinking factor 1 |
| 208634_s_at | MACF1 | microtubule-actin crosslinking factor 1 |
| 208686_s_at | BRD2 | bromodomain containing 2 |
| 208829_at | TAPBP | TAP binding protein (tapasin) |
| 208853_s_at | CANX | calnexin |
| 208871_at | ATN1 | atrophin 1 |
| 208922_s_at | NXF1 | nuclear RNA export factor 1 |
| 209074_s_at | FAM107A | family with sequence similarity 107, member A |
| 209091_s_at | SH3GLB1 | SH3-domain GRB2-like endophilin B1 |
| 209121_x_at | NR2F2 | nuclear receptor subfamily 2, group F, member 2 |
| 209135_at | ASPH | aspartate beta-hydroxylase |
| 209209_s_at | PLEKHC1 | pleckstrin homology domain containing, family C (with FERM domain) member 1 |
| 209225_x_at | TNPO1 | transportin 1 |
| 209393_s_at | EIF4E2 | eukaryotic translation initiation factor 4E family member 2 |
| 209403_at | LOC653380; LOC653498; LOC727735; LOC729837; LOC729873; LOC729877; TBC1D3; TBC1D3C | TBC1 domain family, member 3; TBC1 domain family, member 3C; similar to USP6 N-terminal like; similar to TBC1 domain family member 3 (Rab GTPase-activating protein PRC17) (Prostate cancer gene 17 protein) (TRE17 alpha protein); similar to TBC1 domain family, member 3 |
| 209428_s_at | ZFPL1 | zinc finger protein-like 1 |
| 209437_s_at | SPON1 | spondin 1, extracellular matrix protein |
| 209450_at | OSGEP | O-sialoglycoprotein endopeptidase |
| 209593_s_at | TOR1B | torsin family 1, member B (torsin B) |
| 209623_at | MCCC2 | methylcrotonoyl-Coenzyme A carboxylase 2 (beta) |
| 209651_at | TGFB1I1 | transforming growth factor beta 1 induced transcript 1 |
| 209708_at | MOXD1 | monooxygenase, DBH-like 1 |
| 209866_s_at | LPHN3 | latrophilin 3 |
| 209940_at | PARP3 | poly (ADP-ribose) polymerase family, member 3 |
| 209954_x_at | SS18 | synovial sarcoma translocation, chromosome 18 |
| 209982_s_at | NRXN2 | neurexin 2 |
| 210101_x_at | SH3GLB1 | SH3-domain GRB2-like endophilin B1 |
| 210153_s_at | ME2 | malic enzyme 2, NAD(+)-dependent, mitochondrial |
| 210210_at | MPZL1 | myelin protein zero-like 1 |
| 210407_at | PPM1A | protein phosphatase 1A (formerly 2C), magnesium-dependent, alpha isoform |
| 210528_at | MR1 | major histocompatibility complex, class I-related |
| 210556_at | NFATC3 | nuclear factor of activated T-cells, cytoplasmic, calcineurin-dependent 3 |
| 210738_s_at | SLC4A4 | solute carrier family 4, sodium bicarbonate cotransporter, member 4 |
| 210775_x_at | CASP9 | caspase 9, apoptosis-related cysteine peptidase |
| 210778_s_at | MXD4 | MAX dimerization protein 4 |
| 210794_s_at | MEG3 | maternally expressed 3 |
| 210835_s_at | CTBP2 | C-terminal binding protein 2 |
| 210843_s_at | MFAP3L | microfibrillar-associated protein 3-like |
| 210896_s_at | ASPH | aspartate beta-hydroxylase |
| 211207_s_at | ACSL6 | acyl-CoA synthetase long-chain family member 6 |
| 211276_at | TCEAL2 | transcription elongation factor A (SII)-like 2 |
| 211316_x_at | CFLAR | CASP8 and FADD-like apoptosis regulator |
| 211569_s_at | HADH | hydroxyacyl-Coenzyme A dehydrogenase |
| 211574_s_at | CD46 | CD46 molecule, complement regulatory protein |
| 211713_x_at | KIAA0101 | KIAA0101 |
| 211715_s_at | BDH1 | 3-hydroxybutyrate dehydrogenase, type 1 |
| 211775_x_at | MGC13053 | hypothetical MGC13053 |
| 211876_x_at | PCDHGA10; PCDHGA11; PCDHGA12; PCDHGA3; PCDHGA5; PCDHGA6 | protocadherin gamma subfamily A, 12; protocadherin gamma subfamily A, 11; protocadherin gamma subfamily A, 10; protocadherin gamma subfamily A, 6; protocadherin gamma subfamily A, 5; protocadherin gamma subfamily A, 3 |
| 211890_x_at | CAPN3 | calpain 3, (p94) |
| 212059_s_at | TRPC4AP | transient receptor potential cation channel, subfamily C, member 4 associated protein |
| 212079_s_at | MLL | myeloid/lymphoid or mixed-lineage leukemia (trithorax homolog, Drosophila) |
| 212087_s_at | ERAL1 | Era G-protein-like 1 (E. coli) |
| 212228_s_at | COQ9 | coenzyme Q9 homolog (S. cerevisiae) |
| 212291_at | HIPK1 | homeodomain interacting protein kinase 1 |
| 212303_x_at | --- | --- |
| 212492_s_at | JMJD2B | jumonji domain containing 2B |
| 212512_s_at | CARM1 | coactivator-associated arginine methyltransferase 1 |
| 212520_s_at | SMARCA4 | SWI/SNF related, matrix associated, actin dependent regulator of chromatin, subfamily a, member 4 |
| 212553_at | KIAA0460 | KIAA0460 |
| 212601_at | ZZEF1 | zinc finger, ZZ-type with EF-hand domain 1 |
| 212762_s_at | TCF7L2 | transcription factor 7-like 2 (T-cell specific, HMG-box) |
| 212852_s_at | TROVE2 | TROVE domain family, member 2 |
| 213002_at | MARCKS | Myristoylated alanine-rich protein kinase C substrate |
| 213143_at | LOC257407 | hypothetical protein LOC257407 |
| 213236_at | SASH1 | SAM and SH3 domain containing 1 |
| 213531_s_at | RAB3GAP1 | RAB3 GTPase activating protein subunit 1 (catalytic) |
| 213641_at | ZNF500 | zinc finger protein 500 |
| 213652_at | PCSK5 | Proprotein convertase subtilisin/kexin type 5 |
| 213675_at | --- | CDNA FLJ25106 fis, clone CBR01467 |
| 213872_at | C6orf62 | Chromosome 6 open reading frame 62 |
| 213956_at | CEP350 | centrosomal protein 350kDa |
| 214004_s_at | VGLL4 | vestigial like 4 (Drosophila) |
| 214035_x_at | LOC399491 | LOC399491 protein |
| 214104_at | GPR161 | G protein-coupled receptor 161 |
| 214121_x_at | PDLIM7 | PDZ and LIM domain 7 (enigma) |
| 214176_s_at | PBXIP1 | Pre-B-cell leukemia homeobox interacting protein 1 |
| 214205_x_at | TXNL2 | thioredoxin-like 2 |
| 214241_at | NDUFB8 | NADH dehydrogenase (ubiquinone) 1 beta subcomplex, 8, 19kDa |
| 214329_x_at | TNFSF10 | Tumor necrosis factor (ligand) superfamily, member 10 |
| 214405_at | --- | Clone 23705 mRNA sequence |
| 214427_at | NOL1 | nucleolar protein 1, 120kDa |
| 214564_s_at | PCDHGC3 | protocadherin gamma subfamily C, 3 |
| 214707_x_at | ALMS1 | Alstrom syndrome 1 |
| 214743_at | CUTL1 | cut-like 1, CCAAT displacement protein (Drosophila) |
| 214806_at | BICD1 | bicaudal D homolog 1 (Drosophila) |
| 214882_s_at | SFRS2 | splicing factor, arginine/serine-rich 2 |
| 214902_x_at | --- | MRNA; cDNA DKFZp586A061 (from clone DKFZp586A061) |
| 214929_s_at | KIAA1109 | KIAA1109 |
| 214989_x_at | --- | CDNA FLJ11875 fis, clone HEMBA1007078 |
| 215067_x_at | PRDX2 | peroxiredoxin 2 |
| 215147_at | --- | Clone 23712 mRNA sequence |
| 215200_x_at | --- | UG0651E06 |
| 215253_s_at | DSCR1 | Down syndrome critical region gene 1 |
| 215310_at | APC | Adenomatosis polyposis coli |
| 215350_at | SYNE1 | spectrin repeat containing, nuclear envelope 1 |
| 215372_x_at | --- | CDNA FLJ12002 fis, clone HEMBB1001536 |
| 215383_x_at | SPG21 | spastic paraplegia 21 (autosomal recessive, Mast syndrome) |
| 215385_at | --- | CDNA FLJ12411 fis, clone MAMMA1002964 |
| 215386_at | --- | CDNA FLJ12396 fis, clone MAMMA1002758 |
| 215390_at | --- | CDNA FLJ12102 fis, clone HEMBB1002684 |
| 215418_at | PARVA | parvin, alpha |
| 215435_at | --- | CDNA FLJ11921 fis, clone HEMBB1000318 |
| 215439_x_at | --- | CDNA FLJ11924 fis, clone HEMBB1000343 |
| 215455_at | TIMELESS | timeless homolog (Drosophila) |
| 215507_x_at | --- | Transcribed locus |
| 215588_x_at | RIOK3 | RIO kinase 3 (yeast) |
| 215589_at | --- | CDNA: FLJ21284 fis, clone COL01911 |
| 215595_x_at | --- | CDNA FLJ13856 fis, clone THYRO1000988 |
| 215600_x_at | FBXW12 | F-box and WD repeat domain containing 12 |
| 215615_x_at | --- | CDNA FLJ14152 fis, clone MAMMA1003089 |
| 215653_at | --- | Clone IMAGE:248602, mRNA sequence |
| 215683_at | --- | Clone 24803 mRNA sequence |
| 215698_at | JARID1A | jumonji, AT rich interactive domain 1A |
| 215794_x_at | GLUD2 | glutamate dehydrogenase 2 |
| 215810_x_at | D6S1101 | Dystonia musculorum of mouse, human homolog of |
| 215836_s_at | PCDHGA1; PCDHGA10; PCDHGA11; PCDHGA12; PCDHGA2; PCDHGA3; PCDHGA4; PCDHGA5; PCDHGA6; PCDHGA7; PCDHGA8; PCDHGA9; PCDHGB1; PCDHGB2; PCDHGB3; PCDHGB4; PCDHGB5; PCDHGB6; PCDHGB7; PCDHGC3; PCDHGC4; PCDHGC5 | protocadherin gamma subfamily C, 3; protocadherin gamma subfamily B, 4; protocadherin gamma subfamily A, 8; protocadherin gamma subfamily A, 12; protocadherin gamma subfamily C, 5; protocadherin gamma subfamily C, 4; protocadherin gamma subfamily B, 7; protocadherin gamma subfamily B, 6; protocadherin gamma subfamily B, 5; protocadherin gamma subfamily B, 3; protocadherin gamma subfamily B, 2; protocadherin gamma subfamily B, 1; protocadherin gamma subfamily A, 11; protocadherin gamma subfamily A, 10; protocadherin gamma subfamily A, 9; protocadherin gamma subfamily A, 7; protocadherin gamma subfamily A, 6; protocadherin gamma subfamily A, 5; protocadherin gamma subfamily A, 4; protocadherin gamma subfamily A, 3; protocadherin gamma subfamily A, 2; protocadherin gamma subfamily A, 1 |
| 215982_s_at | DOM3Z | dom-3 homolog Z (C. elegans) |
| 216080_s_at | FADS3 | fatty acid desaturase 3 |
| 216101_at | --- | Full length insert cDNA clone YR67C11 |
| 216123_x_at | --- | CDNA FLJ14096 fis, clone MAMMA1000752 |
| 216176_at | HCRP1 | hepatocellular carcinoma-related HCRP1 |
| 216187_x_at | --- | Alu repeat (LNX1) mRNA sequence |
| 216189_at | --- | Homo sapiens, clone IMAGE:3344506 |
| 216259_at | --- | Clone IMAGE:35527 unknown protein |
| 216294_s_at | KIAA1109 | KIAA1109 |
| 216352_x_at | PCDHGA3 | protocadherin gamma subfamily A, 3 |
| 216509_x_at | MLLT10 | myeloid/lymphoid or mixed-lineage leukemia (trithorax homolog, Drosophila); translocated to, 10 |
| 216524_x_at | --- | MRNA; cDNA DKFZp564E233 (from clone DKFZp564E233) |
| 216532_x_at | LOC643450; LOC728344 | similar to Thioredoxin-like protein 2 (PKC-interacting cousin of thioredoxin) (PKC-theta-interacting protein) (PKCq-interacting protein) |
| 216958_s_at | IVD | isovaleryl Coenzyme A dehydrogenase |
| 217446_x_at | --- | MRNA; cDNA DKFZp434M054 (from clone DKFZp434M054) |
| 217536_x_at | --- | Transcribed locus |
| 217541_x_at | LOC731901; ZNF816A | zinc finger protein 816A; similar to zinc finger protein 160 |
| 217550_at | ATF6 | Activating transcription factor 6 |
| 217643_x_at | --- | --- |
| 217662_x_at | --- | Transcribed locus |
| 217679_x_at | --- | --- |
| 217713_x_at | --- | --- |
| 217715_x_at | --- | --- |
| 217810_x_at | LARS | leucyl-tRNA synthetase |
| 217889_s_at | CYBRD1 | cytochrome b reductase 1 |
| 218059_at | ZNF706 | zinc finger protein 706 |
| 218131_s_at | GATAD2A | GATA zinc finger domain containing 2A |
| 218253_s_at | LGTN | ligatin |
| 218358_at | CRELD2 | cysteine-rich with EGF-like domains 2 |
| 218418_s_at | ANKRD25 | ankyrin repeat domain 25 |
| 218429_s_at | FLJ11286 | hypothetical protein FLJ11286 |
| 218505_at | WDR59 | WD repeat domain 59 |
| 218803_at | CHFR | checkpoint with forkhead and ring finger domains |
| 218958_at | C19orf60 | chromosome 19 open reading frame 60 |
| 219045_at | RHOF | ras homolog gene family, member F (in filopodia) |
| 219196_at | SCG3 | secretogranin III |
| 219290_x_at | DAPP1 | dual adaptor of phosphotyrosine and 3-phosphoinositides |
| 219333_s_at | CAPN10 | calpain 10 |
| 219392_x_at | PRR11 | proline rich 11 |
| 219426_at | EIF2C3 | eukaryotic translation initiation factor 2C, 3 |
| 219527_at | MOSC2 | MOCO sulphurase C-terminal domain containing 2 |
| 219577_s_at | ABCA7 | ATP-binding cassette, sub-family A (ABC1), member 7 |
| 219627_at | ZNF767 | zinc finger family member 767 |
| 219774_at | CCDC93 | coiled-coil domain containing 93 |
| 219807_x_at | RAB4B | RAB4B, member RAS oncogene family |
| 219968_at | ZNF589 | zinc finger protein 589 |
| 219975_x_at | OLAH | oleoyl-ACP hydrolase |
| 220029_at | ELOVL2 | elongation of very long chain fatty acids (FEN1/Elo2, SUR4/Elo3, yeast)-like 2 |
| 220071_x_at | CEP27 | centrosomal protein 27kDa |
| 220113_x_at | POLR1B | polymerase (RNA) I polypeptide B, 128kDa |
| 220612_at | --- | Clone HQ0641 PRO0641 |
| 220642_x_at | GPR89A; LOC728932; UNQ192 | G protein-coupled receptor 89A; similar to G protein-coupled receptor 89 |
| 220694_at | DDEF1IT1 | DDEF1 intronic transcript 1 |
| 220791_x_at | SCN11A | sodium channel, voltage-gated, type XI, alpha subunit |
| 220892_s_at | PSAT1 | phosphoserine aminotransferase 1 |
| 221036_s_at | APH1B | anterior pharynx defective 1 homolog B (C. elegans) |
| 221050_s_at | GTPBP2 | GTP binding protein 2 |
| 221141_x_at | EPN1 | epsin 1 |
| 221176_x_at | WBSCR23 | Williams-Beuren syndrome chromosome region 23 |
| 221191_at | DKFZP434A0131 | DKFZp434A0131 protein |
| 221307_at | KCNIP1 | Kv channel interacting protein 1 |
| 221501_x_at | LOC339047 | hypothetical protein LOC339047 |
| 221535_at | LSG1 | large subunit GTPase 1 homolog (S. cerevisiae) |
| 221636_s_at | MOSC2 | MOCO sulphurase C-terminal domain containing 2 |
| 221739_at | C19orf10 | chromosome 19 open reading frame 10 |
| 221829_s_at | TNPO1 | transportin 1 |
| 221881_s_at | CLIC4 | chloride intracellular channel 4 |
| 221963_x_at | --- | Transcribed locus |
| 221972_s_at | SDF4 | stromal cell derived factor 4 |
| 222024_s_at | AKAP13 | A kinase (PRKA) anchor protein 13 |
| 222026_at | RBM3 | RNA binding motif (RNP1, RRM) protein 3 |
| 222104_x_at | GTF2H3 | general transcription factor IIH, polypeptide 3, 34kDa |
| 222150_s_at | LOC54103 | hypothetical protein LOC54103 |
| 222158_s_at | C1orf121 | chromosome 1 open reading frame 121 |
| 222159_at | --- | CDNA FLJ12996 fis, clone NT2RP3000235 |
| 222282_at | --- | Transcribed locus |
| 222284_at | --- | Transcribed locus |
| 222302_at | --- | --- |
| 222319_at | --- | --- |
| 222320_at | --- | Transcribed locus |
| 222366_at | --- | Transcribed locus |
| 222385_x_at | SEC61A1 | Sec61 alpha 1 subunit (S. cerevisiae) |
| 222540_s_at | RSF1 | remodeling and spacing factor 1 |
| 222760_at | ZNF703 | zinc finger protein 703 |
| 222762_x_at | LIMD1 | LIM domains containing 1 |
| 222780_s_at | BAALC | brain and acute leukemia, cytoplasmic |
| 223134_at | BBX | bobby sox homolog (Drosophila) |
| 223390_at | C9orf37 | chromosome 9 open reading frame 37 |
| 223519_at | ZAK | sterile alpha motif and leucine zipper containing kinase AZK |
| 223528_s_at | LOC731602; METT11D1 | methyltransferase 11 domain containing 1; similar to methyltransferase 11 domain containing 1 isoform 2 |
| 223534_s_at | RPS6KL1 | ribosomal protein S6 kinase-like 1 |
| 223539_s_at | LOC728492; SERF1A; SERF1B | small EDRK-rich factor 1A (telomeric); small EDRK-rich factor 1B (centromeric); similar to small EDRK-rich factor 1A, telomeric |
| 223933_at | KIF5A | kinesin family member 5A |
| 224105_x_at | --- | Clone FLB8034 PRO2158 |
| 224196_x_at | DPH5 | DPH5 homolog (S. cerevisiae) |
| 224259_at | WNT8A | wingless-type MMTV integration site family, member 8A |
| 224372_at | IQWD1; UNC5B | IQ motif and WD repeats 1; unc-5 homolog B (C. elegans) |
| 224567_x_at | MALAT1 | metastasis associated lung adenocarcinoma transcript 1 (non-coding RNA) |
| 224569_s_at | IRF2BP2 | interferon regulatory factor 2 binding protein 2 |
| 224667_x_at | C10orf104 | chromosome 10 open reading frame 104 |
| 224712_x_at | C19orf42 | chromosome 19 open reading frame 42 |
| 224727_at | LOC284361 | hematopoietic signal peptide-containing |
| 224771_at | NAV1 | neuron navigator 1 |
| 224804_s_at | C15orf17 | chromosome 15 open reading frame 17 |
| 224904_at | PDPR | pyruvate dehydrogenase phosphatase regulatory subunit |
| 224970_at | NFIA | nuclear factor I/A |
| 224991_at | CMIP | c-Maf-inducing protein |
| 225035_x_at | CXYorf1; FAM39B; FAM39DP; FLJ00038; LOC376475; LOC653635 | family with sequence similarity 39, member D pseudogene; family with sequence similarity 39, member B; CXYorf1-related protein; chromosomes X and Y open reading frame 1; similar to CXYorf1-related protein |
| 225117_at | KIAA1267 | KIAA1267 |
| 225234_at | CBL | Cas-Br-M (murine) ecotropic retroviral transforming sequence |
| 225269_s_at | RBMS1 | RNA binding motif, single stranded interacting protein 1 |
| 225298_at | PNKD | paroxysmal nonkinesiogenic dyskinesia |
| 225311_at | IVD | isovaleryl Coenzyme A dehydrogenase |
| 225529_at | CENTB5 | centaurin, beta 5 |
| 225570_at | SLC41A1 | solute carrier family 41, member 1 |
| 225758_s_at | TUBGCP6 | tubulin, gamma complex associated protein 6 |
| 225866_at | BXDC1 | brix domain containing 1 |
| 225906_at | --- | CDNA FLJ38264 fis, clone FCBBF3001657 |
| 225973_at | TAP2 | transporter 2, ATP-binding cassette, sub-family B (MDR/TAP) |
| 225995_x_at | FAM39B | family with sequence similarity 39, member B |
| 226144_at | REXO1 | REX1, RNA exonuclease 1 homolog (S. cerevisiae) |
| 226153_s_at | CNOT6L | CCR4-NOT transcription complex, subunit 6-like |
| 226179_at | SLC25A37 | solute carrier family 25, member 37 |
| 226200_at | VARS2 | valyl-tRNA synthetase 2, mitochondrial (putative) |
| 226252_at | --- | CDNA FLJ34585 fis, clone KIDNE2008758 |
| 226354_at | LACTB | lactamase, beta |
| 226372_at | CHST11 | Carbohydrate (chondroitin 4) sulfotransferase 11 |
| 226554_at | ZBTB7A | zinc finger and BTB domain containing 7A |
| 226620_x_at | DAZAP1 | DAZ associated protein 1 |
| 226848_at | --- | CDNA FLJ39306 fis, clone OCBBF2013123 |
| 226876_at | FAM101B | family with sequence similarity 101, member B |
| 227039_at | AKAP13 | A kinase (PRKA) anchor protein 13 |
| 227082_at | --- | MRNA; cDNA DKFZp586K1922 (from clone DKFZp586K1922) |
| 227168_at | MIAT | myocardial infarction associated transcript (non-protein coding) |
| 227208_at | CCDC84 | coiled-coil domain containing 84 |
| 227286_at | CCDC95 | coiled-coil domain containing 95 |
| 227347_x_at | HES4 | hairy and enhancer of split 4 (Drosophila) |
| 227417_at | MOSC2 | MOCO sulphurase C-terminal domain containing 2 |
| 227431_at | --- | CDNA clone IMAGE:4791585 |
| 227484_at | --- | CDNA FLJ41690 fis, clone HCASM2009405 |
| 227640_s_at | LOC441212; RP9 | retinitis pigmentosa 9 (autosomal dominant); retinitis pigmentosa 9 pseudogene |
| 227651_at | BTBD14B | BTB (POZ) domain containing 14B |
| 227677_at | JAK3 | Janus kinase 3 (a protein tyrosine kinase, leukocyte) |
| 227727_at | MRGPRF | MAS-related GPR, member F |
| 227923_at | SHANK3 | SH3 and multiple ankyrin repeat domains 3 |
| 227931_at | --- | MRNA; cDNA DKFZp686D22106 (from clone DKFZp686D22106) |
| 227948_at | FGD4 | FYVE, RhoGEF and PH domain containing 4 |
| 228070_at | --- | CDNA FLJ34250 fis, clone FCBBF4000529 |
| 228155_at | C10orf58 | chromosome 10 open reading frame 58 |
| 228224_at | PRELP | proline/arginine-rich end leucine-rich repeat protein |
| 229220_x_at | NOM1 | nucleolar protein with MIF4G domain 1 |
| 229265_at | ATN1 | Atrophin 1 |
| 229272_at | FNBP4 | formin binding protein 4 |
| 229351_at | --- | CDNA FLJ13620 fis, clone PLACE1010947 |
| 229467_at | PCBP2 | Poly(rC) binding protein 2 |
| 229574_at | TRA2A | transformer-2 alpha |
| 229654_at | ZNF44 | Zinc finger protein 44 |
| 229686_at | P2RY8 | purinergic receptor P2Y, G-protein coupled, 8 |
| 229851_s_at | C11orf54 | chromosome 11 open reading frame 54 |
| 229858_at | --- | CDNA FLJ12024 fis, clone HEMBB1001797 |
| 229943_at | TRIM13 | tripartite motif-containing 13 |
| 230014_at | --- | Transcribed locus |
| 230200_at | NSUN6 | NOL1/NOP2/Sun domain family, member 6 |
| 230440_at | ZNF469 | zinc finger protein 469 |
| 230528_s_at | MGC2752 | hypothetical protein MGC2752 |
| 230599_at | --- | Transcribed locus |
| 230630_at | --- | Transcribed locus |
| 230733_at | --- | Transcribed locus |
| 230779_at | TNRC6B | trinucleotide repeat containing 6B |
| 230820_at | --- | Transcribed locus |
| 230843_at | --- | Transcribed locus |
| 230941_at | LOC728537; LOC730797 | hypothetical protein LOC728537; hypothetical protein LOC730797 |
| 231109_at | --- | CDNA FLJ38468 fis, clone FEBRA2021864 |
| 231116_at | --- | Transcribed locus |
| 231164_at | LOC440331 | hypothetical gene supported by AK095200; BC042853 |
| 231212_x_at | --- | --- |
| 231238_at | --- | Transcribed locus |
| 231240_at | DIO2 | deiodinase, iodothyronine, type II |
| 231495_at | --- | Transcribed locus |
| 231696_x_at | --- | Transcribed locus |
| 231828_at | LOC253039 | Hypothetical protein LOC253039 |
| 231829_at | VISA | virus-induced signaling adapter |
| 231992_x_at | --- | CDNA clone IMAGE:4722553 |
| 232015_at | FAM59B | family with sequence similarity 59, member B |
| 232096_x_at | --- | CDNA: FLJ22140 fis, clone HEP20977 |
| 232125_at | --- | CDNA FLJ34585 fis, clone KIDNE2008758 |
| 232169_x_at | NDUFS8 | NADH dehydrogenase (ubiquinone) Fe-S protein 8, 23kDa (NADH-coenzyme Q reductase) |
| 232175_at | ARF1 | ADP-ribosylation factor 1 |
| 232215_x_at | PRR11 | proline rich 11 |
| 232216_at | YME1L1 | YME1-like 1 (S. cerevisiae) |
| 232225_at | --- | CDNA FLJ11764 fis, clone HEMBA1005685 |
| 232264_at | --- | CDNA FLJ12142 fis, clone MAMMA1000356 |
| 232347_x_at | --- | CDNA FLJ11379 fis, clone HEMBA1000469 |
| 232392_at | SFRS3 | Splicing factor, arginine/serine-rich 3 |
| 232396_at | --- | MRNA full length insert cDNA clone EUROIMAGE 113222 |
| 232408_at | ZFYVE28 | zinc finger, FYVE domain containing 28 |
| 232416_at | BRUNOL5 | bruno-like 5, RNA binding protein (Drosophila) |
| 232420_x_at | LOC286260 | hypothetical protein LOC286260 |
| 232455_x_at | LOC340085 | hypothetical protein LOC340085 |
| 232472_at | --- | CDNA FLJ12399 fis, clone MAMMA1002780 |
| 232516_x_at | YY1AP1 | YY1 associated protein 1 |
| 232537_x_at | MARK3 | MAP/microtubule affinity-regulating kinase 3 |
| 232541_at | --- | CDNA FLJ20099 fis, clone COL04544 |
| 232554_at | LRRC56 | leucine rich repeat containing 56 |
| 232614_at | --- | CDNA FLJ12049 fis, clone HEMBB1001996 |
| 232615_at | --- | CDNA: FLJ22765 fis, clone KAIA1180 |
| 232653_at | --- | CDNA FLJ14044 fis, clone HEMBA1006124 |
| 232663_s_at | LOC390595 | similar to ubiquitin-associated protein 1 (predicted) |
| 232665_x_at | --- | --- |
| 232685_at | --- | CDNA: FLJ21564 fis, clone COL06452 |
| 232691_at | SFXN5 | Sideroflexin 5 |
| 232693_s_at | FBXO16; ZNF395 | zinc finger protein 395; F-box protein 16 |
| 232753_at | ZNF346 | Zinc finger protein 346 |
| 232882_at | --- | CDNA FLJ12289 fis, clone MAMMA1001788 |
| 232935_at | --- | Primary neuroblastoma cDNA, clone:Nbla03614, full insert sequence |
| 232940_s_at | MLL3 | myeloid/lymphoid or mixed-lineage leukemia 3 |
| 232957_x_at | --- | CDNA FLJ13017 fis, clone NT2RP3000628 |
| 233014_at | --- | CDNA FLJ12918 fis, clone NT2RP2004580 |
| 233017_x_at | --- | CDNA FLJ12326 fis, clone MAMMA1002132 |
| 233025_at | PDZD2 | PDZ domain containing 2 |
| 233041_x_at | --- | CDNA: FLJ21356 fis, clone COL02831 |
| 233130_at | --- | CDNA FLJ12202 fis, clone MAMMA1000908 |
| 233168_s_at | RP3-402G11.5 | selenoprotein O |
| 233226_at | PTPN9 | Protein tyrosine phosphatase, non-receptor type 9 |
| 233265_at | --- | CDNA FLJ12203 fis, clone MAMMA1000914 |
| 233306_at | --- | CDNA FLJ11447 fis, clone HEMBA1001383 |
| 233313_at | --- | CDNA FLJ14302 fis, clone PLACE2000003 |
| 233315_at | --- | CDNA: FLJ21294 fis, clone COL01981 |
| 233319_x_at | --- | CDNA FLJ13845 fis, clone THYRO1000815 |
| 233321_x_at | LOC90834 | hypothetical protein BC001742 |
| 233417_at | --- | CDNA FLJ11625 fis, clone HEMBA1004200 |
| 233427_x_at | --- | CDNA FLJ13808 fis, clone THYRO1000253 |
| 233442_at | --- | CDNA FLJ12196 fis, clone MAMMA1000867 |
| 233449_at | --- | CDNA FLJ11377 fis, clone HEMBA1000442 |
| 233596_at | --- | Clone FLB2543 |
| 233605_x_at | HNRPM | heterogeneous nuclear ribonucleoprotein M |
| 233608_at | --- | CDNA FLJ11929 fis, clone HEMBB1000434 |
| 233622_x_at | --- | MRNA; cDNA DKFZp761A219 (from clone DKFZp761A219) |
| 233664_at | --- | CDNA: FLJ22803 fis, clone KAIA2685 |
| 233702_x_at | --- | CDNA: FLJ20946 fis, clone ADSE01819 |
| 233851_s_at | TOR3A | torsin family 3, member A |
| 233873_x_at | PAPD1 | PAP associated domain containing 1 |
| 233877_at | --- | CDNA FLJ20770 fis, clone COL06509 |
| 233901_at | --- | MRNA full length insert cDNA clone EUROIMAGE 163507 |
| 234033_at | --- | Clone IMAGE:110218 mRNA sequence |
| 234126_at | --- | MRNA; cDNA DKFZp761M1112 (from clone DKFZp761M1112) |
| 234127_at | --- | CDNA FLJ13320 fis, clone OVARC1001611 |
| 234135_x_at | --- | CDNA FLJ11590 fis, clone HEMBA1003758 |
| 234159_at | --- | CDNA: FLJ21529 fis, clone COL05981 |
| 234294_x_at | GATAD2A | GATA zinc finger domain containing 2A |
| 234307_s_at | KIF26A | kinesin family member 26A |
| 234314_at | C20orf74 | chromosome 20 open reading frame 74 |
| 234382_x_at | --- | --- |
| 234491_s_at | SAV1 | salvador homolog 1 (Drosophila) |
| 234501_x_at | --- | MRNA; cDNA DKFZp586M151 (from clone DKFZp586M151) |
| 234762_x_at | NLN | Neurolysin (metallopeptidase M3 family) |
| 234981_x_at | CMBL | carboxymethylenebutenolidase homolog (Pseudomonas) |
| 234989_at | TncRNA | trophoblast-derived noncoding RNA |
| 235041_at | GOSR2 | golgi SNAP receptor complex member 2 |
| 235081_x_at | TRIM65 | tripartite motif-containing 65 |
| 235084_x_at | --- | Transcribed locus |
| 235308_at | ZBTB20 | zinc finger and BTB domain containing 20 |
| 235508_at | PML | promyelocytic leukemia |
| 235538_at | --- | CDNA FLJ30718 fis, clone FCBBF2001675 |
| 235652_at | --- | CDNA FLJ37623 fis, clone BRCOC2014013 |
| 235660_at | --- | MRNA; cDNA DKFZp667E0114 (from clone DKFZp667E0114) |
| 235756_at | --- | CDNA FLJ26187 fis, clone ADG04782 |
| 235803_at | --- | Transcribed locus |
| 235847_at | --- | Transcribed locus |
| 235875_at | --- | Transcribed locus |
| 235902_at | --- | CDNA FLJ42963 fis, clone BRSTN2012380 |
| 235985_at | --- | Transcribed locus |
| 235990_at | --- | CDNA FLJ38836 fis, clone MESAN2002519, weakly similar to Mus musculus cell cycle checkpoint control protein Mrad9 gene |
| 235999_at | --- | Transcribed locus |
| 236041_at | --- | CDNA FLJ33236 fis, clone ASTRO2002571 |
| 236060_at | --- | Transcribed locus |
| 236229_at | --- | Transcribed locus |
| 236327_at | --- | --- |
| 236558_at | --- | --- |
| 236593_at | --- | Transcribed locus, weakly similar to XP_341471.3 similar to WD repeat domain 17 [Rattus norvegicus] |
| 236617_at | --- | Transcribed locus |
| 236679_x_at | --- | --- |
| 236766_at | C8orf38 | Chromosome 8 open reading frame 38 |
| 236841_at | FAM39DP | Family with sequence similarity 39, member D pseudogene |
| 236923_x_at | --- | --- |
| 236946_at | GPR75 | G protein-coupled receptor 75 |
| 236966_at | ARMC8 | armadillo repeat containing 8 |
| 237035_at | --- | Transcribed locus |
| 237108_x_at | FLJ42875 | FLJ42875 protein |
| 237118_at | --- | --- |
| 237383_at | --- | Transcribed locus |
| 237398_at | --- | Transcribed locus |
| 237475_x_at | SEPP1 | Selenoprotein P, plasma, 1 |
| 237491_at | --- | --- |
| 237586_at | --- | --- |
| 237733_at | --- | --- |
| 237864_at | --- | CDNA FLJ26101 fis, clone SLV05922 |
| 238058_at | --- | --- |
| 238152_at | MGC3032 | hypothetical protein MGC3032 |
| 238430_x_at | SLFN5 | schlafen family member 5 |
| 238447_at | RBMS3 | RNA binding motif, single stranded interacting protein |
| 238642_at | --- | --- |
| 238672_at | --- | Transcribed locus |
| 238712_at | --- | Transcribed locus |
| 238743_at | --- | Full-length cDNA clone CS0DK002YF13 of HeLa cells Cot 25-normalized of Homo sapiens (human) |
| 238761_at | --- | Transcribed locus |
| 238863_x_at | --- | Transcribed locus |
| 238884_at | --- | Transcribed locus |
| 238964_at | --- | Transcribed locus |
| 239058_at | --- | Transcribed locus |
| 239091_at | --- | Transcribed locus |
| 239167_at | --- | Transcribed locus |
| 239171_at | --- | --- |
| 239190_at | VRK3 | vaccinia related kinase 3 |
| 239296_at | --- | Transcribed locus |
| 239361_at | --- | Transcribed locus |
| 239385_at | TFG | TRK-fused gene |
| 239448_at | --- | Transcribed locus |
| 239629_at | CFLAR | CASP8 and FADD-like apoptosis regulator |
| 239661_at | --- | Transcribed locus |
| 239748_x_at | OCIAD1 | OCIA domain containing 1 |
| 239753_at | LOC441383 | hypothetical gene supported by AF086559; BC065734 |
| 239804_at | --- | Transcribed locus |
| 239851_at | --- | --- |
| 239856_at | --- | Transcribed locus, strongly similar to XP_001175123.1 hypothetical protein [Pan troglodytes] |
| 239956_at | --- | Transcribed locus |
| 240125_at | --- | Transcribed locus |
| 240138_at | --- | Transcribed locus |
| 240139_at | --- | Transcribed locus |
| 240146_at | --- | --- |
| 240165_at | --- | Transcribed locus |
| 240168_at | --- | --- |
| 240174_at | --- | Transcribed locus |
| 240205_x_at | --- | --- |
| 240279_at | --- | --- |
| 240544_at | --- | Transcribed locus |
| 240651_at | --- | Transcribed locus |
| 240665_at | --- | --- |
| 240666_at | --- | Transcribed locus |
| 240773_at | --- | Transcribed locus |
| 240798_at | --- | --- |
| 240870_at | --- | --- |
| 240971_x_at | --- | --- |
| 241223_x_at | --- | Transcribed locus, weakly similar to NP_001013658.1 protein LOC387873 [Homo sapiens] |
| 241303_x_at | --- | --- |
| 241336_at | --- | --- |
| 241347_at | KIAA1618 | KIAA1618 |
| 241445_at | --- | Transcribed locus |
| 241464_s_at | --- | Transcribed locus |
| 241542_at | --- | --- |
| 241585_at | LRRC4C | leucine rich repeat containing 4C |
| 241790_at | --- | Transcribed locus |
| 241797_at | --- | --- |
| 241818_at | --- | Transcribed locus |
| 241843_at | SNORA28 | small nucleolar RNA, H/ACA box 28 |
| 241987_x_at | MGC39715 | hypothetical protein MGC39715 |
| 242022_at | --- | --- |
| 242077_x_at | C6orf150 | chromosome 6 open reading frame 150 |
| 242099_at | --- | --- |
| 242106_at | --- | Transcribed locus |
| 242121_at | RNF12 | Ring finger protein 12 |
| 242167_at | --- | --- |
| 242171_at | --- | --- |
| 242188_at | --- | --- |
| 242232_at | --- | --- |
| 242235_x_at | NRD1 | Nardilysin (N-arginine dibasic convertase) |
| 242261_at | --- | --- |
| 242268_at | CUGBP2 | CUG triplet repeat, RNA binding protein 2 |
| 242280_x_at | CPEB4 | cytoplasmic polyadenylation element binding protein 4 |
| 242303_at | --- | Transcribed locus |
| 242320_at | --- | Homo sapiens, clone IMAGE:4769230, mRNA |
| 242364_x_at | --- | CDNA clone IMAGE:5286005 |
| 242377_x_at | THUMPD3 | THUMP domain containing 3 |
| 242405_at | --- | Transcribed locus |
| 242407_at | --- | --- |
| 242416_at | --- | --- |
| 242431_at | --- | --- |
| 242443_at | EML5 | Echinoderm microtubule associated protein like 5 |
| 242461_at | --- | --- |
| 242471_at | --- | Clone HLS_IMAGE_238756 mRNA sequence |
| 242472_x_at | FNBP4 | Formin binding protein 4 |
| 242480_at | --- | Transcribed locus |
| 242551_at | --- | --- |
| 242558_at | --- | CDNA FLJ45490 fis, clone BRTHA2005831 |
| 242578_x_at | SLC22A3 | Solute carrier family 22 (extraneuronal monoamine transporter), member 3 |
| 242579_at | --- | Transcribed locus |
| 242611_at | --- | Transcribed locus |
| 242622_x_at | PTEN | Phosphatase and tensin homolog (mutated in multiple advanced cancers 1) |
| 242645_at | --- | Full length insert cDNA clone YR92A01 |
| 242664_at | --- | --- |
| 242670_at | LGI4 | leucine-rich repeat LGI family, member 4 |
| 242671_at | --- | --- |
| 242736_at | --- | --- |
| 242846_at | --- | Transcribed locus |
| 242859_at | --- | --- |
| 242865_at | --- | --- |
| 242872_at | CIT | citron (rho-interacting, serine/threonine kinase 21) |
| 242889_x_at | LOC645431 | hypothetical protein LOC645431 |
| 242903_at | IFNGR1 | interferon gamma receptor 1 |
| 243006_at | --- | CDNA FLJ30333 fis, clone BRACE2007262 |
| 243158_at | --- | --- |
| 243169_at | --- | --- |
| 243218_at | --- | Transcribed locus |
| 243291_at | --- | Transcribed locus |
| 243295_at | RBM27 | RNA binding motif protein 27 |
| 243365_s_at | AUTS2 | autism susceptibility candidate 2 |
| 243431_at | --- | Transcribed locus |
| 243442_x_at | --- | Transcribed locus |
| 243546_at | --- | Transcribed locus |
| 243586_at | --- | Transcribed locus |
| 243612_at | NSD1 | Nuclear receptor binding SET domain protein 1 |
| 243618_s_at | LOC152485 | Hypothetical protein LOC152485 |
| 243640_x_at | --- | --- |
| 243648_at | --- | --- |
| 243826_at | --- | Transcribed locus |
| 243929_at | --- | --- |
| 243963_at | SDCCAG8 | Serologically defined colon cancer antigen 8 |
| 243964_at | --- | Transcribed locus |
| 243997_x_at | --- | Transcribed locus |
| 244062_at | DAAM1 | dishevelled associated activator of morphogenesis 1 |
| 244093_at | --- | --- |
| 244197_x_at | --- | --- |
| 244217_at | --- | Transcribed locus |
| 244241_x_at | --- | Transcribed locus |
| 244310_at | --- | Full length insert cDNA clone YU07D01 |
| 244345_at | CADM1 | cell adhesion molecule 1 |
| 244358_at | --- | --- |
| 244373_at | --- | --- |
| 244433_at | --- | --- |
| 244457_at | --- | Transcribed locus |
| 244459_at | --- | Transcribed locus |
| 244480_at | --- | Transcribed locus |
| 244535_at | --- | Transcribed locus |
| 244579_at | --- | Transcribed locus |
| 244605_at | --- | --- |
| 244646_at | --- | Transcribed locus |
| 244669_at | SNHG5; SNORD50A; SNORD50B | small nucleolar RNA, C/D box 50A; small nucleolar RNA host gene (non-protein coding) 5; small nucleolar RNA, C/D box 50B |
| 244697_at | --- | --- |
| 244726_at | --- | Transcribed locus |
| 244753_at | --- | Transcribed locus |
| 244826_at | --- | Transcribed locus |
| 244868_at | --- | --- |
| 33132_at | CPSF1 | cleavage and polyadenylation specific factor 1, 160kDa |
| 34697_at | LRP6 | low density lipoprotein receptor-related protein 6 |
| 36019_at | STK19 | serine/threonine kinase 19 |
| 36936_at | TSTA3 | tissue specific transplantation antigen P35B |
| 37278_at | TAZ | tafazzin (cardiomyopathy, dilated 3A (X-linked); endocardial fibroelastosis 2; Barth syndrome) |
| 38157_at | DOM3Z | dom-3 homolog Z (C. elegans) |
| 39854_r_at | PNPLA2 | patatin-like phospholipase domain containing 2 |
| 40016_g_at | MAST4 | microtubule associated serine/threonine kinase family member 4 |
| 40562_at | GNA11 | guanine nucleotide binding protein (G protein), alpha 11 (Gq class) |
| 41160_at | MBD3 | methyl-CpG binding domain protein 3 |
| 41512_at | --- | Transcribed locus |
| 41644_at | SASH1 | SAM and SH3 domain containing 1 |
| 46167_at | C1orf175; TTC4 | tetratricopeptide repeat domain 4; chromosome 1 open reading frame 175 |
| 52078_at | C1orf160 | chromosome 1 open reading frame 160 |
| 90610_at | LRCH4 | leucine-rich repeats and calponin homology (CH) domain containing 4 |

Table S2. Compiled table of all 790 probes detected to be significantly different from background and overlapping in at least two different pathologically defined groups. When possible probes are given in official gene symbol names. Gene symbol: official genbank gene symbol. Gene Title: official genbank gene name. ---: Unknown
