# Supplementary material for: Comprehensive mRNA Expression Profiling Distinguishes Tauopathies and Identifies Shared Molecular Pathways
Source: PLoS One. 2009 Aug 28;4(8):e6826. doi: 10.1371/journal.pone.0006826 (PMC2729393; doi:10.1371/journal.pone.0006826)
Supplement: Table S3 — Compiled table of all 166 probes detected to be significantly different from background and overlapping in at least two different pathologically defined groups. When possible probes are given in official gene symbol. Gene Title: official genbank gene name. ---: Unknown (0.20 MB DOC) [file pone.0006826.s003.doc]

| **Multiple overlapping significantly altered probes / genes** | | |
| --- | --- | --- |
| Probe Set ID | Gene Symbol | Gene Title |
| 1552301_a_at | CORO6 | coronin 6 |
| 1552536_at | VTI1A | vesicle transport through interaction with t-SNAREs homolog 1A (yeast) |
| 1553565_s_at | DDAH1 | dimethylarginine dimethylaminohydrolase 1 |
| 1553613_s_at | FOXC1 | forkhead box C1 |
| 1553995_a_at | NT5E | 5'-nucleotidase, ecto (CD73) |
| 1555014_x_at | --- | OK/SW-cl.92 |
| 1556000_s_at | BTBD7 | BTB (POZ) domain containing 7 |
| 1556606_at | NAV2 | neuron navigator 2 |
| 1558009_at | SLC1A2 | solute carrier family 1 (glial high affinity glutamate transporter), member 2 |
| 1558010_s_at | SLC1A2 | solute carrier family 1 (glial high affinity glutamate transporter), member 2 |
| 1558041_a_at | LOC653319 | hypothetical protein LOC653319 |
| 1558783_at | --- | CDNA: FLJ21152 fis, clone CAS09594 |
| 1559156_at | --- | MRNA; cDNA DKFZp686B1142 (from clone DKFZp686B1142) |
| 1559375_s_at | --- | Full length insert cDNA clone YI45C08 |
| 1559436_x_at | --- | MRNA; cDNA DKFZp313M2114 (from clone DKFZp313M2114) |
| 1559965_at | --- | CDNA clone IMAGE:4811567 |
| 1561167_at | --- | Full length insert cDNA clone YA75A09 |
| 1561195_at | --- | MRNA; cDNA DKFZp686A22111 (from clone DKFZp686A22111) |
| 1561346_at | --- | CDNA FLJ32691 fis, clone TESTI2000221 |
| 1562063_x_at | KIAA1245; LOC728895; NBPF1; NBPF10; NBPF11; NBPF20; NBPF3; NBPF8; NBPF9; XXyac-YX155B6.1 | neuroblastoma breakpoint family, member 1; neuroblastoma breakpoint family, member 3; KIAA1245; neuroblastoma breakpoint family, member 11; neuroblastoma breakpoint family, member 20; neuroblastoma breakpoint family, member 9; neuroblastoma breakpoint family, member 10; neuroblastoma breakpoint family, member 8; hypothetical protein LOC728895; CLIP-190-like |
| 1565692_at | --- | CDNA FLJ40647 fis, clone THYMU2017522 |
| 1566887_x_at | --- | Multiple myeloma susceptibility mRNA sequence |
| 1569477_at | --- | Homo sapiens, clone IMAGE:4291396, mRNA |
| 1570414_x_at | FLJ13197 | hypothetical FLJ13197 |
| 200778_s_at | Sep-02 | septin 2 |
| 201135_at | ECHS1 | enoyl Coenzyme A hydratase, short chain, 1, mitochondrial |
| 201185_at | HTRA1 | HtrA serine peptidase 1 |
| 202040_s_at | JARID1A | jumonji, AT rich interactive domain 1A |
| 202935_s_at | SOX9 | SRY (sex determining region Y)-box 9 (campomelic dysplasia, autosomal sex-reversal) |
| 203628_at | IGF1R | insulin-like growth factor 1 receptor |
| 203668_at | MAN2C1 | mannosidase, alpha, class 2C, member 1 |
| 203802_x_at | NSUN5 | NOL1/NOP2/Sun domain family, member 5 |
| 204257_at | FADS3 | fatty acid desaturase 3 |
| 204538_x_at | LOC339047; LOC642778; LOC642799; NPIP | nuclear pore complex interacting protein; hypothetical protein LOC339047; similar to nuclear pore complex interacting protein |
| 204650_s_at | APBB3 | amyloid beta (A4) precursor protein-binding, family B, member 3 |
| 205130_at | RAGE | renal tumor antigen |
| 205344_at | CSPG5 | chondroitin sulfate proteoglycan 5 (neuroglycan C) |
| 205887_x_at | MSH3 | mutS homolog 3 (E. coli) |
| 206056_x_at | SPN | sialophorin (leukosialin, CD43) |
| 206527_at | ABAT | 4-aminobutyrate aminotransferase |
| 206548_at | FLJ23556 | hypothetical protein FLJ23556 |
| 206565_x_at | SMA3 | SMA3 |
| 206792_x_at | PDE4C | phosphodiesterase 4C, cAMP-specific (phosphodiesterase E1 dunce homolog, Drosophila) |
| 206950_at | SCN9A | sodium channel, voltage-gated, type IX, alpha subunit |
| 207660_at | DMD | dystrophin (muscular dystrophy, Duchenne and Becker types) |
| 208686_s_at | BRD2 | bromodomain containing 2 |
| 209708_at | MOXD1 | monooxygenase, DBH-like 1 |
| 210407_at | PPM1A | protein phosphatase 1A (formerly 2C), magnesium-dependent, alpha isoform |
| 210528_at | MR1 | major histocompatibility complex, class I-related |
| 211876_x_at | PCDHGA10; PCDHGA11; PCDHGA12; PCDHGA3; PCDHGA5; PCDHGA6 | protocadherin gamma subfamily A, 12; protocadherin gamma subfamily A, 11; protocadherin gamma subfamily A, 10; protocadherin gamma subfamily A, 6; protocadherin gamma subfamily A, 5; protocadherin gamma subfamily A, 3 |
| 212079_s_at | MLL | myeloid/lymphoid or mixed-lineage leukemia (trithorax homolog, Drosophila) |
| 212228_s_at | COQ9 | coenzyme Q9 homolog (S. cerevisiae) |
| 213143_at | LOC257407 | hypothetical protein LOC257407 |
| 213652_at | PCSK5 | Proprotein convertase subtilisin/kexin type 5 |
| 214004_s_at | VGLL4 | vestigial like 4 (Drosophila) |
| 214707_x_at | ALMS1 | Alstrom syndrome 1 |
| 214882_s_at | SFRS2 | splicing factor, arginine/serine-rich 2 |
| 215067_x_at | PRDX2 | peroxiredoxin 2 |
| 215372_x_at | --- | CDNA FLJ12002 fis, clone HEMBB1001536 |
| 215383_x_at | SPG21 | spastic paraplegia 21 (autosomal recessive, Mast syndrome) |
| 215435_at | --- | CDNA FLJ11921 fis, clone HEMBB1000318 |
| 215600_x_at | FBXW12 | F-box and WD repeat domain containing 12 |
| 215615_x_at | --- | CDNA FLJ14152 fis, clone MAMMA1003089 |
| 215683_at | --- | Clone 24803 mRNA sequence |
| 215982_s_at | DOM3Z | dom-3 homolog Z (C. elegans) |
| 216187_x_at | --- | Alu repeat (LNX1) mRNA sequence |
| 216189_at | --- | Homo sapiens, clone IMAGE:3344506 |
| 216532_x_at | LOC643450; LOC728344 | similar to Thioredoxin-like protein 2 (PKC-interacting cousin of thioredoxin) (PKC-theta-interacting protein) (PKCq-interacting protein) |
| 216958_s_at | IVD | isovaleryl Coenzyme A dehydrogenase |
| 217550_at | ATF6 | Activating transcription factor 6 |
| 217643_x_at | --- | --- |
| 217679_x_at | --- | --- |
| 217713_x_at | --- | --- |
| 217715_x_at | --- | --- |
| 217810_x_at | LARS | leucyl-tRNA synthetase |
| 218803_at | CHFR | checkpoint with forkhead and ring finger domains |
| 218958_at | C19orf60 | chromosome 19 open reading frame 60 |
| 219392_x_at | PRR11 | proline rich 11 |
| 219975_x_at | OLAH | oleoyl-ACP hydrolase |
| 220071_x_at | CEP27 | centrosomal protein 27kDa |
| 220694_at | DDEF1IT1 | DDEF1 intronic transcript 1 |
| 220791_x_at | SCN11A | sodium channel, voltage-gated, type XI, alpha subunit |
| 221307_at | KCNIP1 | Kv channel interacting protein 1 |
| 221501_x_at | LOC339047 | hypothetical protein LOC339047 |
| 221636_s_at | MOSC2 | MOCO sulphurase C-terminal domain containing 2 |
| 221829_s_at | TNPO1 | transportin 1 |
| 222026_at | RBM3 | RNA binding motif (RNP1, RRM) protein 3 |
| 222104_x_at | GTF2H3 | general transcription factor IIH, polypeptide 3, 34kDa |
| 222282_at | --- | Transcribed locus |
| 222366_at | --- | Transcribed locus |
| 222762_x_at | LIMD1 | LIM domains containing 1 |
| 223528_s_at | LOC731602; METT11D1 | methyltransferase 11 domain containing 1; similar to methyltransferase 11 domain containing 1 isoform 2 |
| 223534_s_at | RPS6KL1 | ribosomal protein S6 kinase-like 1 |
| 224105_x_at | --- | Clone FLB8034 PRO2158 |
| 224771_at | NAV1 | neuron navigator 1 |
| 225035_x_at | CXYorf1; FAM39B; FAM39DP; FLJ00038; LOC376475; LOC653635 | family with sequence similarity 39, member D pseudogene; family with sequence similarity 39, member B; CXYorf1-related protein; chromosomes X and Y open reading frame 1; similar to CXYorf1-related protein |
| 225234_at | CBL | Cas-Br-M (murine) ecotropic retroviral transforming sequence |
| 225311_at | IVD | isovaleryl Coenzyme A dehydrogenase |
| 225529_at | CENTB5 | centaurin, beta 5 |
| 225995_x_at | FAM39B | family with sequence similarity 39, member B |
| 226620_x_at | DAZAP1 | DAZ associated protein 1 |
| 229467_at | PCBP2 | Poly(rC) binding protein 2 |
| 229943_at | TRIM13 | tripartite motif-containing 13 |
| 231109_at | --- | CDNA FLJ38468 fis, clone FEBRA2021864 |
| 232096_x_at | --- | CDNA: FLJ22140 fis, clone HEP20977 |
| 232215_x_at | PRR11 | proline rich 11 |
| 232347_x_at | --- | CDNA FLJ11379 fis, clone HEMBA1000469 |
| 232420_x_at | LOC286260 | hypothetical protein LOC286260 |
| 232541_at | --- | CDNA FLJ20099 fis, clone COL04544 |
| 232663_s_at | LOC390595 | similar to ubiquitin-associated protein 1 (predicted) |
| 232665_x_at | --- | --- |
| 232882_at | --- | CDNA FLJ12289 fis, clone MAMMA1001788 |
| 232935_at | --- | Primary neuroblastoma cDNA, clone:Nbla03614, full insert sequence |
| 232957_x_at | --- | CDNA FLJ13017 fis, clone NT2RP3000628 |
| 233017_x_at | --- | CDNA FLJ12326 fis, clone MAMMA1002132 |
| 233319_x_at | --- | CDNA FLJ13845 fis, clone THYRO1000815 |
| 233321_x_at | LOC90834 | hypothetical protein BC001742 |
| 233449_at | --- | CDNA FLJ11377 fis, clone HEMBA1000442 |
| 233622_x_at | --- | MRNA; cDNA DKFZp761A219 (from clone DKFZp761A219) |
| 233702_x_at | --- | CDNA: FLJ20946 fis, clone ADSE01819 |
| 233901_at | --- | MRNA full length insert cDNA clone EUROIMAGE 163507 |
| 234382_x_at | --- | --- |
| 234981_x_at | CMBL | carboxymethylenebutenolidase homolog (Pseudomonas) |
| 235081_x_at | TRIM65 | tripartite motif-containing 65 |
| 235084_x_at | --- | Transcribed locus |
| 235538_at | --- | CDNA FLJ30718 fis, clone FCBBF2001675 |
| 236617_at | --- | Transcribed locus |
| 236923_x_at | --- | --- |
| 237108_x_at | FLJ42875 | FLJ42875 protein |
| 237475_x_at | SEPP1 | Selenoprotein P, plasma, 1 |
| 238743_at | --- | Full-length cDNA clone CS0DK002YF13 of HeLa cells Cot 25-normalized of Homo sapiens (human) |
| 238884_at | --- | Transcribed locus |
| 239167_at | --- | Transcribed locus |
| 239661_at | --- | Transcribed locus |
| 239748_x_at | OCIAD1 | OCIA domain containing 1 |
| 240125_at | --- | Transcribed locus |
| 240174_at | --- | Transcribed locus |
| 240665_at | --- | --- |
| 241303_x_at | --- | --- |
| 241797_at | --- | --- |
| 241818_at | --- | Transcribed locus |
| 242077_x_at | C6orf150 | chromosome 6 open reading frame 150 |
| 242106_at | --- | Transcribed locus |
| 242235_x_at | NRD1 | Nardilysin (N-arginine dibasic convertase) |
| 242280_x_at | CPEB4 | cytoplasmic polyadenylation element binding protein 4 |
| 242320_at | --- | Homo sapiens, clone IMAGE:4769230, mRNA |
| 242364_x_at | --- | CDNA clone IMAGE:5286005 |
| 242377_x_at | THUMPD3 | THUMP domain containing 3 |
| 242405_at | --- | Transcribed locus |
| 242611_at | --- | Transcribed locus |
| 242664_at | --- | --- |
| 242889_x_at | LOC645431 | hypothetical protein LOC645431 |
| 243158_at | --- | --- |
| 243291_at | --- | Transcribed locus |
| 243365_s_at | AUTS2 | autism susceptibility candidate 2 |
| 243431_at | --- | Transcribed locus |
| 243442_x_at | --- | Transcribed locus |
| 243640_x_at | --- | --- |
| 244345_at | CADM1 | cell adhesion molecule 1 |
| 244358_at | --- | --- |
| 244457_at | --- | Transcribed locus |
| 244535_at | --- | Transcribed locus |
| 244697_at | --- | --- |
| 36936_at | TSTA3 | tissue specific transplantation antigen P35B |
| 37278_at | TAZ | tafazzin (cardiomyopathy, dilated 3A (X-linked); endocardial fibroelastosis 2; Barth syndrome) |
| 41644_at | SASH1 | SAM and SH3 domain containing 1 |

Table S3. Compiled table of all 166 probes detected to be significantly different from background and overlapping in at least two different pathologically defined groups. When possible probes are given in official gene symbol. Gene Title: official genbank gene name. ---: Unknown
