# Supplementary material for: Comprehensive mRNA Expression Profiling Distinguishes Tauopathies and Identifies Shared Molecular Pathways
Source: PLoS One. 2009 Aug 28;4(8):e6826. doi: 10.1371/journal.pone.0006826 (PMC2729393; doi:10.1371/journal.pone.0006826)
Supplement: Table S4 — Overview of detected probes, signal strengths, background and noise levels. (0.17 MB DOC) [file pone.0006826.s004.doc]

| Probe sets | | | | | | Average Signals | | | |
| --- | --- | --- | --- | --- | --- | --- | --- | --- | --- |
| Present | % | Absent | % | Marginal | % | Present | Absent | Marginal | Overall |
| 22666 | 41.50% | 31050 | 56.80% | 959 | 1.80% | 719.8 | 47.4 | 131.7 | 327.6 |
| 22321 | 40.80% | 31400 | 57.40% | 954 | 1.70% | 737.9 | 46.1 | 132.8 | 330 |
| 25138 | 46.00% | 28658 | 52.40% | 879 | 1.60% | 682.5 | 35.8 | 103 | 334.2 |
| 24829 | 45.40% | 28996 | 53.00% | 850 | 1.60% | 656 | 36.1 | 104.8 | 318.7 |
| 24301 | 44.40% | 29445 | 53.90% | 929 | 1.70% | 652.9 | 44.5 | 131.4 | 316.4 |
| 20850 | 38.10% | 32829 | 60.00% | 996 | 1.80% | 790.8 | 60.6 | 172.7 | 341.1 |
| 23377 | 42.80% | 30346 | 55.50% | 952 | 1.70% | 690.6 | 45.9 | 135.5 | 323.1 |
| 24527 | 44.90% | 29213 | 53.40% | 935 | 1.70% | 648 | 42.1 | 118.9 | 315.2 |
| 26122 | 47.80% | 27683 | 50.60% | 870 | 1.60% | 619.3 | 34.5 | 97.2 | 314.9 |
| 23846 | 43.60% | 29902 | 54.70% | 927 | 1.70% | 654.8 | 43.7 | 118.9 | 311.5 |
| 23355 | 42.70% | 30437 | 55.70% | 883 | 1.60% | 687.4 | 45.5 | 128.5 | 321.1 |
| 25868 | 47.30% | 27914 | 51.10% | 893 | 1.60% | 678.1 | 34.5 | 101.6 | 340.1 |
| 25855 | 47.30% | 27916 | 51.10% | 904 | 1.70% | 627.5 | 33.2 | 91.9 | 315.2 |
| 22960 | 42.00% | 30793 | 56.30% | 922 | 1.70% | 699.4 | 47.8 | 144.6 | 323 |
| 25120 | 45.90% | 28651 | 52.40% | 904 | 1.70% | 680.1 | 37.3 | 109.1 | 333.8 |
| 26606 | 48.70% | 27181 | 49.70% | 888 | 1.60% | 591.9 | 28.3 | 80.4 | 303.4 |
| 26591 | 48.60% | 27235 | 49.80% | 849 | 1.60% | 629.5 | 26.4 | 84.3 | 320.6 |
| 24738 | 45.20% | 29038 | 53.10% | 899 | 1.60% | 683.8 | 34.6 | 105.9 | 329.5 |
| 22171 | 40.60% | 31529 | 57.70% | 975 | 1.80% | 733.3 | 47.9 | 150.6 | 327.7 |
| 24199 | 44.30% | 29501 | 54.00% | 975 | 1.80% | 668.3 | 40.5 | 122.8 | 319.9 |
| 24252 | 44.40% | 29547 | 54.00% | 876 | 1.60% | 650.3 | 36.4 | 115.5 | 310 |
| 26878 | 49.20% | 27007 | 49.40% | 790 | 1.40% | 591.8 | 27.6 | 77.7 | 305.6 |
| 26150 | 47.80% | 27691 | 50.60% | 834 | 1.50% | 599.3 | 32.2 | 99.7 | 304.5 |
| 23791 | 43.50% | 29997 | 54.90% | 887 | 1.60% | 645.8 | 42.7 | 128.6 | 306.5 |
| 23086 | 42.20% | 30669 | 56.10% | 920 | 1.70% | 709.8 | 43.5 | 136.9 | 326.4 |
| 25457 | 46.60% | 28416 | 52.00% | 802 | 1.50% | 625.1 | 29.3 | 87.5 | 307.6 |
| 24250 | 44.40% | 29533 | 54.00% | 892 | 1.60% | 660.5 | 36.9 | 106.2 | 314.7 |
| 27320 | 50.00% | 26588 | 48.60% | 767 | 1.40% | 560.7 | 25.1 | 73.5 | 293.4 |
| 22240 | 40.70% | 31499 | 57.60% | 936 | 1.70% | 729.9 | 46.3 | 139.5 | 326 |
| 27216 | 49.80% | 26615 | 48.70% | 844 | 1.50% | 575.4 | 29.5 | 84.4 | 302.1 |

Supplementary Table 4A. Overview of detected probes and signal strengths.

| RAW Noise | Scale Factor | Norm Factor | Background | | | | Noise | | | |
| --- | --- | --- | --- | --- | --- | --- | --- | --- | --- | --- |
| (Q) | (SF) | (NF) | Avg | Std | Min | Max | Avg | Std | Min | Max |
| 2.12 | 3.686 TGT Value: 200 | 1 | 59.18 | 0.71 | 57.3 | 60.8 | 3.13 | 0.11 | 2.9 | 3.7 |
| 2.36 | 3.269 TGT Value: 200 | 1 | 65.6 | 0.9 | 64 | 68.5 | 3.6 | 0.4 | 3.2 | 6.3 |
| 1.76 | 3.758 TGT Value: 200 | 1 | 48.5 | 0.7 | 46 | 50.4 | 2.7 | 0.4 | 2.3 | 5.1 |
| 2.33 | 2.921 TGT Value: 200 | 1 | 66.3 | 0.9 | 64 | 68.8 | 3.4 | 0.1 | 3.2 | 3.6 |
| 2.51 | 3.111 TGT Value: 200 | 1 | 67.9 | 0.9 | 66 | 70.5 | 3.7 | 0.2 | 3.4 | 5.2 |
| 2.14 | 4.320 TGT Value: 200 | 1 | 59.6 | 0.8 | 57 | 61.6 | 3.2 | 0.1 | 2.8 | 3.6 |
| 2.71 | 3.247 TGT Value: 200 | 1 | 73.2 | 1 | 72 | 76.3 | 3.9 | 0.1 | 3.5 | 4.3 |
| 2.82 | 2.581 TGT Value: 200 | 1 | 77.7 | 1.1 | 75 | 80 | 4.5 | 0.3 | 4.1 | 6.1 |
| 2.7 | 2.305 TGT Value: 200 | 1 | 73.4 | 1 | 72 | 76.2 | 4.1 | 0.1 | 3.8 | 4.4 |
| 2.79 | 2.758 TGT Value: 200 | 1 | 73.1 | 1.2 | 71 | 77.2 | 4.2 | 0.1 | 3.9 | 4.6 |
| 2.76 | 2.998 TGT Value: 200 | 1 | 75.9 | 0.9 | 74 | 79.1 | 4 | 0.1 | 3.7 | 4.3 |
| 2.21 | 2.895 TGT Value: 200 | 1 | 61.3 | 0.8 | 59 | 63.2 | 3.2 | 0.1 | 3.1 | 3.4 |
| 2.47 | 2.391 TGT Value: 200 | 1 | 69.3 | 1.1 | 66 | 72.6 | 3.6 | 0.1 | 3.2 | 3.9 |
| 2.25 | 3.593 TGT Value: 200 | 1 | 61.8 | 0.7 | 60 | 63.6 | 3.2 | 0.1 | 3 | 3.6 |
| 2.46 | 2.870 TGT Value: 200 | 1 | 68.7 | 0.9 | 66 | 71.1 | 3.6 | 0.1 | 3.4 | 3.9 |
| 2.81 | 1.359 TGT Value: 200 | 1 | 80.4 | 1.1 | 78 | 83.7 | 4.4 | 0.2 | 4 | 4.8 |
| 2.08 | 1.945 TGT Value: 200 | 1 | 56.3 | 0.7 | 55 | 58 | 3.1 | 0.1 | 2.8 | 3.7 |
| 2.24 | 2.592 TGT Value: 200 | 1 | 60.8 | 0.7 | 59 | 62.7 | 3.3 | 0.2 | 3 | 4 |
| 2.18 | 3.789 TGT Value: 200 | 1 | 58.9 | 0.7 | 58 | 60.6 | 3 | 0.1 | 2.7 | 3.2 |
| 2.29 | 2.782 TGT Value: 200 | 1 | 63.2 | 1.2 | 61 | 66.3 | 3.3 | 0.1 | 3.1 | 3.5 |
| 2.19 | 3.306 TGT Value: 200 | 1 | 62 | 0.9 | 59 | 64 | 3.1 | 0.1 | 2.8 | 3.3 |
| 2.61 | 2.187 TGT Value: 200 | 1 | 75.6 | 1.1 | 73 | 77.8 | 3.9 | 0.1 | 3.7 | 4.4 |
| 2.48 | 2.301 TGT Value: 200 | 1 | 71.6 | 1 | 69 | 73.3 | 3.9 | 0.3 | 3.4 | 5.6 |
| 2.63 | 3.032 TGT Value: 200 | 1 | 79.4 | 1.1 | 77 | 81.7 | 4.2 | 0.5 | 3.6 | 7.2 |
| 2.15 | 3.845 TGT Value: 200 | 1 | 63.6 | 0.8 | 62 | 64.8 | 3.1 | 0.2 | 2.8 | 4.2 |
| 2.82 | 2.181 TGT Value: 200 | 1 | 84.5 | 1.2 | 81 | 86.9 | 4.2 | 0.1 | 3.9 | 4.5 |
| 2.61 | 3.002 TGT Value: 200 | 1 | 79.4 | 1 | 76 | 80.8 | 3.8 | 0.2 | 3.5 | 4.5 |
| 3.07 | 1.505 TGT Value: 200 | 1 | 94.9 | 1.3 | 92 | 96.3 | 4.9 | 0.1 | 4.5 | 5.2 |
| 2.72 | 3.312 TGT Value: 200 | 1 | 80.1 | 0.9 | 78 | 82.4 | 4 | 0.1 | 3.7 | 4.3 |
| 2.62 | 1.991 TGT Value: 200 | 1 | 78.7 | 1.2 | 75 | 80.3 | 3.9 | 0.1 | 3.6 | 4.2 |

Supplementary Table 4B Overview background and noise levels.

Table S4. Overview of detected probes, signal strengths, background and noise levels.
