# Supplementary material for: Comprehensive mRNA Expression Profiling Distinguishes Tauopathies and Identifies Shared Molecular Pathways
Source: PLoS One. 2009 Aug 28;4(8):e6826. doi: 10.1371/journal.pone.0006826 (PMC2729393; doi:10.1371/journal.pone.0006826)
Supplement: Table S5 — Primer sequences of primers used for quantitative reverse transcriptase confirmation of the microarray data. (0.04 MB DOC) [file pone.0006826.s005.doc]

| Gene | forward | reverse |
| --- | --- | --- |
| MOXD1 | tcagcctgccagtgaatgtga | tgccacacaccaaaggttctg |
| ZNF589 | ggctgaagcagaagatcaacga | tggtggtctctggaacaggcta |
| FOXC1 | ctacatcgcgctcatcaccat | ctgcttgttgtcccggtagaag |
| SASH1 | agctggtaaactccactcgcag | aggaccggcgaattctcaa |
| ACAD2 | ggtgatggaggagatatcccga | cctcattcccattgcgtacaag |
| SEPT2 | gcccttagatgtggcgtttatg | gaatccttttcttcagccgctc |
| PNPLA2 | atccaggccaatgtctgca | ggttgtctgaaatgccacca |
| TNPO1 | atgctggactcttagccgctat | tctggcggctggctga |
| CBL | gtgtgaccaccccaaaatcaaa | gctatcaatctgctggtcgcaa |
| GOLPH4 | agcaggaggacaatgttgatga | cagcattatgctccagttccc |
| NRD1 | tttgacttccttcgaaccaagc | tatttggttgcctgagtcccc |
| PARD3 | ttgccgaggtgactttgaatg | ttgtcgatggcagctctgaag |
| PTEN | tcagtggcggaacttgcaa | catgaacttgtcttcccgtcg |
| NPIP | aagaaagcctctttgcaaccg | ggtggcccatcctgtttttt |
| BTBD14A | atcccaagctctactcggaagg | ttatgcttcacccctgcacac |
| CENTB5 | tgagcttggaaacagcgct | gtatttgtccttgatccaggcc |
| HPRT | ATGGGAGGCCATCACATTGT | ATGTAATCCAGCAGGTCAGCAA |
| β-actin | GCTCCTCCTGAGCGCAAG | CATCTGCTGGAAGGTGGACA |

Table S5. Primer sequences of primers used for quantitative reverse transcriptase confirmation of the microarray data.
