# Supplementary material for: Comprehensive mRNA Expression Profiling Distinguishes Tauopathies and Identifies Shared Molecular Pathways
Source: PLoS One. 2009 Aug 28;4(8):e6826. doi: 10.1371/journal.pone.0006826 (PMC2729393; doi:10.1371/journal.pone.0006826)
Supplement: Supplementary File S1 — Ethical Declaration of The Netherlands Brain Bank (0.09 MB PDF) [file pone.0006826.s006.pdf]

Meibergdreef 47
1105 BA Amsterdam
The Netherlands
T +31 20 566 5499
F +31 20 691 8466
www.brainbank.nl

Netherlands Brain Bank

ETHICAL DECLARATION
OF THE NETHERLANDS BRAIN BANK

Whereas the Netherlands Brain Bank (NBB) is engaged in the process of recruiting and disseminating post-mortem human tissue for scientific research, it abides the Dutch law and regulations for obtaining and using human tissue for research and adheres to the following Code of Conduct:

1. Written informed consent is obtained by the NBB from both the donor and the next of kin (or a designated confidant in the absence of family) for the following:

a. To perform an autopsy during which the brain and spinal cord are removed to be stored and distributed for scientific research purposes.
b. To view and process the medical file for scientific research purposes.

2. In case a power of attorney has been given by a person, the holder of this power of attorney can sign the consent forms on behalf of the person who, for reasons of mental or physical health, is no longer capable to give the permission in person. The consent of the holder of the power of attorney covers the areas mentioned in 1. and 2.

3. All tissues and remains are handled with utmost respect and care.

4. The tissue is accompanied by anonymized donor information. It is not allowed to carry out any procedures by which the identity of the donor could be derived.

5. All tissue recipients are informed on the possible hazardous nature of the tissues and sign for handling all material with the necessary safety methods.

6. All recipients are responsible to return unused tissues to the NBB and dispose of tissue rests according to local safety rules for the disposal of human remains.

7. All tissue is supplied for scientific research and is not to be passed on to a third party without prior written permission from the NBB. It is strictly forbidden to use the NBB tissue for commercial purposes.
